# Biocatalyzed and Photochemical Formal [2+2] Cycloaddition of Euphoboetirane A: A Route to a Fused Pentacyclic Diterpene Skeleton

**DOI:** 10.1021/acs.orglett.4c04724

**Published:** 2025-02-02

**Authors:** Fátima Vela Benavides, Marija Kirić, Felipe Escobar-Montaño, Antonio J. Macías-Sánchez, Hernando Bolivar-Anillo, José M. Botubol-Ares, Rosa Durán-Patrón, Rosario Hernández-Galán

**Affiliations:** ‡ Departamento de Química Orgánica, Facultad de Ciencias, 16727 Universidad de Cádiz, Puerto Real, 11510 Cádiz, Spain; Ψ Instituto Universitario de Investigación en Biomoléculas, 16727 Universidad de Cádiz, Puerto Real, 11510 Cádiz, Spain; ⊥ Laboratorio de Investigación en Microbiología, Facultad de Ciencias Básicas y Biomédicas, Universidad Simón Bolivar, Barranquilla 080002, Colombia; § Instituto Universitario de Investigación Vitivinícola y Agroalimentaria, 16727 Universidad de Cádiz, Puerto Real, 11510 Cádiz, Spain

## Abstract

Cycloeuphoboetirane A (**2**), a new diterpene with an unprecedented pentacyclic skeleton, has been obtained from lathyrane euphoboetirane A (**1**). A formal intramolecular [2+2] cycloaddition reaction catalyzed by *Sordaria tomento-alba* ST1-UCA is involved, providing new evidence for the existence of enzymes capable of catalyzing this reaction. A biomimetic photochemical conversion was also achieved by benzophenone with light-emitting diode irradiation.

## Graphical abstract

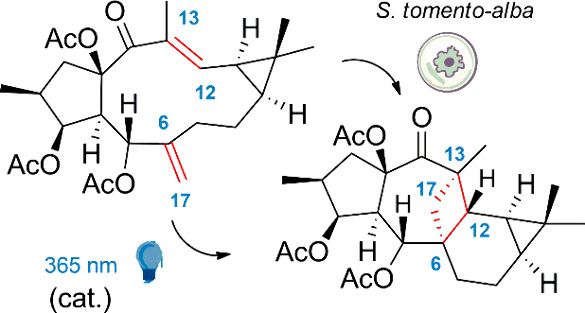

Cyclobutane rings are structural features widely present in many biologically active natural products of medicinal value.^1–5^ Synthetic efforts to prepare four-membered rings have recently emerged, with the [2+2] cycloaddition reaction still being a powerful approach to access novel scaffolds.^6,7^

Over the past decade, enzymes capable of catalyzing [4+2] cycloadditions, such as Diels–Alderases, have been discovered in the biosynthesis of natural products. However, enzymes catalyzing [2+2] cycloadditions have remained elusive.^8–11^ Recent findings suggest that certain [4+2] cyclases, such as those involved in the biosynthesis of pyrroindomycins, can also catalyze [2+2] cycloadditions.^12,13^ The study of the cyclase responsible for the biosynthesis of pyrrolosporin A in *Micromonospora* sp. C39217-R109-7 has provided mechanistic insights into its versatile catalytic activity. A common diradical intermediate can exploit divergent reaction pathways, leading to either [4+2] or [2+2] cycloaddition products, depending on kinetic and thermodynamic factors.

Lathyrane diterpenes are considered to be biogenetic precursors of diverse diterpenoid scaffolds. Transannular cyclization of lathyranes is a promising approach to generating chemical diversity. However, this strategy remains largely unexplored with only a few examples.^14–27^ Similarly, only a few lathyrane diterpenoid biotransformations have been described.^28–32^ These biotransformations primarily involved regioselective oxidation at non-activated carbons, cyclopropane ring opening, and isomerization of the Δ^12^ double bond.

Previously, we isolated gaditanone, a diterpene containing a four-membered ring, from *Euphorbia gaditana*.^33^ We proposed a biosynthetic pathway involving an intramolecular [2+2] photocycloaddition of a suitable jatrophadienone. This hypothesis was further validated by synthetic and computational studies, which support a nonconcerted photochemical mechanism via a diradical intermediate.^33,34^

Sordaricins are antifungal ditepenes produced by several microorganisms, including *Sordaria* spp.^35–37^ A key step in sordaricin biosynthesis involves a Diels–Alder [4+2] cycloaddition catalyzed by an enzyme identified in the *Sordaria araneosa* genome.^38,39^ Our research group recently identified *Sordaria tomento-alba* ST1-UCA, an endophytic fungus isolated from *Gliricidia sepium*, which exhibits significant antifungal activity against *Botrytis cinerea*.^40^ Although the direct identification of sordaricins in this strain remains elusive, their potential production may contribute to the observed antifungal activity.

On the basis of these precedents, and to delve deeper into the possible existence of enzymes capable of catalyzing [2+2] cycloaddition reactions, we chose euphoboetirane A (**1**), a lathyrane-type diterpene containing two double bonds, as a suitable substrate to investigate microbially mediated [2+2] cycloaddition reactions.^41,42^

Initially, *S. tomento-alba* was cultured on PDA plates for 14 days. Then, it was transferred to flasks containing 200 mL of PDB medium and incubated while being shaken for 3 days. Euphoboetirane A (**1**) was added to each flask, and incubation was continued for a further 10 days. The fermentation broth was filtered and extracted, and the resulting extract was analyzed by TLC and HPLC, revealing the presence of two minor compounds in low yield: cycloeuphoboetirane A (**2**) and **3** (Figure 1). However, the starting material was not recovered.

**Figure 1:**
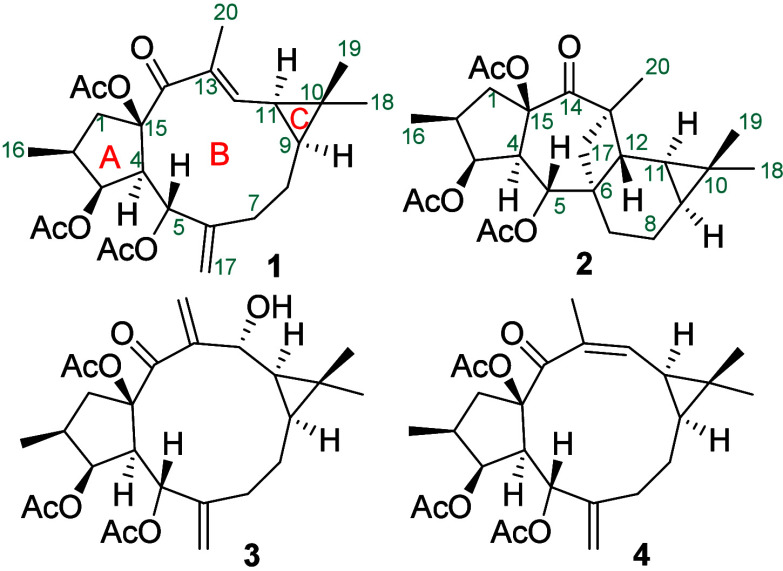
Chemical structures of **1** and its biotransformation compounds (**2**–**4**) obtained by *S. tomento*-*alba* ST1-UCA.

To enhance the biotransformation, **1** was incubated with resting cell cultures of *S. tomento-alba* in Czapek–Dox medium. The progress of the biotransformation was monitored by HPLC analysis at 1, 3, and 5 days (Table S2 and Figures S2–S4). The highest yield for compound **2** (0.6% yield) was achieved after incubation for 3 days. During this time, the starting material was recovered, and known compound **4** ^31^ was also produced (38% yield).

Compound **2** showed a molecular formula of C_26_H_36_O_7_, deduced from the [M + Na]^+^ molecular ion (*m*/*z* 483.2374, calcd for C_26_H_36_O_7_Na), exhibiting the same degree of unsaturation as **1**. However, the characteristic olefinic signals in the nuclear magnetic resonance (NMR) spectra of **1** had disappeared,^43^ indicating that two new carbocycles might have been formed through a formal intramolecular [2+2] cycloaddition (Table S1). Two isolated spin systems were inferred from the ^1^H–^1^H COSY correlations of H_2_-1/H-2­(H_3_-16)/H-3/H-4/H-5 and H_2_-7/H_2_-8/H-9/H-11/H-12 (Figure 2). Moreover, a signal at δ_H_ 2.92 (d, *J* = 4.4 Hz, H-12) was revealed to be coupled to diastereotopic proton H_2_-17 (δ_H_ 1.92, dd, *J* = 13.6, 4.8 Hz) in the one-dimensional (1D) TOCSY spectrum. This coupling is characteristic of a W coupling in rigid strained cyclobutane rings.^44^ Thereby, two plausible cyclobutane isomers were considered for the formal [2+2] cycloaddition. HMBC correlations from H_2_-17 to C-5–C-7, C-12–C-14, and C-20 and from H-12 to C-5, C-6, C-10, C-13, C-14, and C-20, along with the 1D NOESY correlations between H-5/H-12/H_3_-19 and H-11/H-9/H_3_-18/H_3_-20, confirmed that the formal [2+2] cycloaddition had occurred between C-6/C-12 and C-13/C-17 (Figure 2). Accordingly, the structure of cycloeuphoboetirane A was assigned to compound **2**.

**Figure 2:**
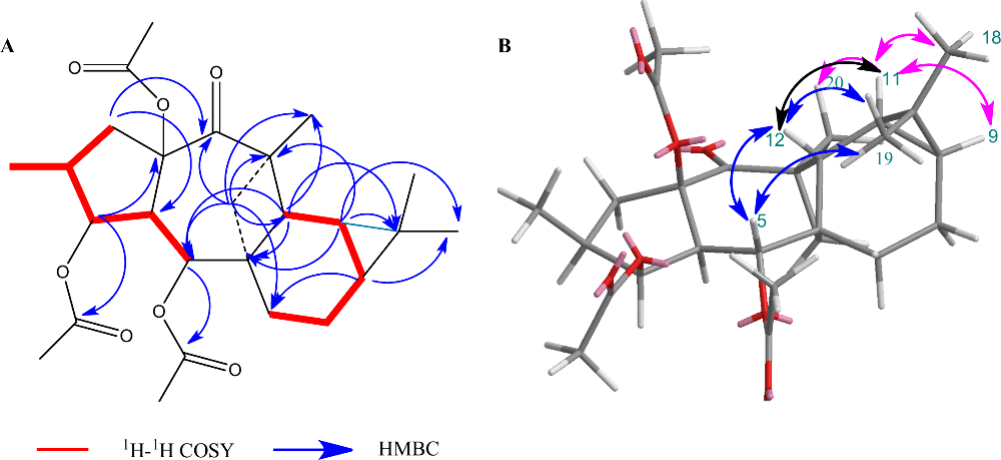
Key two-dimensional (2D) NMR correlation of **2**. (A) COSY and HMBC correlations. (B) 1D and 2D NOESY correlations. β-Face correlations are colored blue, and α-face correlations purple. Correlations involving nuclei in the α-face and β-face are colored black.

Compound **3** exhibited a molecular formula of C_26_H_36_O_8_, indicating the presence of an additional hydroxyl group. The absence of the characteristic olefinic signal for H-12 of **1** in the ^1^H NMR spectrum and the appearance of a new signal at δ_H_ 4.76 (d, *J* = 10.1 Hz) (Table S1) suggested hydroxylation at this position. HMBC correlations from H-12 to C-11, C-13, C-14, and C-20 supported this assignment. Additionally, two new olefinic exomethylene proton signals at δ_H_ 5.19 and 4.81 were observed, indicating isomerization of the Δ^12^ to Δ^13(20)^ double bond, as supported by HMBC correlations from these protons to C-12–C-14. The α-configuration of the hydroxyl group at C-12 was determined by the 1D NOESY correlation of H-12/H-7β/H_3_-19 and H-9/H-11/H_3_-18/H-8α/H-7α/H-20a (Figure S1).

Compound **4** was identified by comparison of its spectroscopic data to those of (12*Z*)-euphoboetirane A, previously obtained through biotransformation of **1** by *Streptomyces puniceus* BC-5GB.11.^31^

A biomimetic semisynthesis of **2** from **1** was investigated. We hypothesized that photoactivation of the Δ^12^ double bond, followed by hydrogen abstraction at C-7, could trigger the formal [2+2] cycloaddition (Table 1 and Table S3).

**Table 1:**
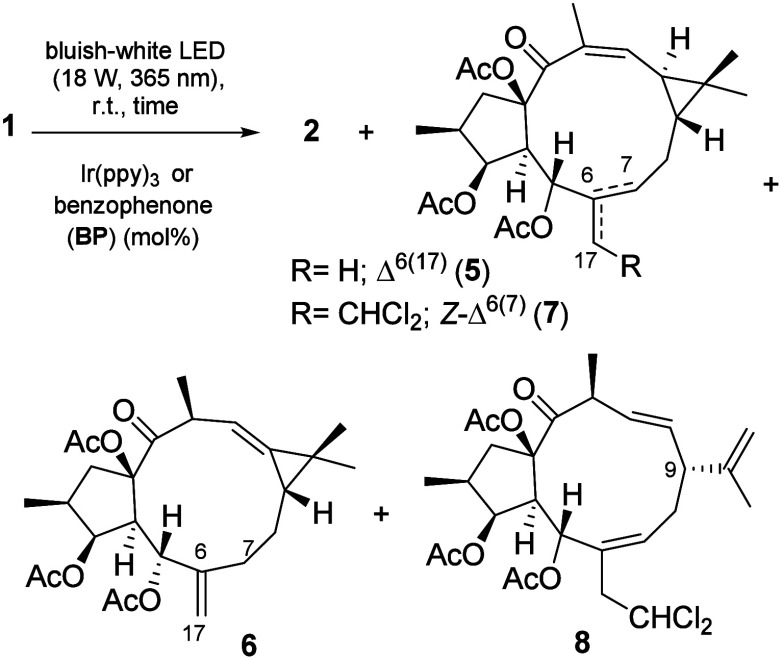
Optimization of the Reaction Conditions^a^

entry	solvent	photocatalyst (mol %)	time (h)	**2**/**5**/**6**/**7**/**8** ^b^
1	DMF	Ir(ppy)_3_ (2.5)	24	0/82/0/0/0
2	DMF	Ir(ppy)_3_ (2.5)	48	0/51/0/0/0
3	DMF	Ir(ppy)_3_ (30)	24	8/47/0/0/0
4	DMF	BP (30)	24	0/72/0/0/0
5	DMF	BP (30)	48	14/20/17/0/0
6	DMF	BP (60)	24	35/41/0/0/0
7	DMF	BP (60)	48	55/0/0/0/0
8	DCM	BP (30)	24	0/46/0/32/0
9	DCM	BP (60)	24	0/0/0/47/28
10	DCM	BP (60)	48	0/0/0/16/34

a Reactions were conducted with **1** (0.04 mmol), a solvent (2 mL), photocatalyst Ir­(ppy)_3_ or BP, and irradiation with an 18 W bluish-white LED (365 nm).

b Isolated yields.

Inspired by the work of Wang et al. on the photocatalytic isomerization of lathyrane-type diterpenoids,^27^ we employed Ir­(ppy)_3_ as a photocatalyst. Irradiation of **1** with 2.5 mol % Ir­(ppy)_3_ under a bluish-white light-emitting diode (LED) exclusively yielded lathyrane **5** (Table 1, entries 1 and 2).^27^ The ^1^H NMR spectrum of **5** exhibited temperature-dependent signal splitting (Figure S31).^33^ Hydrolysis under basic conditions produced compound **5a**, which disappeared via this atropoisomeric phenomenon. Analysis of the 1D NOESY spectra of **5a** confirmed its structure as (9*R*,12*Z*)-euphoboetirane A (Figure S40a–e).^33^

Increasing the amount of Ir­(ppy)_3_ to 30 mol % afforded the desired formal [2+2] cycloaddition product **2** in low yield (Table 1, entry 3). Then, we explored the use of photoexcited benzophenone as the photocatalyst.^45–47^ The reaction time was found to significantly influence the outcome of the reaction. Initially, using 30 mol % benzophenone (BP) in DMF for 24 h led exclusively to compound **5** (Table 1, entry 4). Extending the reaction time to 48 h resulted in a mixture of **2** and **5**, along with 17% compound **6** (Table 1, entry 5). The formation of the latter may be explained by hydrogen abstraction at position C-11 (Scheme S1). Increasing the loading to 60 mol % gave an improved yield of **2**, reaching 55% after 48 h (Table 1, entries 6 and 7). Switching the solvent to DCM did not afford **2**. Instead, a mixture of **5** and compounds **7** and **8** was formed (Table 1, entries 8–10).

After optimizing the reaction conditions, we applied them to compound **5**, affording compound **2** in 24% yield (Table S3, entry 14). This result suggests that hydrogen abstraction at C-7 occurs from compound **5**. On the basis of our observations, a plausible mechanistic pathway is depicted in Scheme 1.

**Scheme 1:**
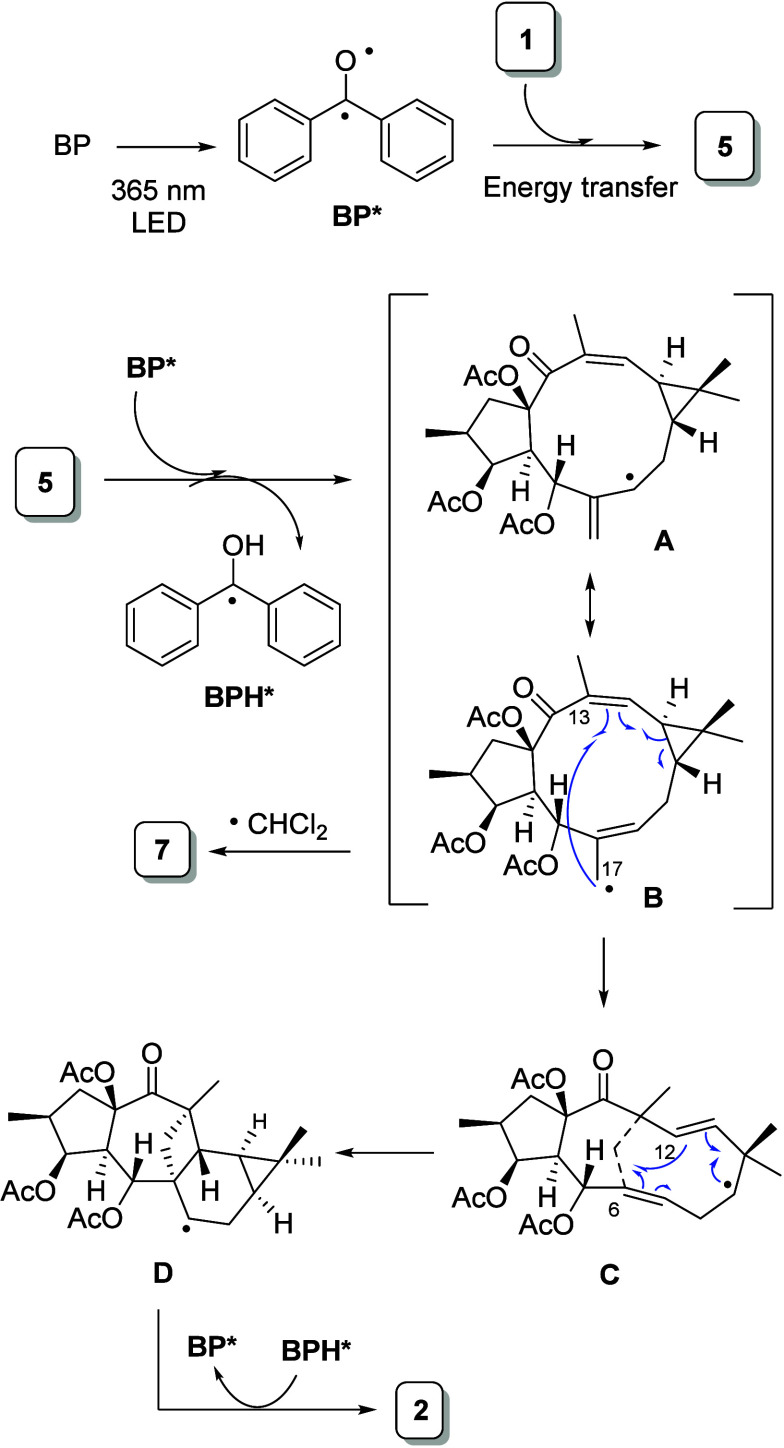
Proposed Mechanism for the Formal [2+2] Cycloaddition of **1**

The photocatalytic process is initiated with the excitation of benzophenone to its photoexcited state (BP*). The transfer of energy from BP* to Δ^12^ produces compound **5**.^48^ Subsequent hydrogen abstraction at allylic position C-7 would generate radical intermediates **A** and **B**. The latter can develop two different pathways depending on the solvent used. Radical **B** is trapped by a CHCl_2_ radical to produce compound **7** when the reaction is carried out in DCM. In DMF, homolytic cleavage of the cyclopropane ring followed by coupling between C-17 and C-13 generates intermediate **C**. Further cyclopropane ring regeneration and intramolecular coupling between C-12 and C-6 affords **D**. Finally, abstraction of hydrogen from BPH* produces compound **2** (Scheme 1 and Scheme S1).

In conclusion, cycloeuphoboetirane A (**2**), featuring an unprecedented 5/7/4/6/3 fused-ring skeleton, was isolated from the microbial transformation of **1** by the endophytic fungus *S. tomento-alba* through a formal intramolecular [2+2] cycloaddition reaction. To the best of our knowledge, this is the first reported instance of a microbiologically catalyzed formal [2+2] cycloaddition reaction. Additionally, the chemical conversion of **1** into **2** was achieved by a formal intramolecular [2+2] photochemical cycloaddition using benzophenone as a photocatalyst.

## Supplementary Material



## Data Availability

The data underlying this study are available in the published article and its Supporting Information.

## Notes

Detailed experimental procedures, full spectroscopic data for all new compounds, and copies of 1D and 2D NMR spectra for **2**–**8** (PDF)
