# Supplementary material for: Biocatalyzed and Photochemical Formal [2+2] Cycloaddition of Euphoboetirane A: A Route to a Fused Pentacyclic Diterpene Skeleton
Source: Org Lett. 2025 Feb 2;27(6):1407–11. doi: 10.1021/acs.orglett.4c04724 (PMC12426989; doi:10.1021/acs.orglett.4c04724)
Supplement: Supplementary file 1 [file ol4c04724_si_001.pdf]

Biocatalyzed and Photochemical Formal [2+2] Cycloaddition of Euphoboetirane A: A Route to a Fused Pentacyclic Diterpene Skeleton

Fátima Vela Benavides[‡], Marija Kirić[‡], Felipe Escobar-Montaño[‡], Antonio J. Macías-Sánchez[‡,Ψ], Hernando Bolivar-Anillo,[⊥] José M. Botubol-Ares[‡,§,*], Rosa Durán-Patrón[‡,Ψ], Rosario Hernández-Galán[‡,Ψ,*].

[‡]Departamento de Química Orgánica, Facultad de Ciencias, Universidad de Cádiz, Puerto Real, 11510 Cádiz, Spain. [Ψ]Instituto Universitario de Investigación en Biomoléculas, Universidad de Cádiz, Puerto Real,11510 Cádiz, Spain. [⊥]Laboratorio de Investigación en Microbiología, Facultad de Ciencias Básicas y Biomédicas, Universidad Simón Bolivar, Barranquilla, Colombia. [§]Instituto Universitario de Investigación Vitivinícola y Agroalimentaria, Universidad de Cádiz, Puerto Real, 11510 Cádiz, Spain.

***Corresponding authors:**

Prof. Dr. Rosario Hernández-Galán (rosario.hernandez@uca.es)

Dr. José M. Botubol-Ares (josemanuel.botubol@uca.es)

Table of Contents

1. General experimental procedures S4

2. Extraction and isolation of euphoboetirane A (1) S4

3. Biotransformation of euphoboetirane A (1) by *Sordaria tomento-alba* S4

Table S1. ^{1}H (700 MHz) and ^{13}C NMR (175 MHz) spectroscopic data for compounds **2** and **3** in $CDCl_3$ S5

Table S2. HPLC analysis of biotransformation of compound **1** using resting cell culture S6

Figure S1. Key 2D NMR correlation of **3** S6

Figure S2. HPLC analysis of biotransformation of **1** using resting cell culture (day 1). S7

Figure S3. HPLC analysis of biotransformation of **1** using resting cell culture (day 3) S7

Figure S4. HPLC analysis of biotransformation of **1** using resting cell culture (day 5) S8

4. General procedure for the photocatalyzed formal [2+2] intramolecular cycloaddition S8

Table S3. Optimization of the reaction conditions for the photocatalyzed formal [2+2] intramolecular cycloaddition of **1** S9

Figure S5. Key ^{1}H-^{1}H COSY and HMBC correlations for compounds **5a** and **6-8**.....S10

Figure S6. Selected 1D and 2D NOESY correlations for compounds **5a** and **6** S10

Figure S7. Selected 1D and 2D NOESY correlations for compounds **7** and **8** S11

Table S4. ^{1}H NMR Spectroscopic data for compounds **5a** (500 MHz, CD_3OD), **7** (500 MHz, $CDCl_3$), **6**, and **8** (700 MHz, $CDCl_3$) (δ_H in ppm, *J* in Hz).....S11

Table S5. ^{13}C NMR Spectroscopic data for compounds **5a** (125 MHz, CD_3OD), **7** (125 MHz, $CDCl_3$), **6**, and **8** (175 MHz, $CDCl_3$) (δ_C in ppm). S12

Scheme S1. Proposed mechanism for the formal [2+2] cycloaddition of **1** and its transformation into compounds **5-8** S13

5. References S13

6. NMR, ECD and HRMS spectra S14

Figure S8. ^{1}H NMR spectrum (700 MHz) of compound **2** in $CDCl_3$ S14

Figure S9. ^{13}C NMR spectrum (175 MHz) of compound **2** in $CDCl_3$ S15

Figure S10. gCOSY spectrum of compound **2** S16

Figure S11. gHSQC spectrum of compound **2** S17

Figure S12. gHMBC (3 Hz) spectrum of compound **2** S18

Figure S13. gHMBC (5 Hz) spectrum of compound **2** S19

Figure S14. gHMBC (10 Hz) spectrum of compound **2** S20

Figure S15. 2D NOESY spectrum of compound **2** S21

Figures S16a-f. 1D NOESY spectra of compound **2** S22-27

Figures S17a-c. 1D TOCSY spectrum of compound **2** S28-30

Figure S18. HRMS of compound **2** S31

Figure S19. ECD spectrum of compound **2** S32

Figure S20. ^{1}H NMR spectrum (700 MHz) of compound **3** in $CDCl_3$ S33

Figure S21. ^{13}C NMR spectrum (175 MHz) of compound **3** in $CDCl_3$ S34

Figure S22. gCOSY spectrum of compound **3** S35

Figure S23. gHSQC spectrum of compound **3** S36

Figure S24. gHMBC spectrum of compound **3** S37

Figures S25a-d. 1D NOESY spectra of compound **3** S38-41

Figure S26. HRMS of compound **3** S42

Figure S27. ECD spectrum of compound **3** S43

Figure S28. ^{1}H NMR spectrum (400 MHz) of compound **4** in $CDCl_3$ S44

Figure S29. ^{13}C NMR spectrum (100 MHz) of compound **4** in $CDCl_3$ S45

Figure S30. ^{1}H NMR spectrum (400 MHz) of mixture of atropoisomers **5** in $CDCl_3$ S46

Figure S31. ^{1}H NMR spectrum (400 MHz) of mixture of atropoisomers **5** in DMSO-d_6 (25ºC for upper spectrum and 80ºC for lower spectrum) S47

Figure S32. HRMS of compound **5** S48

Figure S33. ^{1}H NMR spectrum (500 MHz) of compound **5a** in $CDCl_3$ S49

Figure S34. ^{1}H NMR spectrum (500 MHz) of compound **5a** in CD_3OD S50

Figure S35. ^{13}C NMR spectrum (125 MHz) of compound **5a** in CD_3OD S51

Figure S36. gCOSY spectrum of compound **5a** in CD_3OD S52

Figure S37. gHSQC spectrum of compound **5a** in CD_3OD S53

Figure S38. gHMBC spectrum of compound **5a** in CD_3OD S54

Figure S39. 2D NOESY spectrum of compound **5a** in CD_3OD S55

Figures S40a-e. 1D NOESY spectra of compound **5a** in CD_3OD.................... S56-60

Figure S41. ECD spectrum of compound **5a**.................... S61

Figure S42. ^{1}H NMR spectrum (700 MHz) of compound **6** in $CDCl_3$ S62

Figure S43. ^{13}C NMR spectrum (175 MHz) of compound **6** in $CDCl_3$ S63

Figure S44. gCOSY spectrum of compound **6** S64

Figure S45. gHSQC spectrum of compound **6** S65

Figure S46. gHMBC spectrum of compound **6** S66

Figure S47. 2D NOESY spectrum of compound **6**.................... S67

Figures S48a-e. 1D NOESY spectra of compound **6** S68-72

Figure S49. HRMS of compound **6**.................... S73

Figure S50. ^{1}H NMR spectrum (500 MHz) of compound **7** in $CDCl_3$ S74

Figure S51. ^{13}C NMR spectrum (125 MHz) of compound **7** in $CDCl_3$ S75

Figure S52. gCOSY spectrum of compound **7** S76

Figure S53. gHSQC spectrum of compound **7** S77

Figure S54. gHMBC spectrum of compound **7** S78

Figure S55. 2D NOESY spectrum of compound **7**.................... S79

Figures S56a-c. 1D NOESY spectra of compound **7** S80-82

Figure S57. ECD spectrum of compound **7**.................... S83

Figure S58. HRMS of compound **7**.................... S84

Figure S59. ^{1}H NMR spectrum (700 MHz) of compound **8** in $CDCl_3$ S85

Figure S60. ^{13}C NMR spectrum (175 MHz) of compound **8** in $CDCl_3$ S86

Figure S61. gCOSY spectrum of compound **8** S87

Figure S62. gHSQC spectrum of compound **8** S88

Figure S63. gHMBC spectrum of compound **8** S89

Figures S64a-g. 1D NOESY spectra of compound **8** S90-96

Figure S65. HRMS of compound **8**.................... S97

1. General experimental procedures

All chemical purchased from commercial sources and used without further purification. Anhydrous DMF was stored over 4Å molecular sieves. Dichloromethane (DCM) was distilled under CaH_2. Irradiation of photochemical reactions were carried out using a EvoluChemTM Photoredox Box (US Patent 10,906,022). Light source was a HCK1012-01-002 EvoluChem blue LED (18W, 445 nm) and HCK1012-01-011 EvoluChem bluish-white LED (18W, 365 nm). The irradiation vessel material was borosilicate glass. The distance from the light source to the irradiation vessel was 3 cm with none filters used. Unless otherwise specified, reactions were carried out under an argon atmosphere in 10 mL glass tube. Reactions were monitored by thin layer chromatography (TLC) using hexane:ethyl acetate mixtures as eluent and vainilline solution to visualize the course of the reactions. Optical rotations were determined with a JASCO P-2000 polarimeter (JASCO, Tokyo, Japan). Infrared spectra were recorded on a PerkinElmer Spectrum BX FT-IR spectrophotometer (PerkinElmer, Waltham, CA, USA) and reported as wave number (cm^{-1}). ^{1}H and ^{13}C NMR measurements were recorded on Bruker NMR spectrometers (700, 500 or 400 MHz) (Billerica, Massachusetts, USA) with $SiMe_4$ as the internal reference. Chemical shifts are expressed in ppm (δ), referenced to $CDCl_3$ (Eurisotop, Saint-Aubiu, France, δ_H 7.25, δ_C 77.0) or CD_3OD (Eurisotop, Saint-Aubiu, France, δ_H 3.30, δ_C 49.0). COSY, TOCSY, HSQC, HMBC, and NOESY experiments were performed using a standard Bruker pulse sequence. Spectra were assigned using a combination of 1D and 2D techniques. HRMS was performed in a Q-TOF mass spectrometer (Xevo-G2-S QTOF; Waters, Manchester, UK) in the positive-ion ESI mode. TLC was performed on Merck Kiesegel 60 Å F_{254}, 0.25 mm layer thickness. Purification by column chromatography were performed using Silica gel 60 (60−200 µm, VWR). For further purification, a Merck-Hitachi Primade HPLC equipped with an UV−vis detector (Primaide 1410) and a refractive index detector (RI-5450) or a Merck-Hitachi LaChrom apparatus equipped with a UV−vis detector (L 4250) and a differential refractometer detector (RI-7490) (Merck, Darmstadt, Germany) were used. LiChroCART LiChrospher Si 60 (5 µm, 250 mm × 4 mm), LiChroCART LiChrospher Si 60 (10 µm, 250 mm × 10 mm), and ACE 5 SIL (5 µm, 250 mm × 4.6 mm id) columns were used for isolation experiments.

2. Extraction and isolation of euphoboetirane A (1)

Starting compound, euphoboetirane A (**1**), was isolated in gram amounts by column chromatography from the MeOH extract of the aerial parts of *Euphorbia boetica* following the procedure described in the literature.[1] The whole plants were collected at El Pinar del Hierro (Chiclana de la Frontera, Cádiz, Spain) in July 2023 with the permission of the national competent authorities (Dirección General de Biodiversidad, Bosques y Desertificación, Secretaría de Estado de Medio Ambiente, Ministerio para la Transición Ecológica y Reto Demográfico (reference number ESNC64) and Consejería de Agricultura, Ganadería Pesca y Desarrollo Sostenible-Delegación Territorial de Cádiz, Junta de Andalucía (reference number 201999901092011)). Fresh plants of *E. boetica* (3.2 kg) were frozen with liquid nitrogen, powdered, and extracted with MeOH (2.5 L) three times at room temperature for 24 h. The solvent was evaporated to yield a crude residue, which was suspended in water (1 L) and then extracted with *n*-hexane (1.5 L x 3). The residue (33.3 g) was purified by a silica gel column chromatography eluting with *n*-hexane:EtOAc (v/v 9:1 to 0:1) to afford 21 fractions, according to TLC analysis. Fractions 4-7 (3.55 g) were combined and purified by column chromatography using first a CH_2Cl_2:acetone gradient (v/v 1:0 to 49:1) and in further purification with a gradient mixture of *n*-hexane:EtOAc (v/v 9:1 to 0:1) to yield 2.0 g of pure euphoboetirane A (**1**).

3. Biotransformation of euphoboetirane A (1) by *Sordaria tomento-alba*

3.1 – Microorganism

The endophytic fungus *Sordaria tomento-alba* ST1-UCA was isolated from *Gliricidia sepium* in Barranquilla (Colombia) and identified by the Spanish Type Culture Collection (CECT).[2]

3.2- Whole cell experiment

The strain ST1-UCA was cultured in Petri dishes on Potato Dextrose Agar (PDA, Condalab, Madrid, Spain) at 25°C under white light (daylight lamp) for 14 days. Six mycelium plugs (1 cm diameter) from this culture were transferred to eighteen 500 mL-Erlenmeyer flasks containing 200 mL of sterilized Potato Dextrose Broth (PDB, Condalab, Madrid, Spain) medium. The flasks were then incubated at 25 °C and 200 rpm

under white light for 3 days. Subsequently, 300 µL of a DMSO solution of euphoboetirane A (**1**) was added to each flask at a final concentration of 85 ppm. The flasks were incubated under the conditions described above for further10 days. A flask containing 200 mL of sterilized PDB and 16.95 mg of **1** dissolved in 300 µL of DMSO was used as a product control and another with the same amount of medium, 6 plugs of mycelium and 300 µl of DMSO as a control of the microorganism. The broths were filtered and extracted with ethyl acetate (3x). The organic extracts were dried over anhydrous Na_2SO_4 and the solvents were removed under reduced pressure. The residues were solved in ethyl acetate and analyzed by TLC and HPLC. The analysis revealed the presence of two minor compounds; however, the starting material was not recovered. The purification of the obtained extract through column chromatography using a *n*-hexane:EtOAc gradient (v/v 9:1 to 0:1) yielding two fractions (F2 and F4), which were further purified by HPLC. Fractions F2 (53.32 mg) and F4 (57.21 mg) were purified by semipreparative HPLC, using *n*-hexane:EtOAc (85:15 and 70:30, respectively), to yield cycloeuphoboetirane A (**2**) (0.2 mg, 0.07%) and **3** (0.9 mg, 0.28%).

Table S1. ^{1}H (700 MHz) and ^{13}C NMR (175 MHz) spectroscopic data for compounds **2** and **3** in $CDCl_3$.

Position	**2**		**3**	
	**δ_H, mult (*J* in Hz)**	**δ_C, type**	**δ_H, mult (*J* in Hz)**	**δ_C, type**
1*α*	3.31, dd (15.6, 11.2)	41.2 CH_2	3.09, dd (16.0, 8.4)	46.9, CH_2
1*β*	1.54, dd (15,6, 6.8)		2.39, dd (16.0, 11.9)	
2	2.41-2.34, m	34.7, CH	2.56-2.50, m	37.7, CH
3	5.46, dd (5.9, 4.4)	76.2 CH	5.58, t (4.0)	76.5, CH
4	2.36, dd (10.5, 4.4)	52.7, CH	3.14, dd (10.5, 4.0)	53.7, CH
5	5.43, d (10.5)	72.7, CH	5.64, d (10.5)	74.2, CH
6	-	37.6, C	-	142.7, C
7*α*	1.24-1.19, m	24.3, CH_2	1.62-1.55, m	29.1, CH_2
7*β*	1.00-0.96, m		2.14-2.10, m	
8*α*	1.88-1.83, m	16.6, CH_2	1.92-1.85, m	22.1, CH_2
8*β*	1.79-1.74, m		0.73-0.66, m	
9	0.73-0.70, m	18.9, CH	0.61, dt (9.0, 2.1)	29.7, CH
10	-	16.0, C	-	19.5, C
11	0.61 d (9.0)	19.5, CH	0.85, dd (10.1, 9.0)	34.2, CH
12	2.92, d (4.8)	34.3, CH	4.76, d (10.1)	65.4, CH
13	-	50.5, C	-	148.8, C
14	-	207.4, C	-	192.4, C
15	-	92.2, C	-	92.5, C
16	0.95, d (6.9)	15.5 CH_3	1.03, d (6.5)	14.0, CH_3
17a	2.08, d (13.0)	37.1, CH_2	5.19, d (2.9)	116.8, CH_2
17b	1.92, dd (13.0, 4.8)		4.81, d (2.9)	
18	0.99, s	30.1, CH_3	1.10, s	28.9, CH_3
19	1.03, s	16.6, CH_3	1.15, s	14.9, CH_3
20a	1.34, s	20.0, CH_3	6.40, s	125.6, CH_2
20b			6.17, s	
O<u>C</u>O-3	-	170.9, C	-	170.5, C
OCO<u>Me</u>-3	2.09, s	20.9, CH_3	2.09, s	21.1, CH_3
O<u>C</u>O-5	-	170.2, C	-	169.5, C
OCO<u>Me</u>-5	1.96, s	29.9,[a] CH_3	2.12,[b] s	21.5,[c] CH_3
O<u>C</u>O-15		169.8, C	-	169.5, C
OCO<u>Me</u>-15	2.10, s	20.8,[a] CH_3	1.96,[b] s	20.7,[c] CH_3

[a-c] Interchangeable signals

(1a*S*,3a*S*,4*R*,4a*R*,5*S*,6*S*,7a*R*,9*R*,9a*R*,9b*S*)-1,1,6,9-tetramethyl-8-oxododecahydro-3a,9-methanocyclopropa[3,4]benzo[1,2-f]azulene-4,5,7a(4*H*)-triyl triacetate (cycloeuphoboetirane A) (2). Purified by analytical HPLC (*n*-hexane:EtOAc 82:18, t_R = 15 min, flow 1.0 mL/min), 0.3 mg, 0.1% yield.

Colourless oil; $[\alpha]_D^{21}$= -2.9 (c 0.04, $CHCl_3$); ^1H and ^{13}C NMR data, see Table S1; IR (film) υ_{max} 2923, 1739, 1459, 1374, 1243, 1023 cm^{-1}; ECD (MeOH) λ (Δε) 234 (-0.58), 273 (0.05), 3.32 (0.22), 365 (0.10) nm; HRMS (ESI) m/z: $[M+Na]^+$ Calcd for $C_{26}H_{36}O_7Na$ 483.2355; found 483.2353.

(2*S*,3*S*,4*R*,5*R*,9*S*,11*R*,12*R*,15*R*)-3,5,15-triacetoxy-12-hydroxylathyra-6(17),13(20)-dien-14-one (3). Purified by semi-preparative HPLC (*n*-hexane:EtOAc 70:30, t_R = 11 min, flow 3.0 mL/min), 1.34 mg, 0.4% yield. Colourless oil; $[\alpha]_D^{21}$= -11.0 (c 0.02, CH_3OH); ^1H and ^{13}C NMR data, see Table S1; IR (film) υ_{max} 3457, 2918, 2850, 1741, 1676, 1371, 1238, 1058, 944 cm^{-1}; ECD (MeOH) λ (Δε) 210 (-1.62), 230 (3.89), 261 (-0.75) nm; HRMS (ESI) m/z: $[M+H]^+$ Calcd for $C_{26}H_{37}O_8$ 477.2488; found 477.2505.

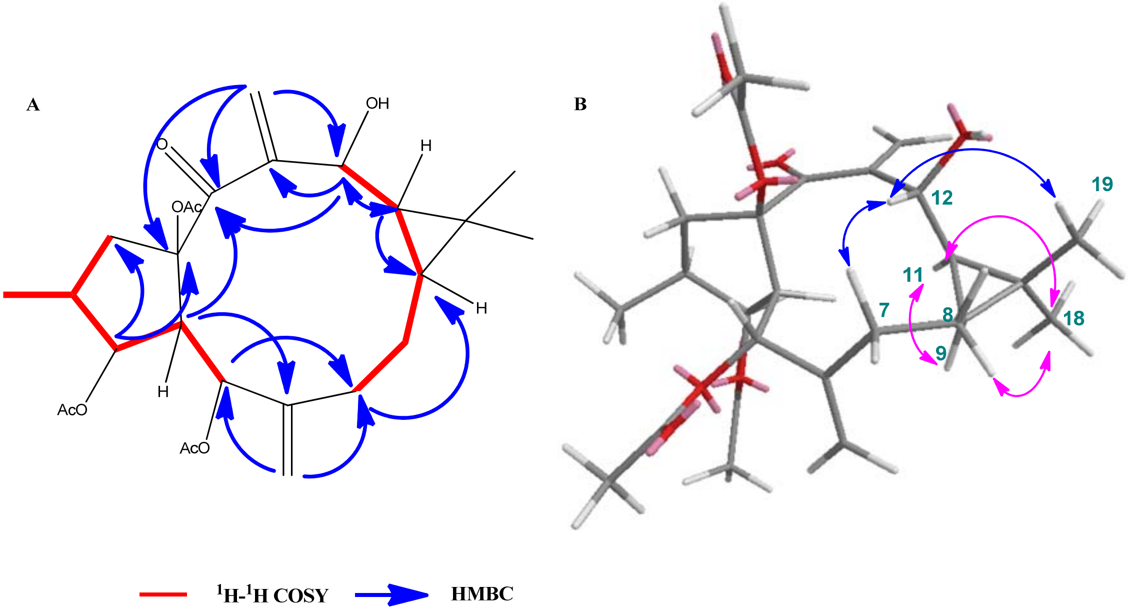

Figure S1. Key 2D NMR correlation of **3**. **A)** COSY and HMBC correlations. **B)** Selected 1D and 2D NOESY correlations for compound **3**. *β*-Face correlations in blue, *α*-Face correlations in purple.

3.3- Resting cell culture

The strain ST1-UCA was cultured in Petri dishes on Potato Dextrose Agar (PDA, Condalab, Madrid, Spain) at 25°C under white light (daylight lamp) for 14 days. Six mycelium plugs (1 cm diameter) from this culture were transferred to was cultured in six 500 mL-Erlenmeyer flaks, each containing 200 mL of sterilized PDB medium, at 200 rpm and 25 °C under white light for 3 days. Then, the mycelium was transferred to the same number of flasks, each containing 200 mL of Czapek-Dox medium without glucose (1 g of yeast extract, 5 g of K_2HPO_4, 2 g of $NaNO_3$, 0.5 g of $MgSO_4 \cdot 7H_2O$, and 0.01 g of $FeSO_4 \cdot 7H_2O$ per litre of distilled water) and euphoboetirane A (**1**) dissolved in 300 µL of DMSO (85 ppm). The flasks were then incubated for an additional period of one to five days under the aforementioned conditions. The broths were filtered and extracted with ethyl acetate (3x). The organic extracts were dried over anhydrous Na_2SO_4 and the solvents were removed under reduced pressure. Then, the crude was purified by analytical HPLC (*n*-hexane:EtOAc 82:18, flow 1.0 mL/min) (Table S2 and Figures S1-4). The best result for cycloeuphoboetirane A (**2**) (0.6% yield) was achieved when the resting cell culture was incubated with the substrate for three days. Moreover, compound **4** was obtained in 38% yield after this incubation period.

(12*Z*)-Euphoboetirane A (4). Purified by analytical HPLC (*n*-hexane:EtOAc 82:18, t_R = 22 min, flow 1.0 mL/min). The spectroscopic data are consistent with those described in literature.[1]

Table S2. HPLC analysis of biotransformation of compound **1** using resting cell culture.

entry	day	yield of 2	yield of 4
1	1	0.2%	-
2	3	0.6%	0.9%
3	5	0.4%	38%

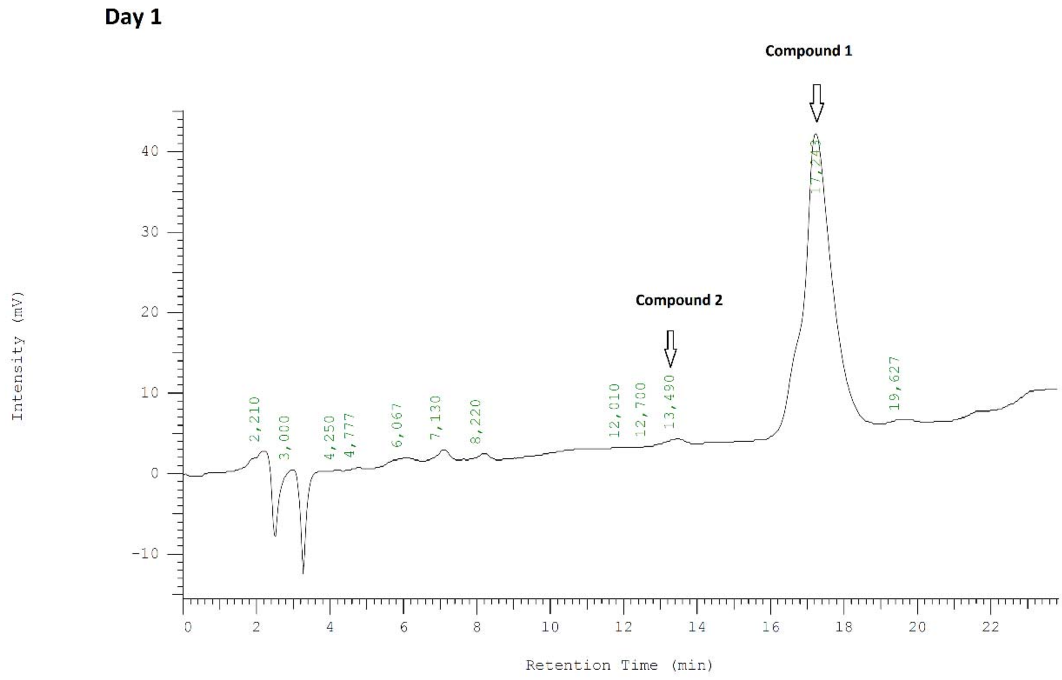

Figure S2. HPLC analysis of biotransformation of **1** using resting cell culture (day 1).

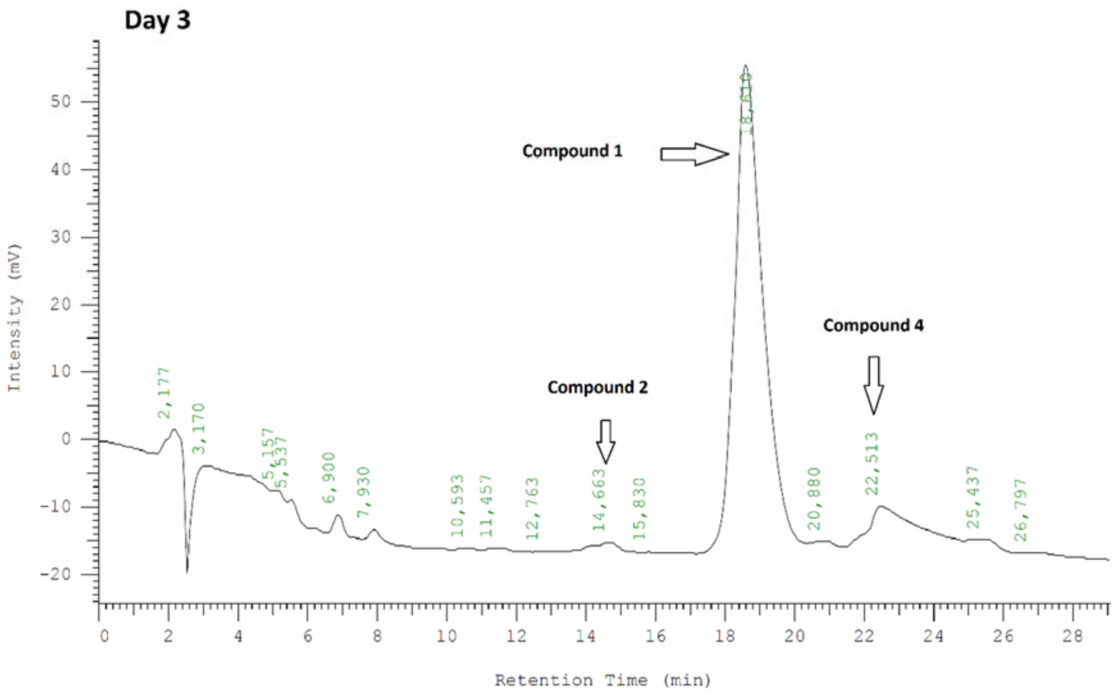

Figure S3. HPLC analysis of biotransformation of **1** using resting cell culture (day 3).

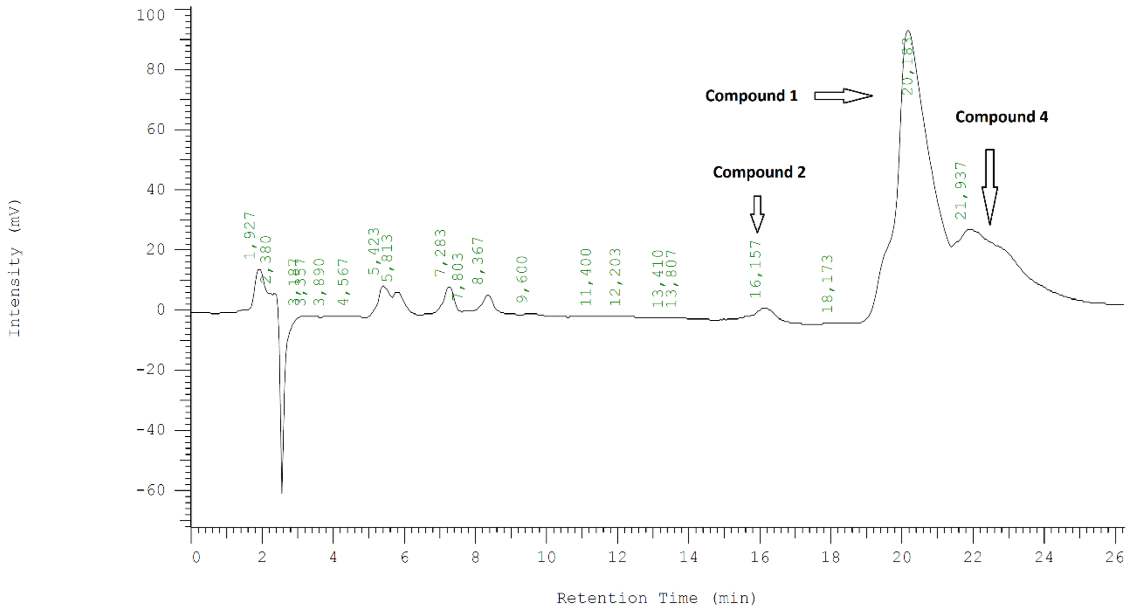

Figure S4. HPLC analysis of biotransformation of **1** using resting cell culture (day 5).

4. General procedure for the photocatalyzed formal [2+2] intramolecular cycloaddition

Euphoboetirane A (**1**) (0.04 mmol, 1.0 eq.) was added to an oven-dried 10 mL glass tube equipped with a magnetic stir bar. The tube was set under an argon atmosphere. The respective photocatalyst ($Ir(ppy)_3$ or benzophenone) was added, followed by the addition of the solvent via syringe (2 mL). The syringe was previously degassed by argon sparging for 5 minutes. The reaction mixture was irradiated under blue LED (18W, 445 nm) or bluish-white LED (18W, 365 nm) at room temperature overnight. Subsequently, ethyl acetate was added (6 mL) and the organic layer washed with water (5 mL), brine (5 mL) and dried over anhydrous Na_2SO_4. Solvent was removed under reduced pressure to give a crude, which was purified by silica gel column chromatography using as eluent *n*-hexane:EtOAc (95:5) to afford compounds **5-8** (See Table S3).

(9*R*,12*Z*)-Euphoboetirane A (5). Purified by semi-preparative HPLC (*n*-hexane:EtOAc 85:15, t_R = 38 min, flow 3.0 mL/min). Colourless oil; $[\alpha]_D^{21}$= -42.6 (c 0.24, $CHCl_3$); ^1H and ^{13}C NMR spectra, see Figures S28-29; IR (film) υ_{max} 2937, 2877, 1746, 1709, 1455, 1372, 1251, 1149, 1021, 756 cm^{-1}; HRMS (ESI) m/z: $[M+Na]^+$ Calcd for $C_{26}H_{36}O_7Na$ 483.2359; found 483.2359.

(2*S*,3*S*,4*R*,5*R*,9*S*,13*S*,15*R*)-3,5,15-Tri-*O*-acetyl-lathyra-6(17),10(19),11*E*-dien-14-one (6). Purified by analytical HPLC (*n*-hexane:EtOAc 82:18, t_R = 20 min, flow 1.0 mL/min). Colourless oil; $[\alpha]_D^{21}$= +21.6 (c 0.11, $CHCl_3$); ^1H and ^{13}C NMR data, see Table S4 and S5; IR (film) υ_{max} 2918, 2851, 1742, 1709, 1538, 1455, 1370, 1240, 1138, 1069, 1021 cm^{-1}. HRMS (ESI) m/z: $[M+Na]^+$ Calcd for $C_{26}H_{36}O_7Na$ 483.2359; found 483.2364.

(2*S*,3*S*,4*R*,5*R*,9*R*,11*R*,15*R*)-3,5,15-Tri-*O*-acetyl-17-(dichloromethyl)lathyra-6(7)*Z*,12*Z*-dien-14-one (7). Colourless oil; $[\alpha]_D^{21}$= +84.2 (c 0.11, $CHCl_3$); ^1H and ^{13}C NMR data, see Table S4 and S5; IR (film) υ_{max} 3020, 2919, 2850, 1737, 1709, 1462, 1372, 1253, 1216, 1020, 758, 668 cm^{-1}; ECD (MeOH) λ (Δε) 210 (-0.06), 236 (1.06), 250 (0.03), 271 (0.09), 321 (0.06) nm; HRMS (ESI) m/z: $[M+Na]^+$ Calcd for $C_{27}H_{36}O_7Cl_2Na$ 565.1736; found 565.1757.

(2*S*,3*S*,4*R*,5*R*,9*R*,13*S*,15*R*)-3,5,15-Tri-*O*-acetyl-17-(dichloromethyl)-10,11-secolathyra-6(7)*Z*,10(19),11*E*-trien-14-one (8). Purified by analytical HPLC (*n*-hexane:EtOAc 82:18, t_R = 18 min, flow 1.0 mL/min). Colourless oil; $[\alpha]_D^{21}$= -16.4 (c 0.28, $CHCl_3$); ^1H and ^{13}C NMR data, see Table S4 and S5; IR (film) υ_{max} 2921, 2851, 1739, 1579, 1541, 1455, 1373, 1238, 1154, 1023, 756 cm^{-1}; HRMS (ESI) m/z: $[M+Na]^+$ Calcd for $C_{27}H_{36}O_7Cl_2Na$ 565.1736; found 565.1750.

Table S3. Optimization of the reaction conditions for the photocatalyzed formal [2+2] intramolecular cycloaddition of 1.[a]

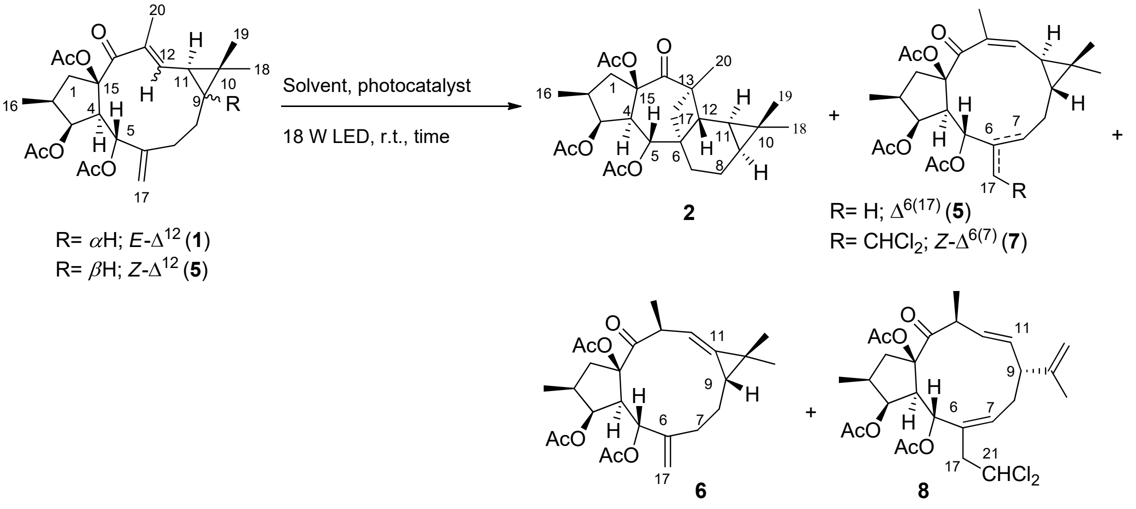

Entry	lathyrane	solvent	Photocatalyst (mol%)	LED	Time (h)	2/5/6/7/8[b]
1	**1**	DMF	Ir(ppy)3 (2.5)	365 nm	24	0/82/0/0/0
2	**1**	DMF	Ir(ppy)3 (2.5)	365 nm	48	0/51/0/0/0
3	**1**	DMF	Ir(ppy)3 (2.5)	365 nm	72	0/59/0/0/0
4	**1**	DMF	Ir(ppy)3 (2.5)	445 nm	24	0/82/0/0/0
5	**1**	DMF	Ir(ppy)3 (30)	365 nm	24	8/47/0/0/0
6	**1**	DMF	BP (30)	365 nm	24	0/72/0/0/0
7	**1**	DMF	BP (30)	365 nm	48	14/20/17/0/0
8	**1**	DMF	BP (60)	365 nm	24	35/41/0/0/0
9	**1**	DMF	BP (60)	365 nm	48	55/0/0/0/0
10	**1**	DCM	BP (30)	365 nm	24	0/46/0/32/0
11	**1**	DCM	BP (60)	365 nm	24	0/0/0/47/28
12	**1**	DCM	BP (60)	365 nm	48	0/0/0/16/34
13	**5**	DMF	BP (60)	365 nm	24	16/58/0/0/0
14	**5**	DMF	BP (60)	365 nm	48	24/51/0/0/0

[a] Reactions were conducted with **1** (0.04 mmol), solvent (2 mL), photocatalyst and irradiation LED light at room temperature under an argon atmosphere. [b] Yields of isolated compounds.

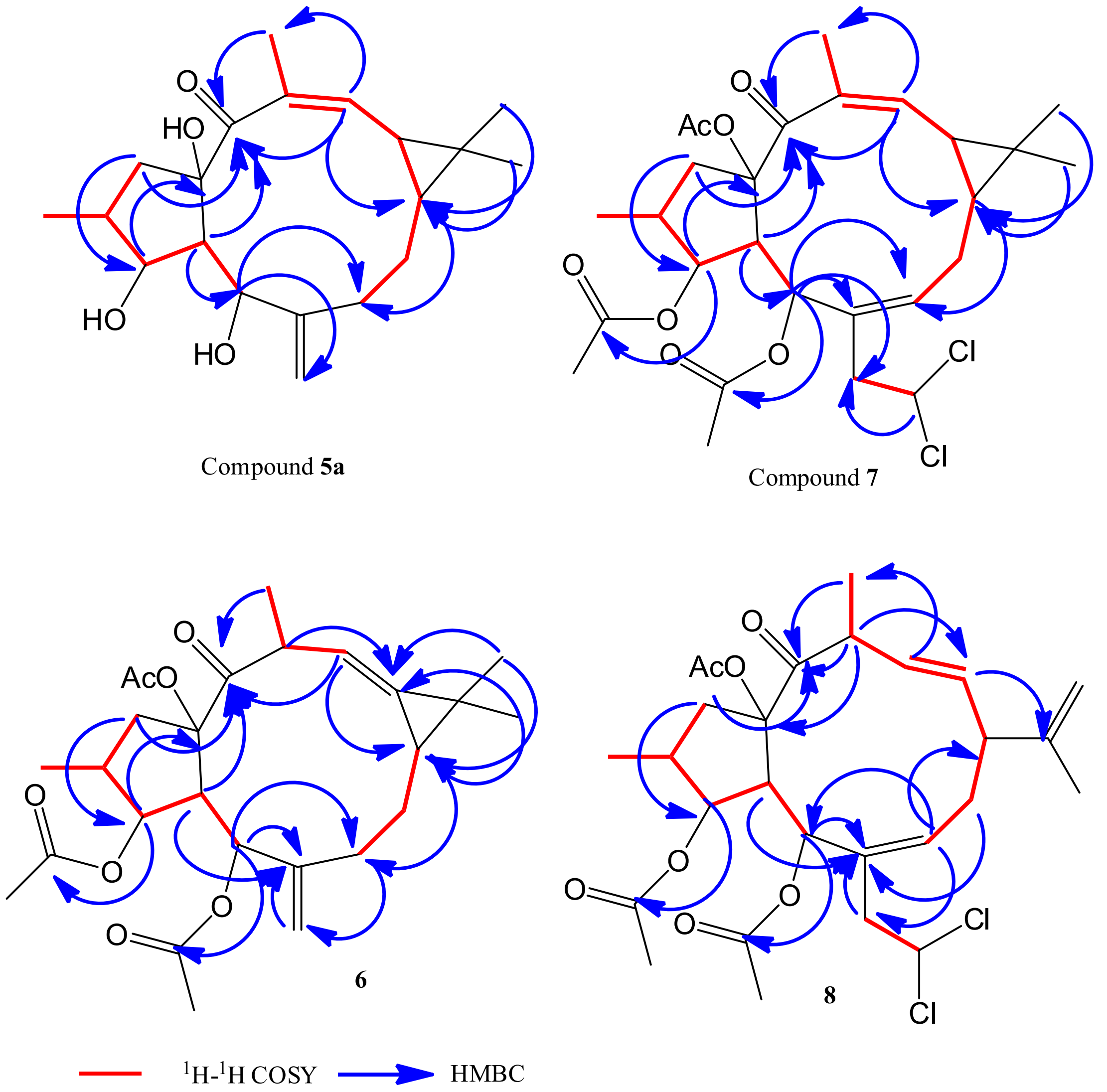

Figure S5. Key ^{1}H-^{1}H COSY and HMBC correlations for compounds **5a** and **6-8**.

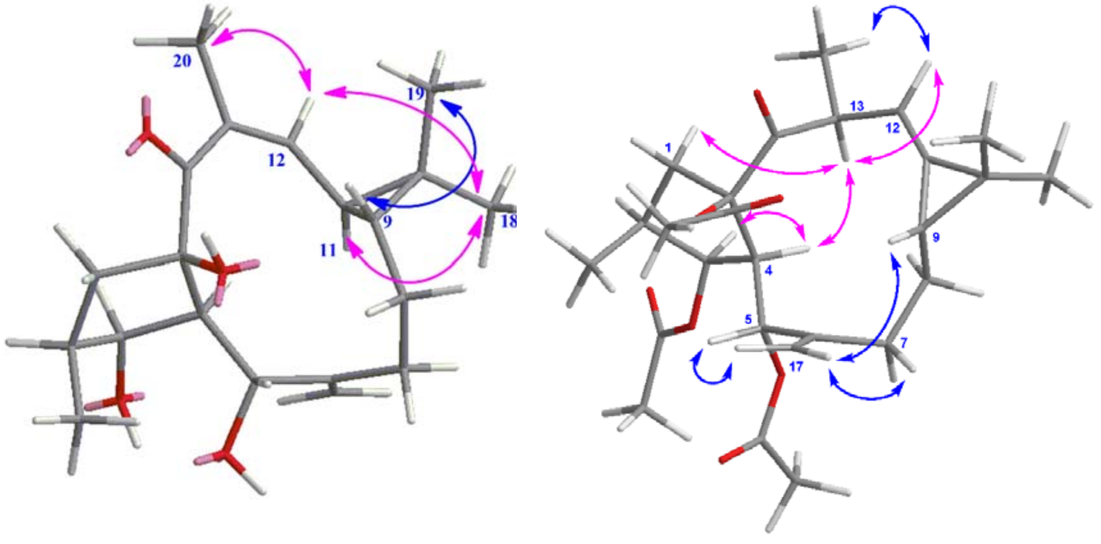

Figure S6. Selected 1D and 2D NOESY correlations for compounds **5a** (left) and **6** (right). *β*-Face correlations in blue, *α*-Face correlations in purple.

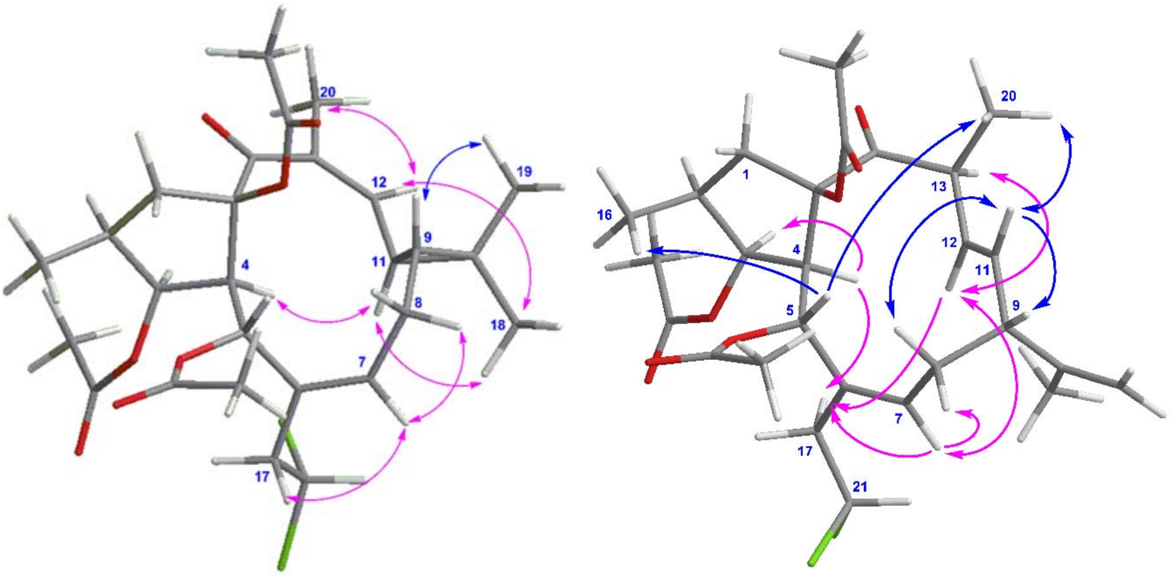

Figure S7. Selected 1D and 2D NOESY correlations for compounds **7** (left) and **8** (right). β-Face correlations in blue, α-Face correlations in purple.

Table S4. ^{1}H NMR Spectroscopic data for compounds **5a** (500 MHz, CD_3OD), **7** (500 MHz, $CDCl_3$), **6**, and **8** (700 MHz, $CDCl_3$) (δ_H in ppm, J in Hz).

Position	**5a**	**6**	**7**	**8**
1α	2.14, dd (12.5, 6.5)	2.99, dd (15.6, 7.8)	2.99, dd (15.0, 7.7)	2.33-2.29, m (overlaid)
1β	1.75, t (12.5)	2.02, dd (15.6, 12.8)	1.83, dd (15.0, 13.1)	
2	1.89-1.81, m	2.27-2.21, m	2.15-2.08, m (overlaid)	2.19-2.16, m
3	4.18, t (3.6)	5.53, t (3.8)	5.51, t (4.0)	5.50, t (3.6)
4	2.95, dd (10.1, 3.6)	2.76, dd (11.0, 3.8)	3.64, dd, (10.9, 4.0)	3.38, dd, (11.3, 3.6)
5	4.56, d (10.1)	5.82, d (11.0)	5.38, dd (10.9, 1.1)	5.73, d (11.3)
7α	1.96-1.91, m	1.93-1.88, m	5.62 (dd, 11.5, 7.2)	5.69 (dd, 12.0, 4.5)
7β	2.38-2.27, m	2.00-1.96, m		
8α	2.10-1.98, m	1.42-1.37, m	2.15-2.08, m (overlaid)	2.23-2.18, m
8β	1.26-1.15, m	2.03-2.01, m	2.33, dd (13.5, 7.2)	2.33-2.29, m (overlaid)
9	0.71, dt (10.9, 5.7)	1.27-1.23, m	0.28, dd (8.7, 5.6)	2.64-2.59, m (overlaid)
11	1.16-1.11, m	-	0.74, brs	5.05, dd (15.7, 9.9)
12	5.36, dd (7.2, 1.7)	5.55, d (2.4)	5.60 s	5.33, dd (15.7, 8.4)
13	-	3.48-3.44, m	-	3.72-3.68, m
16	1.05, d (6.7)	0.98, d (6.6)	0.95, d (7.0)	0.88, d (6.7)
17[a]	5.23, brs	5.36, brs	3.36 (dd, 14.8, 3.3)	2.63 (dd, 15.0, 4.2)
17b	4.97, d (2.0)	5.08, d (1.5)	2.90 (dd, 14.8, 10.0)	2.77 (dd, 15.0, 10.0)
18	1.10, s	1.10, s	1.04, s	1.74, d (1.1)
19a	1.02, s	0.99, s	0.98, s	4.80, brs
19b				4.76, brs
20	1.94, dd (1.6, 1.0)	1.36, d (7.0)	2.00, dd (2.3, 1.6)	1.15, d (6.7)
21	-	-	6.19, dd (10.0, 3.3)	5.88, dd (10.0, 4.2)
OCOMe-3	-	2.06, s	2.08, s	2.01, s
OCOMe-5	-	1.96, s	1.96, s	1.98, s[a]
OCOMe-15	-	2.15, s	2.15, s	2.25, s[a]

[a] Interchangeable signal

Table S5. ^{13}C NMR Spectroscopic data for compounds **5a** (125 MHz, CD_3OD), **7** (125 MHz, $CDCl_3$), **6**, and **8** (175 MHz, $CDCl_3$) (δ_C in ppm).

Position	5a, type	6, type	7, type	8, type
1	49.6, CH_2	46.0, CH_2	45.4, CH_2	45.3, CH_2
2	38.7, CH	38.4, CH	38.2, CH	37.1, CH
3	77.6, CH	77.3, CH	76.1 CH	77.8, CH
4	55.9, CH	49.6, CH	52.3, CH	54.0, CH
5	75.4, CH	73.7, CH	68.6, CH	74.8, CH
6	151.1, C	140.0, C	131.1, C	129.4, C
7	29.1, CH_2	30.1, CH_2	136.9, CH	138.7, CH
8	24.2, CH_2	22.4, CH_2	27.3, CH_2	31.9, CH_2
9	34.9, CH	26.4, CH	31.8, CH	51.1, CH
10	22.2, C	16.9, C	20.8 C	146.1, C
11	26.6, CH	139.5, CH	33.5, CH	132.8, CH
12	132.7, CH	116.5, CH	125.4, CH	128.0, CH
13	140.6, C	40.1, CH	142.7, C	44.4, CH
14	216.4, C	202.1, C	202.5, C	208.5, C
15	87.3, C	90.7 C	90.6 C	93.9 C
16	13.7, CH_3	13.7, CH_3	13.4, CH_3	13.5, CH_3
17	114.0, CH_2	117.2, CH_2	52.6, CH_2	40.9, CH_2
18	22.7, CH_3	18.7, CH_3	21.4,[a] CH_3	21.6, CH_3
19	21.5, CH_3	26.6, CH_3	21.1,[a] CH_3	110.2, CH_2
20	20.5, CH_3	22.2, CH_3	23.3, CH_3	14.8, CH_3
21	-	-	72.1,CH	72.4,CH
O<u>C</u>O-3	-	170.6, C	170.8, C	170.3, C
OCO<u>C</u>H$_3$-3	-	21.0, CH_3	20.8, CH_3	20.7, CH_3
O<u>C</u>O-5	-	169.3, C	169.9, C	169.5, C[b]
OCO<u>C</u>H$_3$-5	-	20.8, CH_3	21.0,[a] CH_3	21.4, CH_3[c]
O<u>C</u>O-15	-	169.6, C	169.0, C	169.4, C[b]
OCO<u>C</u>H$_3$-15	-	21.4, CH_3	21.1,[a] CH_3	22.5, CH_3[c]

[a-c] Interchangeable signals

4.1 Hydrolysis of compound 5

A solution of 1M KOH in methanol (2 mL) was added to a stirred solution of **5** (10.0 mg, 0.02 mmol) in methanol (1 mL) and stirred for 16 hours at r.t. Solvent was evaporated under reduced pressure and water was added (5 mL). The aqueous layer was extracted with ethyl acetate (3 x 5 mL). The organic layer was dried over anhydrous Na_2SO_4 and solvent removed under reduced pressure to give a crude. This was purified by silica gel column chromatography to afford compound **5a** (6.9 mg, 95% yield).

(9*R*,12*Z*)-lathyrol (5a).[3] Colourless oil; $[\alpha]_D^{21}$= +40.3 (c 0.08, CH_3OH); ^1H and ^{13}C NMR data, see Table S4 and S5; IR (film) υ_{max} 3448, 2931, 1687, 1455, 1377, 1152, 810 cm^{-1}; ECD (MeOH) λ (Δε) 242 (-0.38), 275 (0.78), 302 (0.52), 329 (0.86) nm.

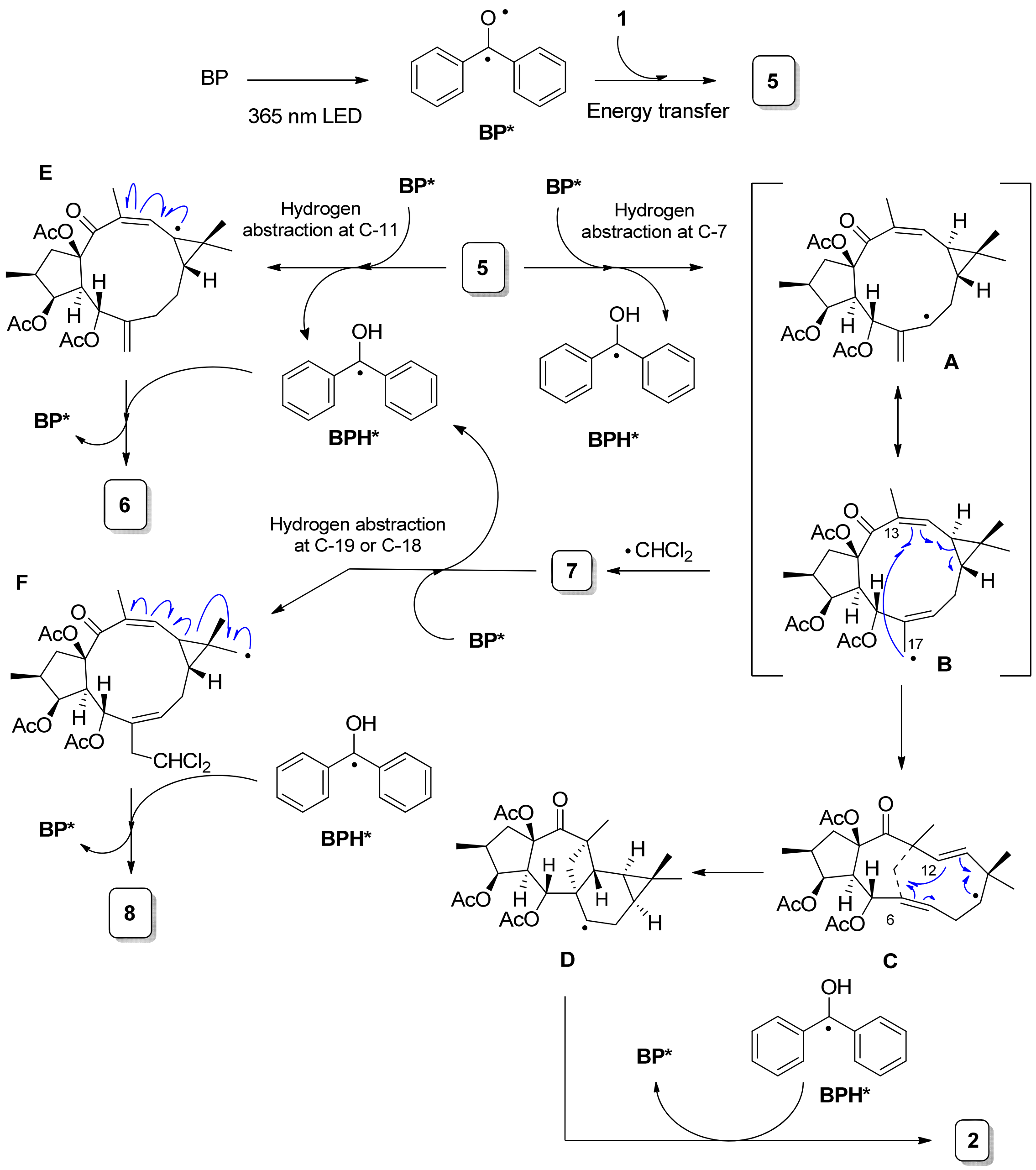

Scheme S1. Proposed mechanism for the formal [2+2] cycloaddition of **1** and its transformation into compounds **5**-**8**.

5. References

(1) Escobar-Montaño, F.; González-Rodríguez, V.E.; Macías-Sánchez, A. J.; Botubol-Ares, J. M.; Durán-Patrón, R.; Henández-Galán, R. Enhancing Structural Diversity of Lathyrane Derivatives through Biotransformation by the Marine-Derived actinomycete *Streptomyces puniceus* BC-5GB.11. *Int. J. Mol. Sci*. **2024**, *25*, 2289.

(2) Bolívar-Anillo, H. J.; Izquierdo-Bueno, I.; González-Rey, E.; González-Rodríguez, V. E.; Cantoral, J. M.; Collado, I. G.; Garrido, C. In Vitro Analysis of the Antagonistic Biological and Chemical Interactions between the Endophyte *Sordaria tomento-alba* and the Phytopathogen *Botrytis cinerea*. *Int. J. Mol. Sci.* **2024**, *25*, 1022.

(3) Wang, N.; Xu, J. B.; Li, X. H.; Zhou, X. L.; Gao, F. Ir-Catalyzed Biomimetic Photoisomerization of Cyclopropane in Lathyrane-Type Euphorbia Diterpenes. *Org. Lett.* **2022**, *24* (47), 8598–8602.

6. NMR, ECD and HRMS spectra

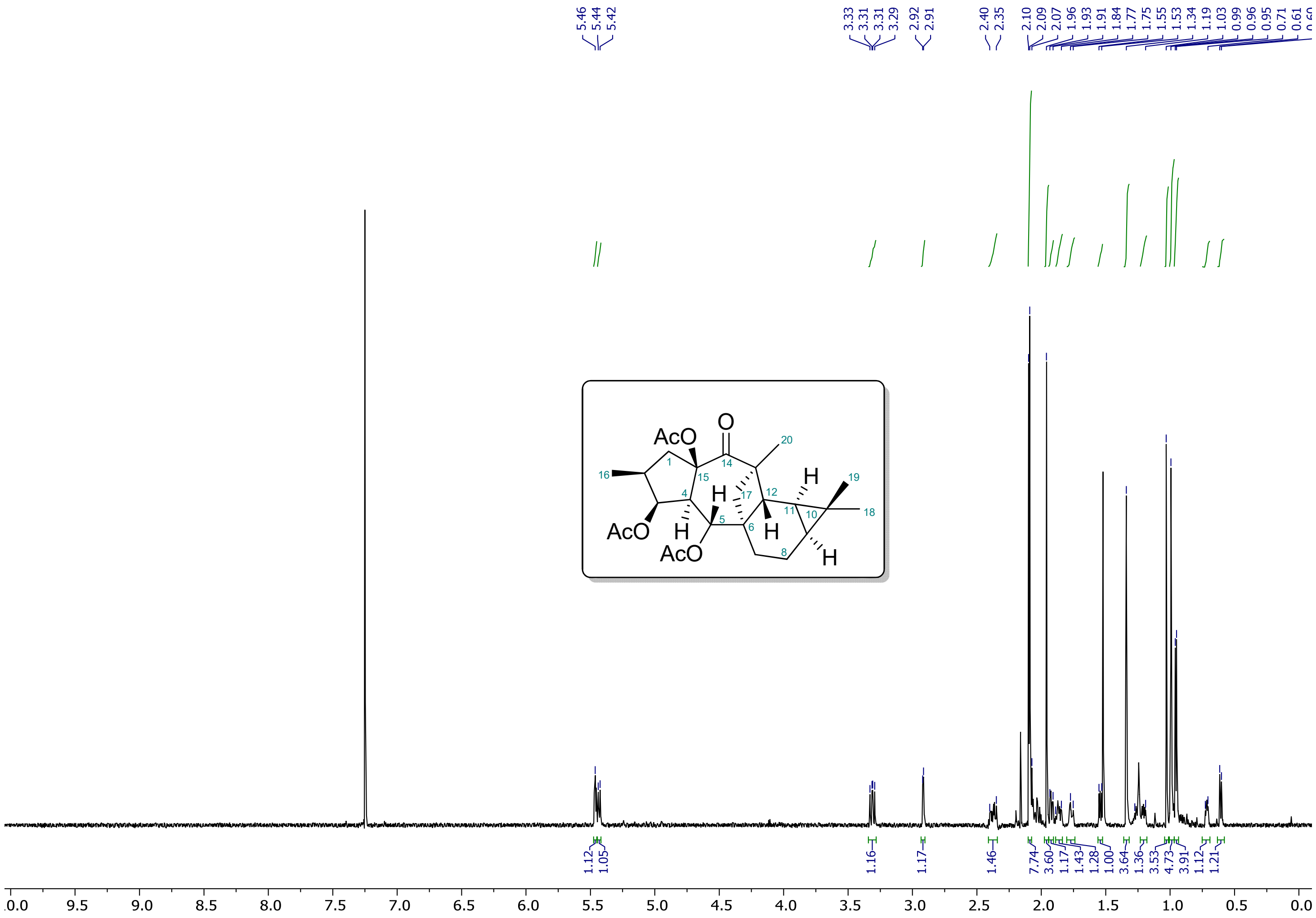

Figure S8. ^{1}H NMR spectrum (700 MHz) of compound **2** in $CDCl_3$.

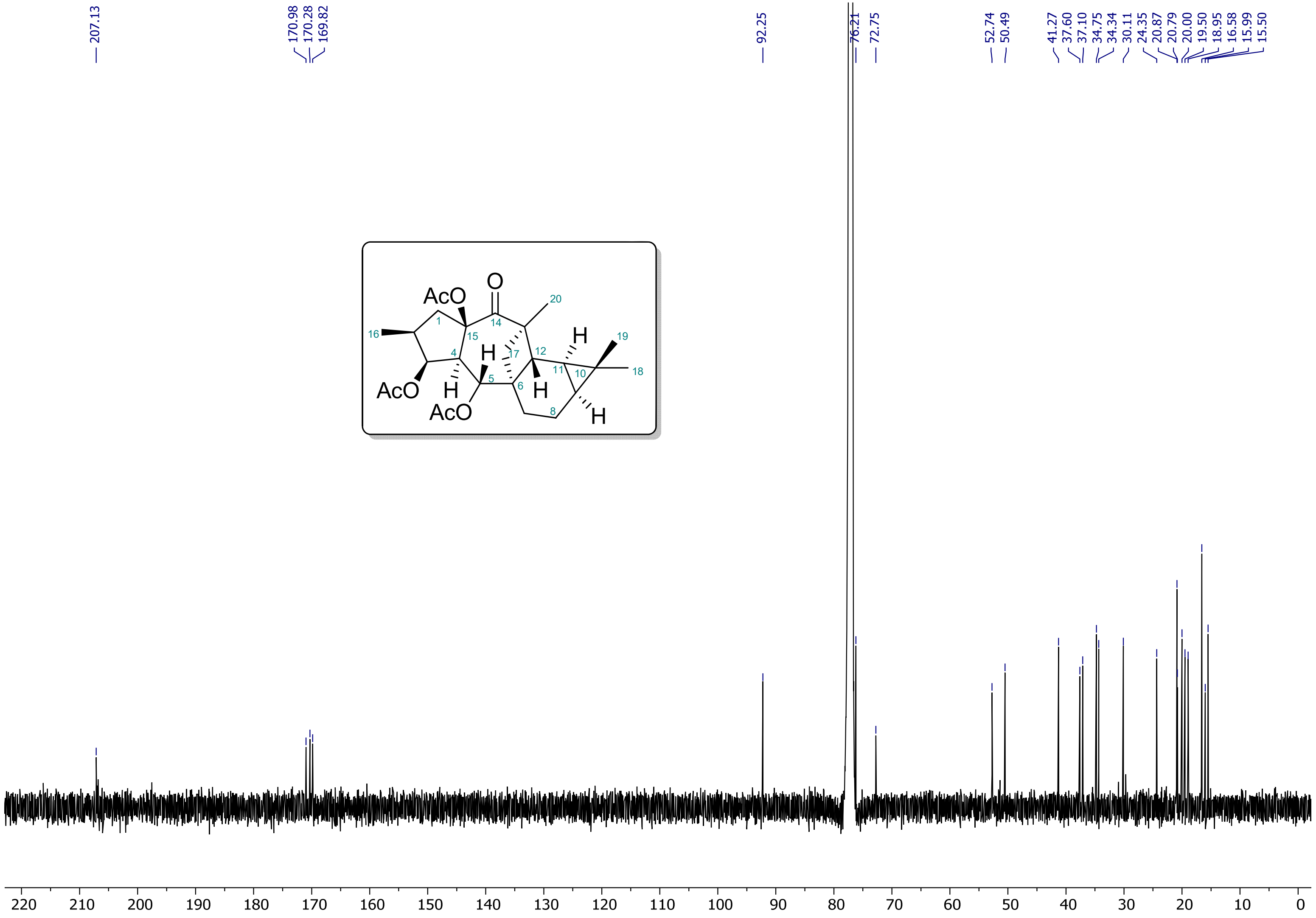

Figure S9. ^{13}C NMR spectrum (150 MHz) of compound **2** in $CDCl_3$.

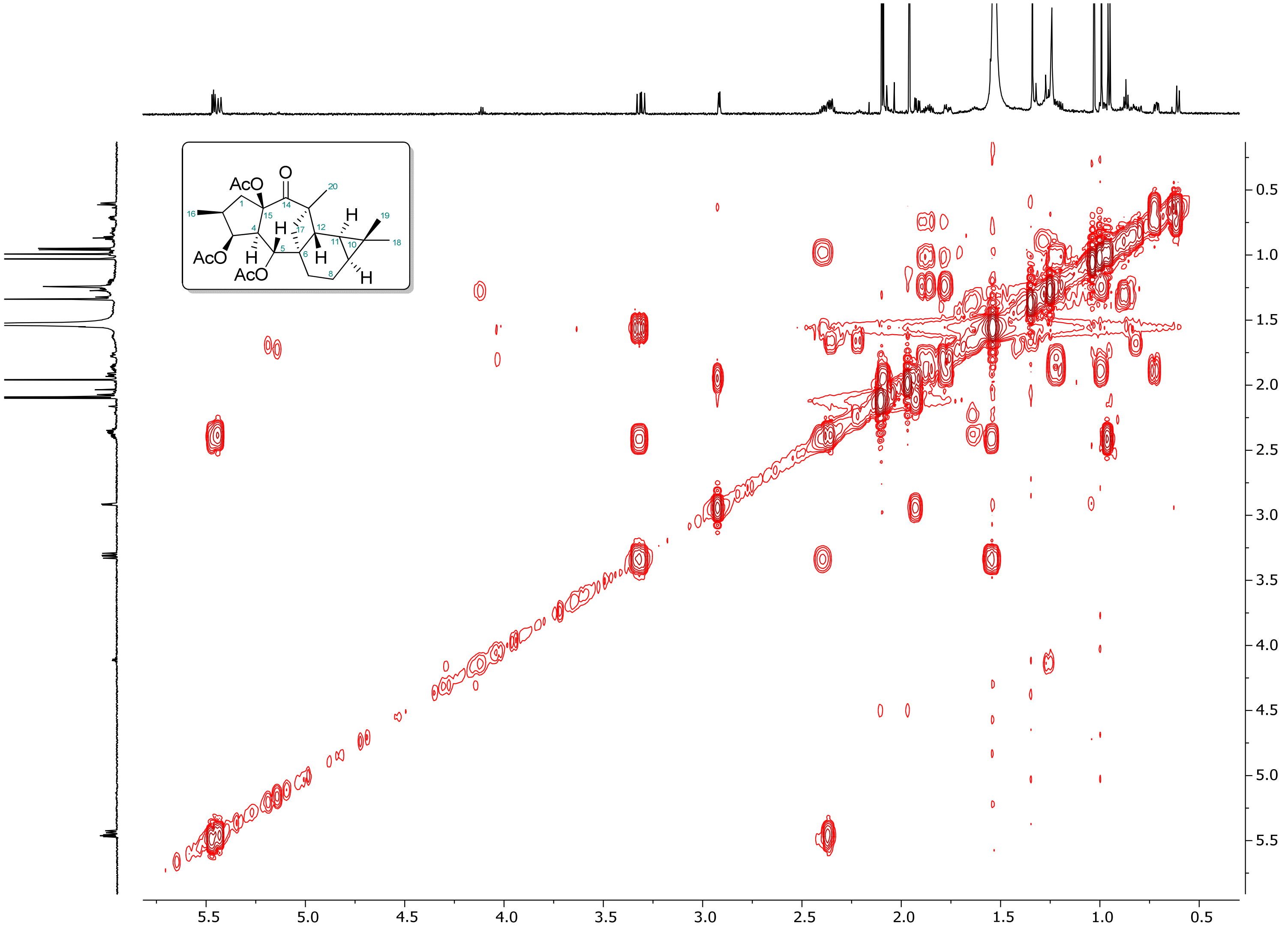

Figure S10. gCOSY spectrum of compound **2**.

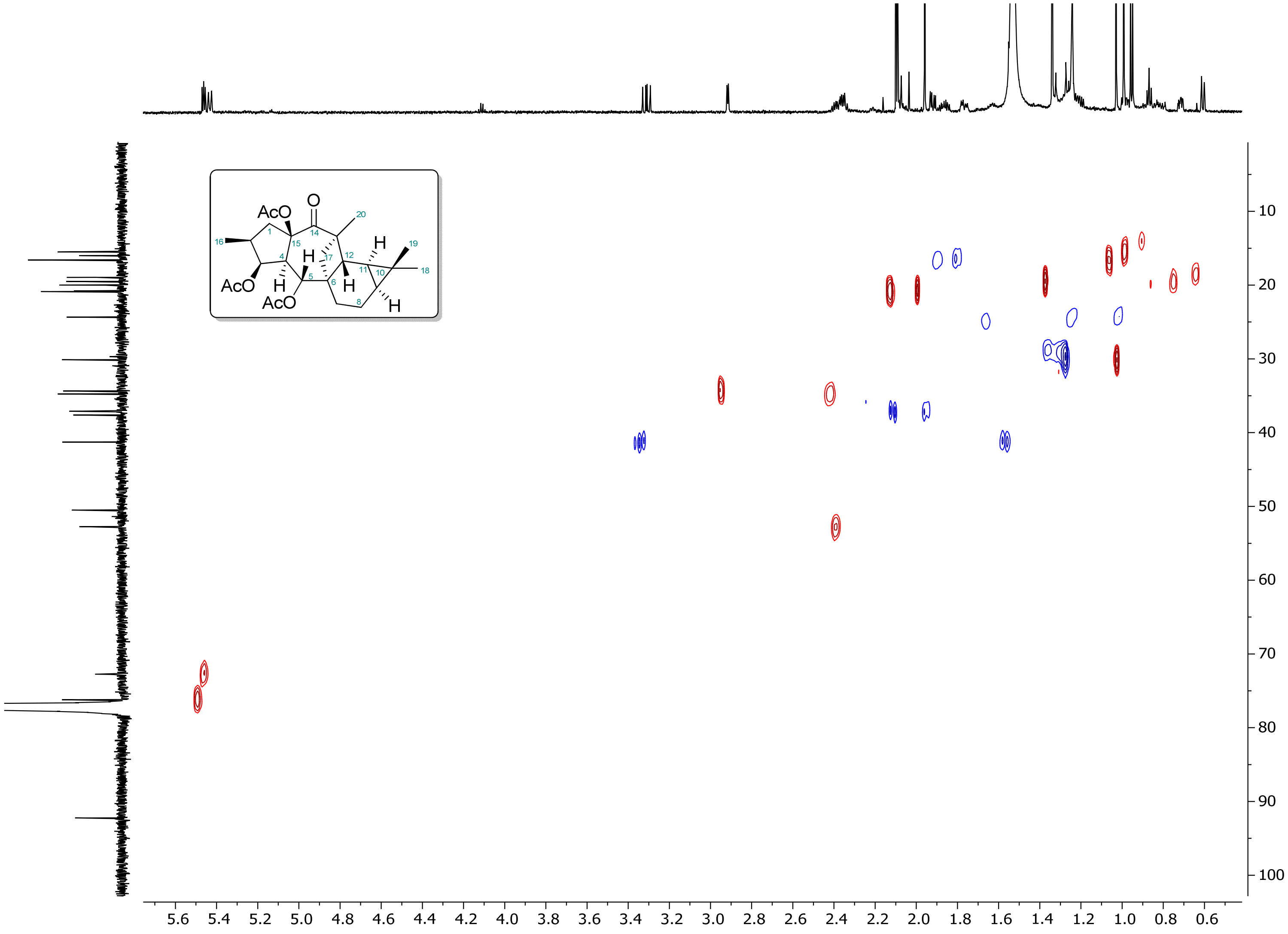

Figure S11. gHSQC spectrum of compound **2**.

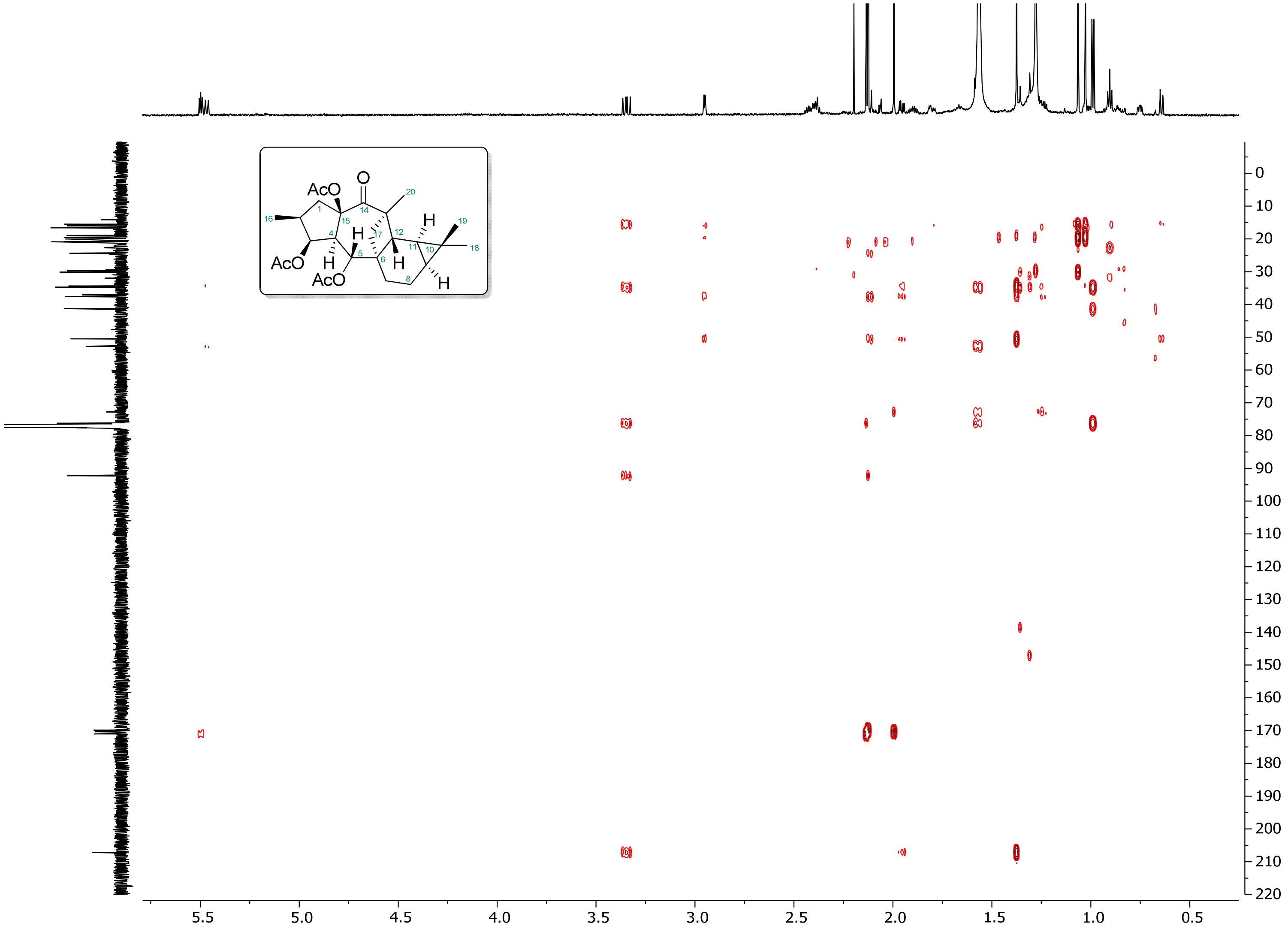

Figure S12. gHMBC (3 Hz) spectrum of compound **2**.

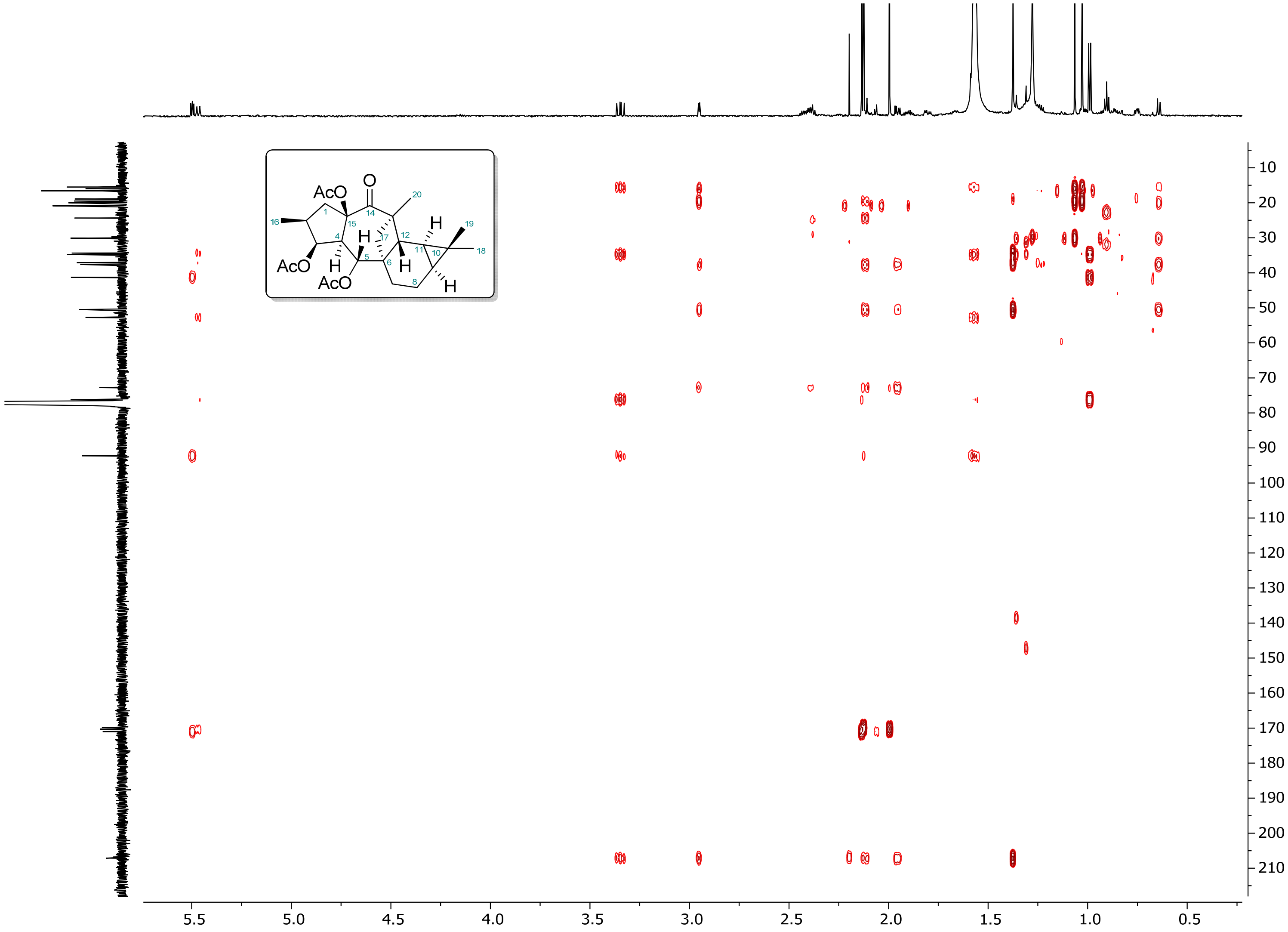

Figure S13. gHMBC (5 Hz) spectrum of compound **2**.

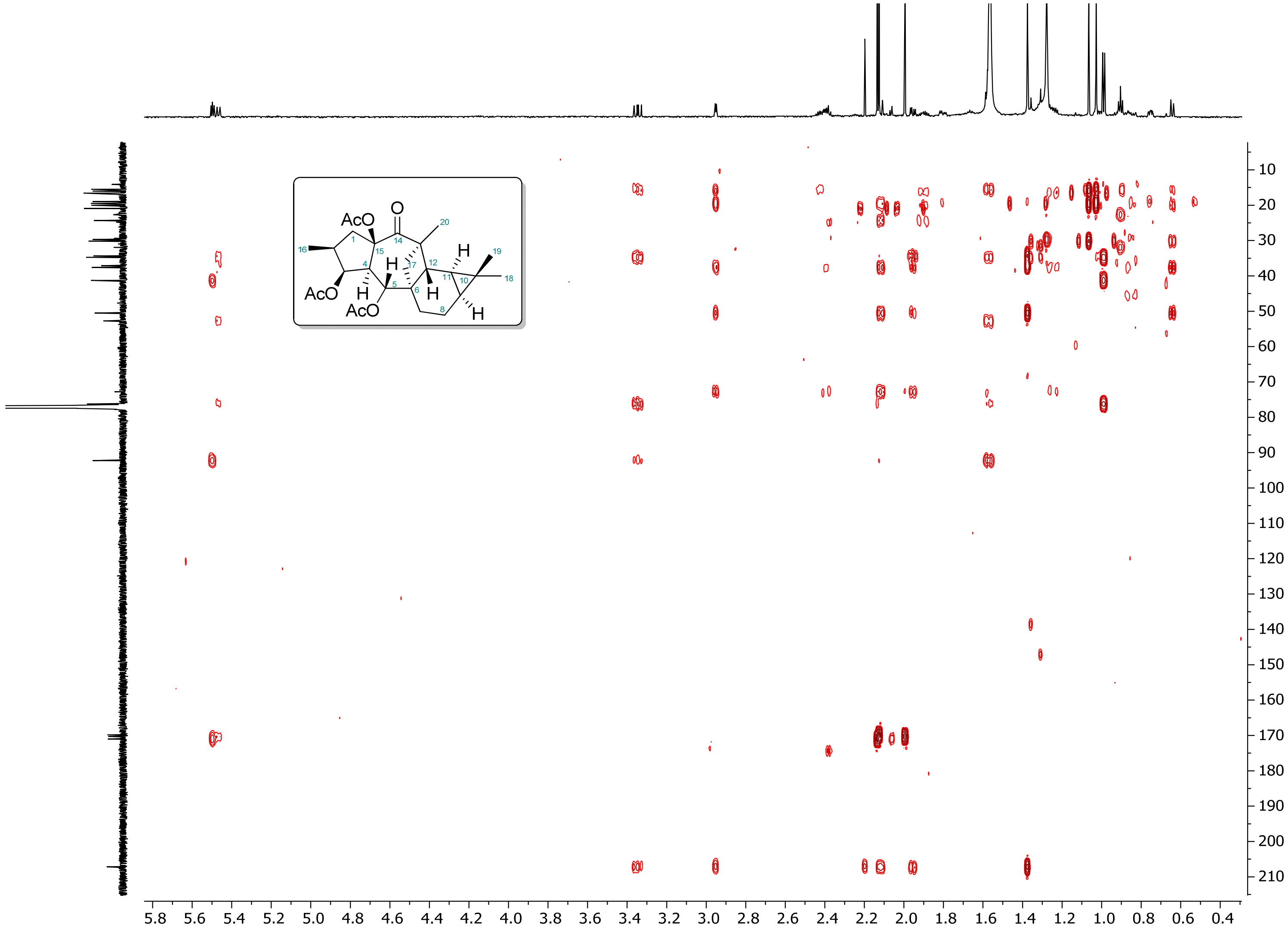

Figure S14. gHMBC (10 Hz) spectrum of compound **2**.

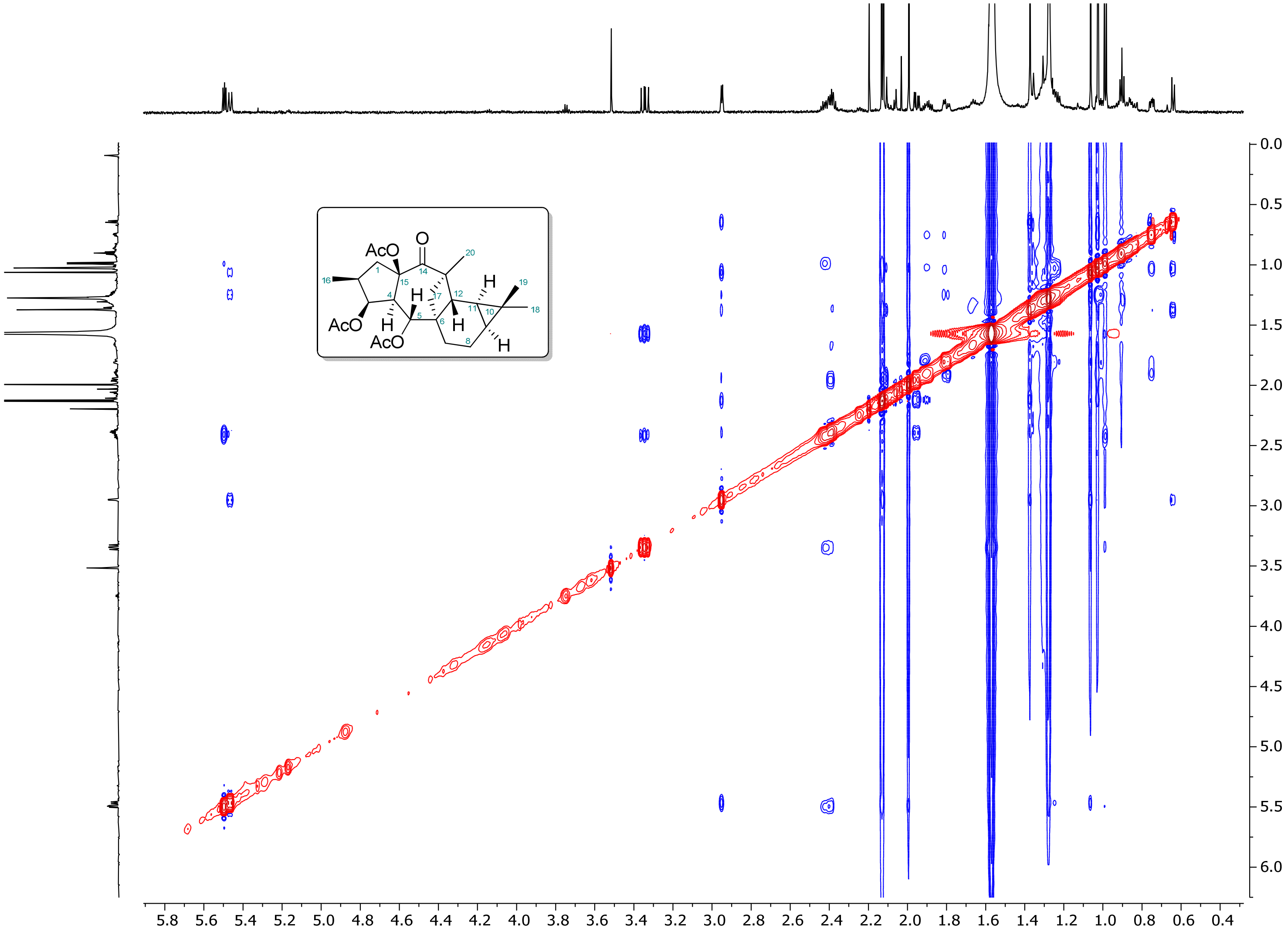

Figure S15. 2D NOESY spectrum of compound **2**.

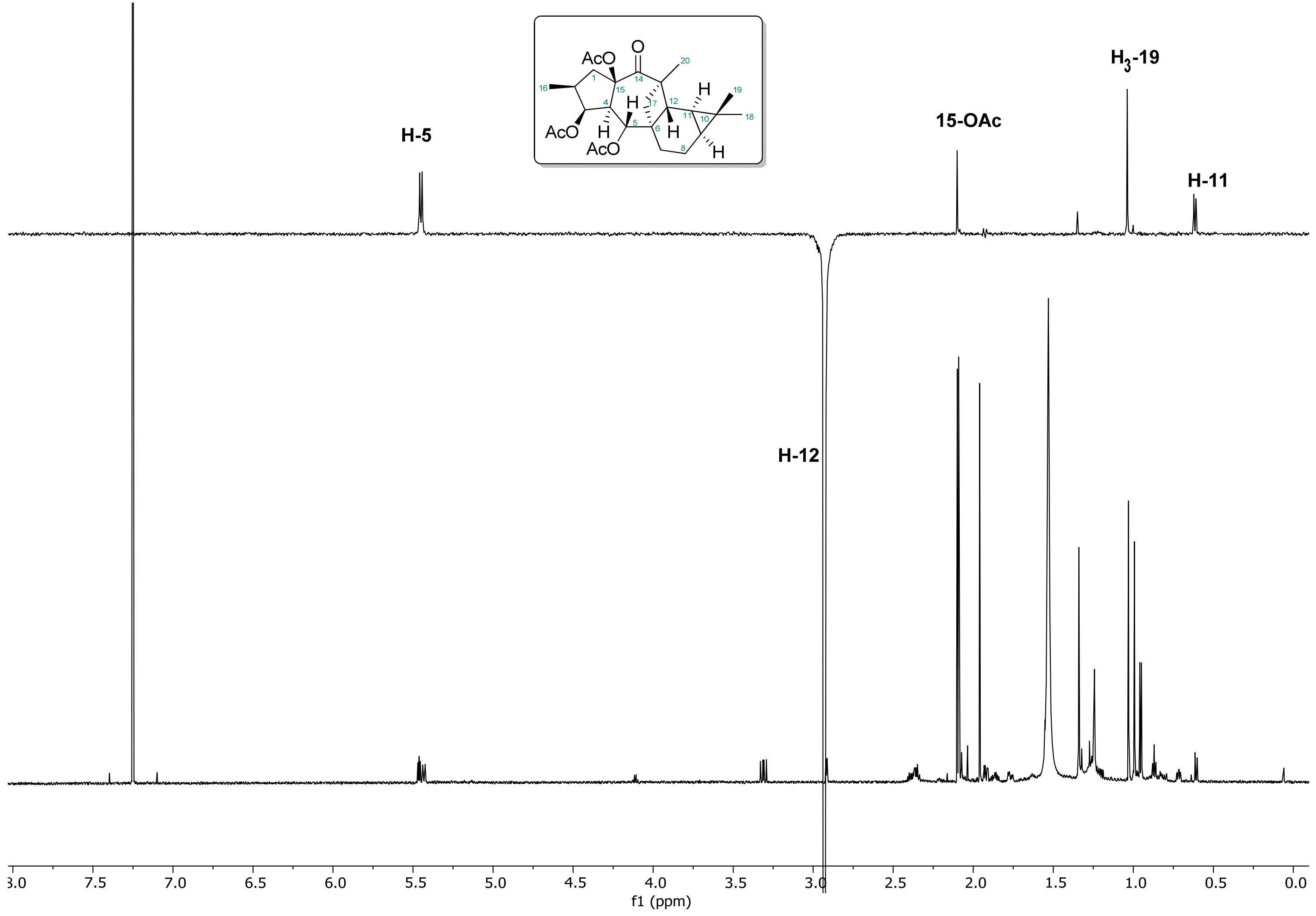

Figure S16a. 1D NOESY spectrum of compound **2**.

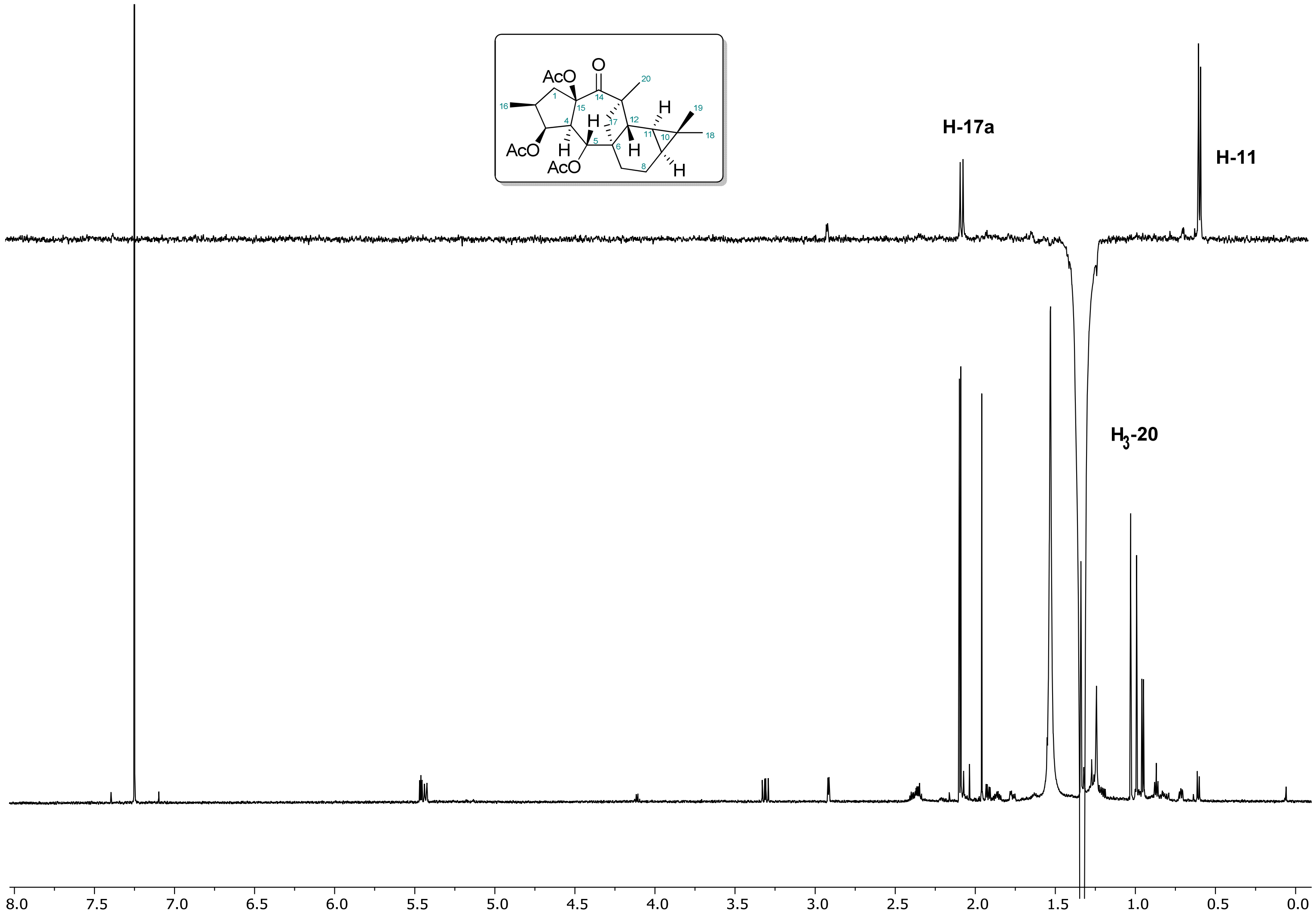

Figure S16b. 1D NOESY spectrum of compound **2**.

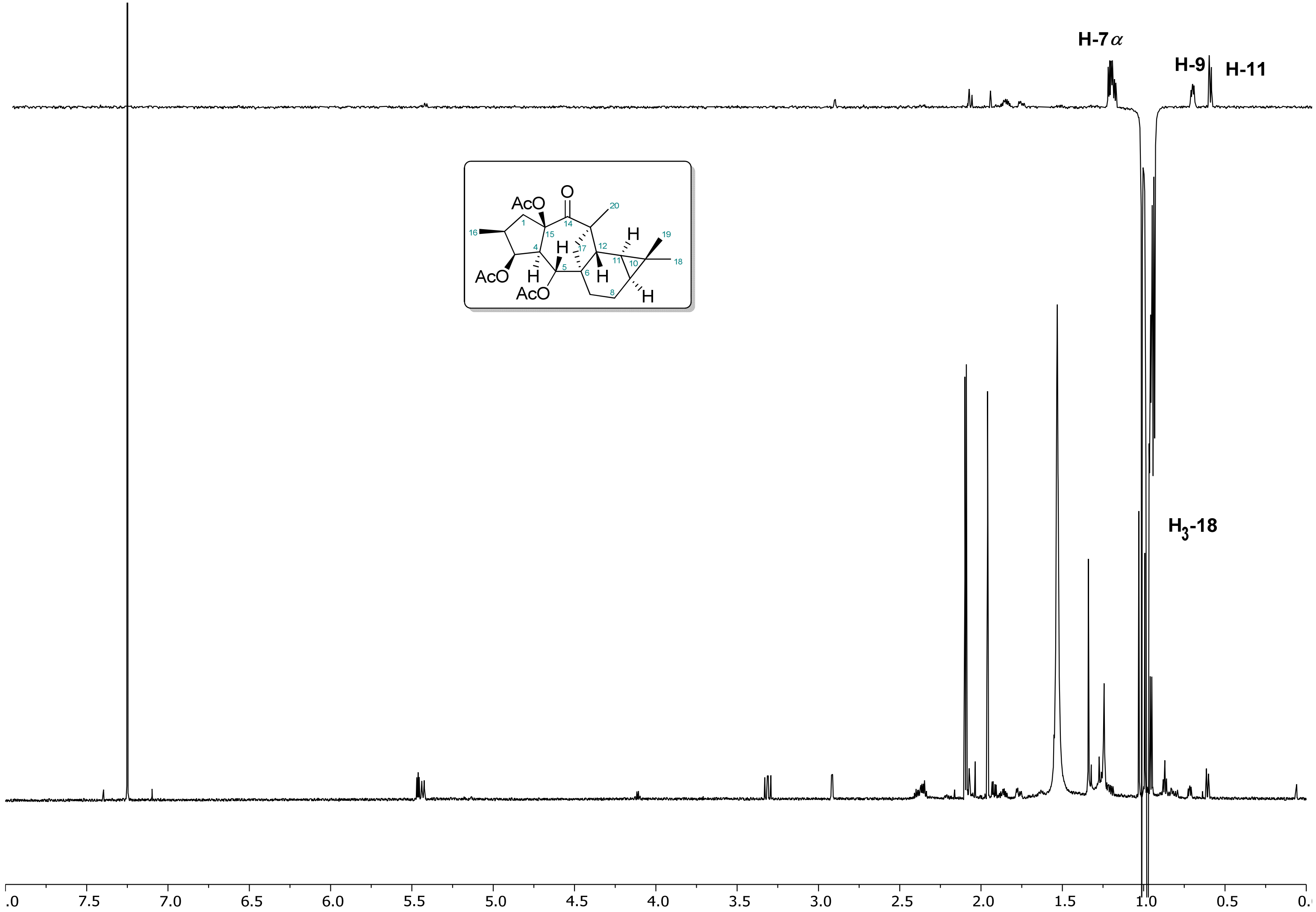

Figure S16c. 1D NOESY spectrum of compound **2**.

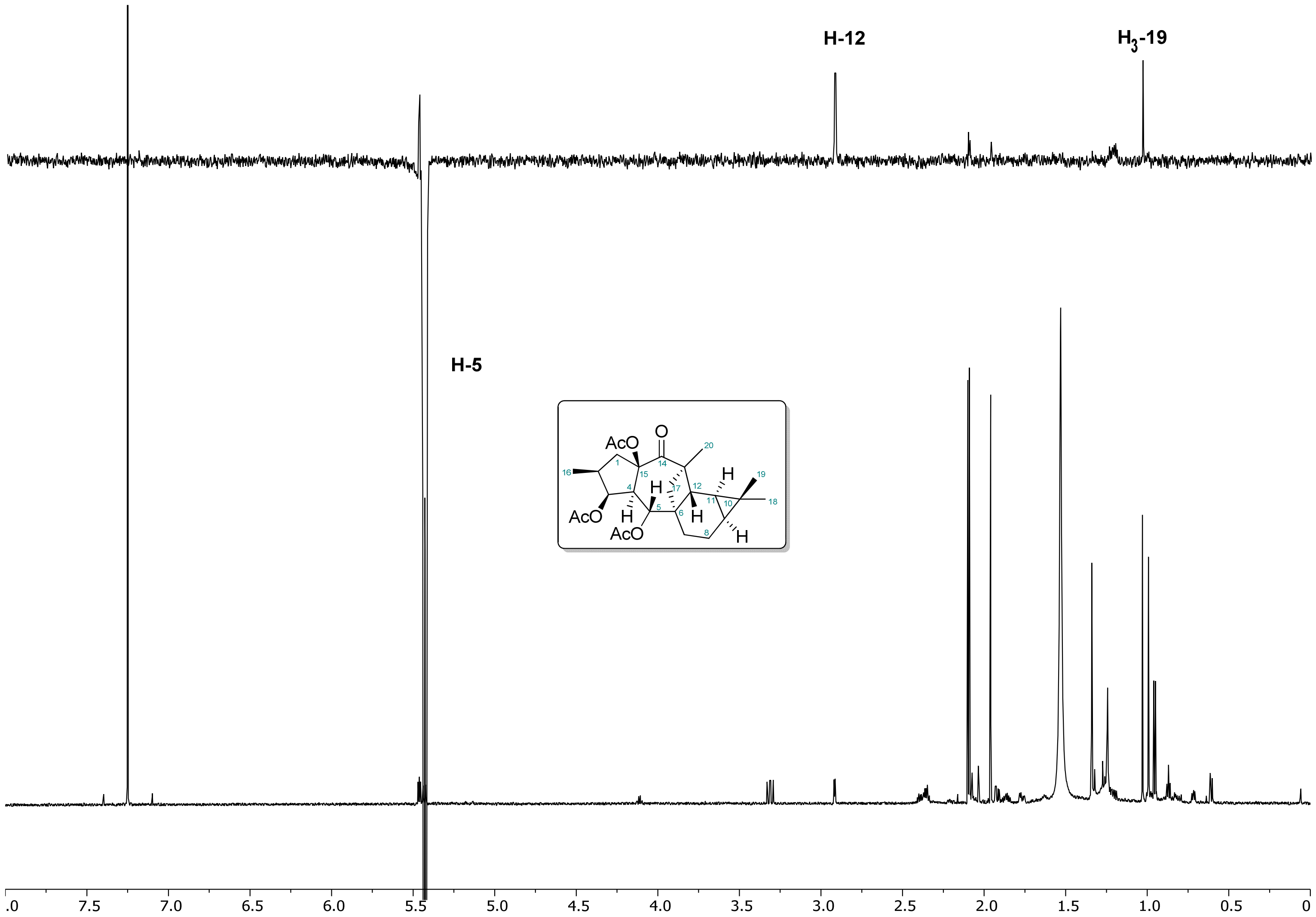

Figure S16d. 1D NOESY spectrum of compound **2**.

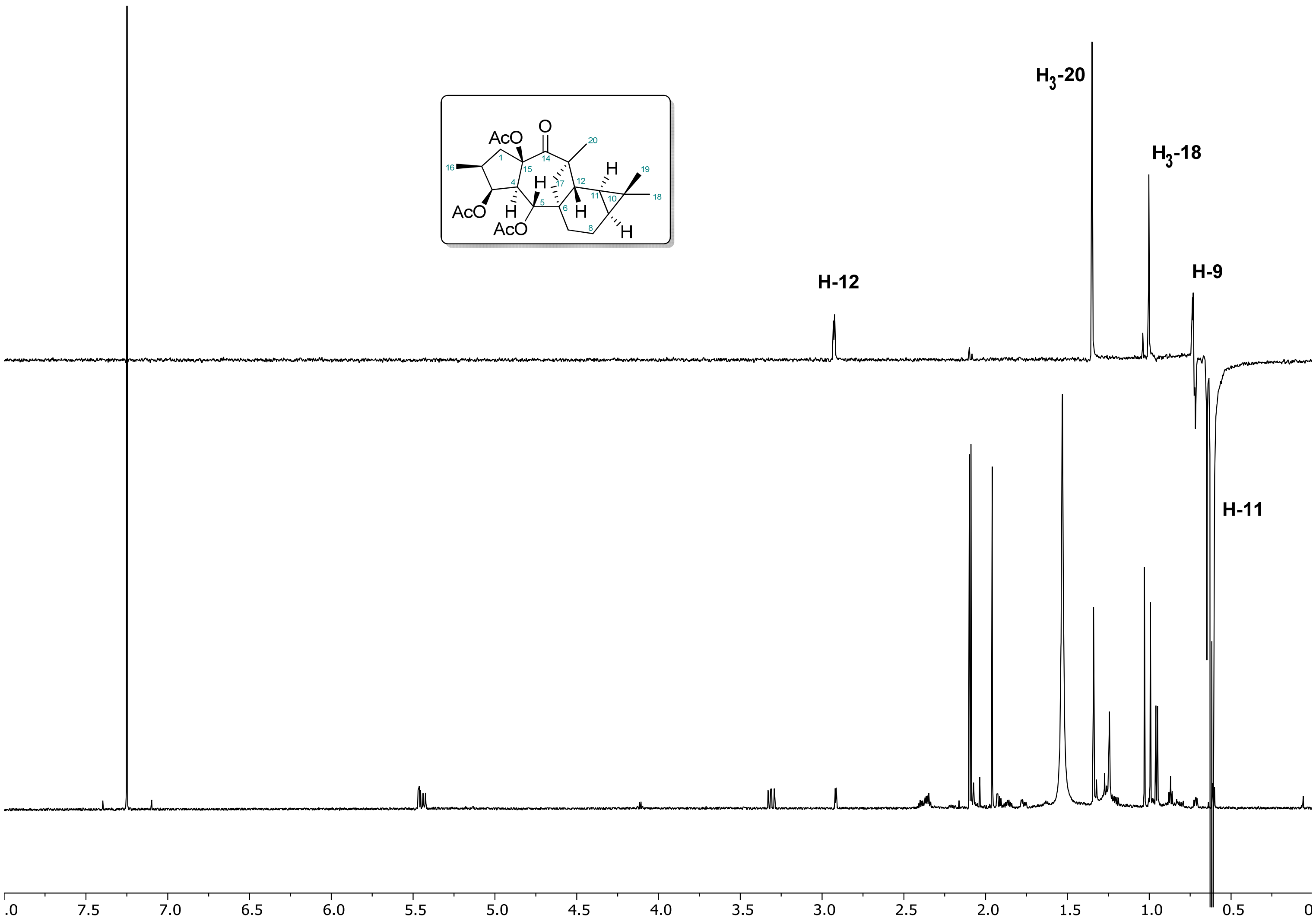

Figure S16e. 1D NOESY spectrum of compound **2**.

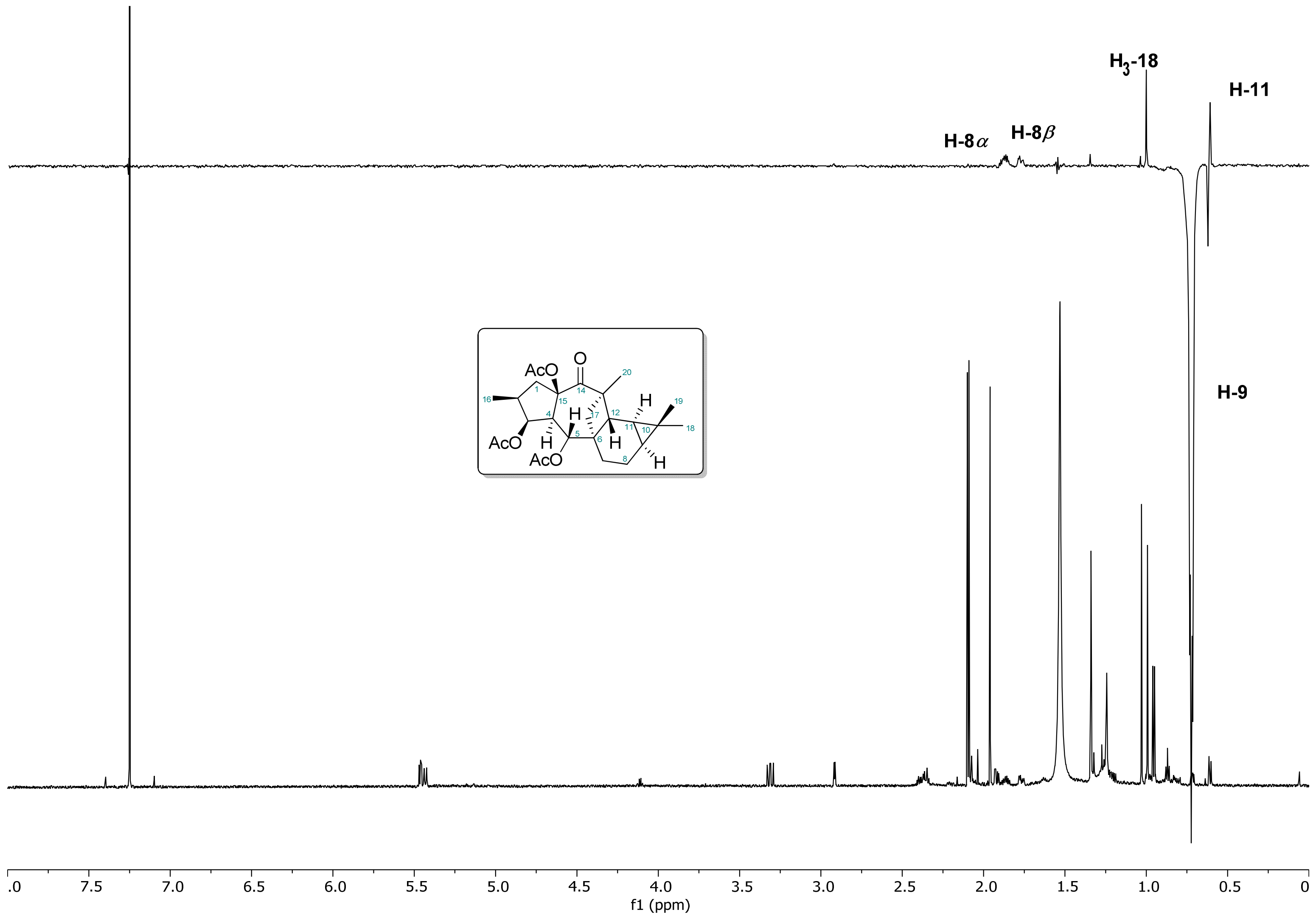

Figure S16f. 1D NOESY spectrum of compound **2**.

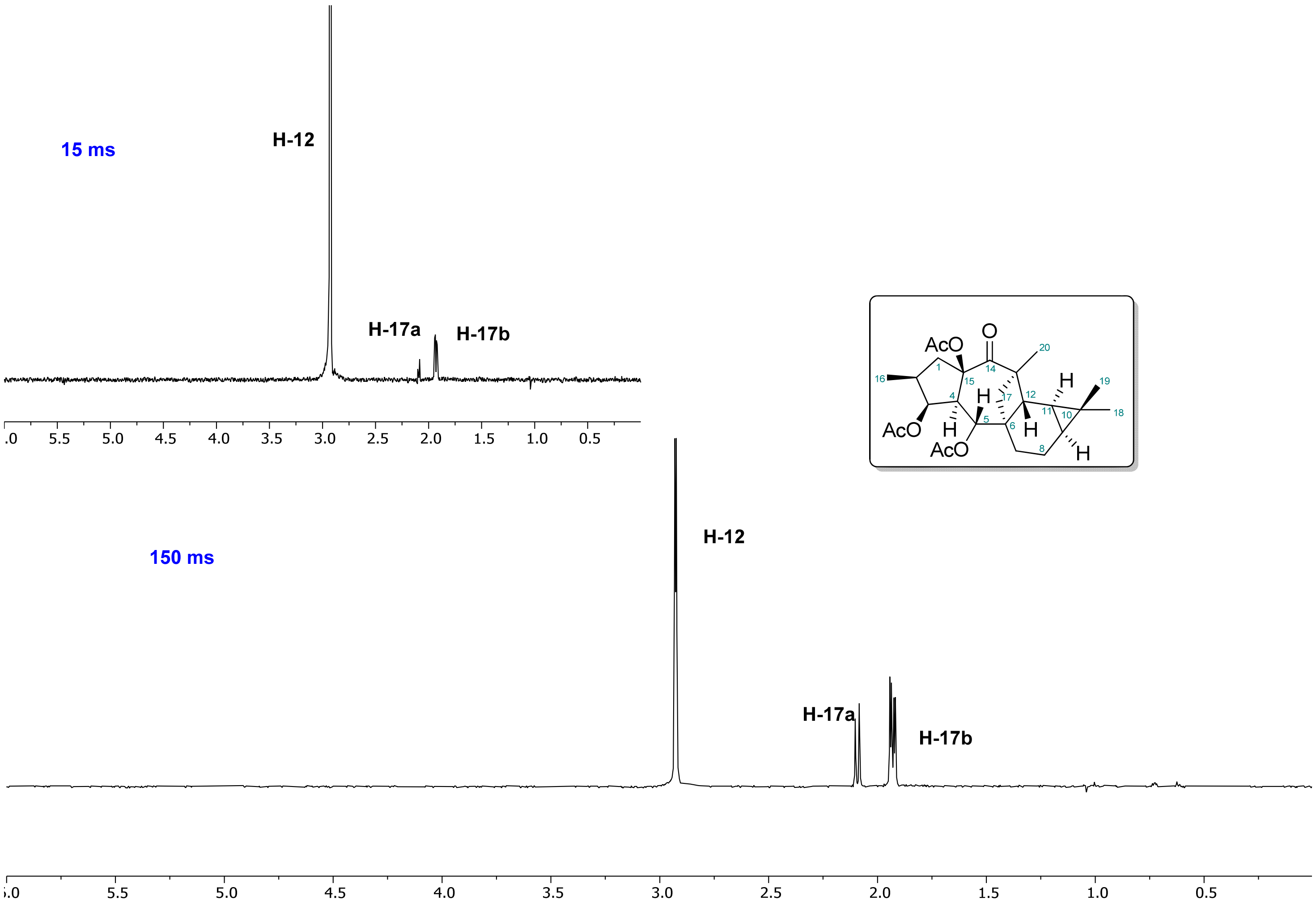

Figure S17a. 1D TOCSY spectrum of compound **2**.

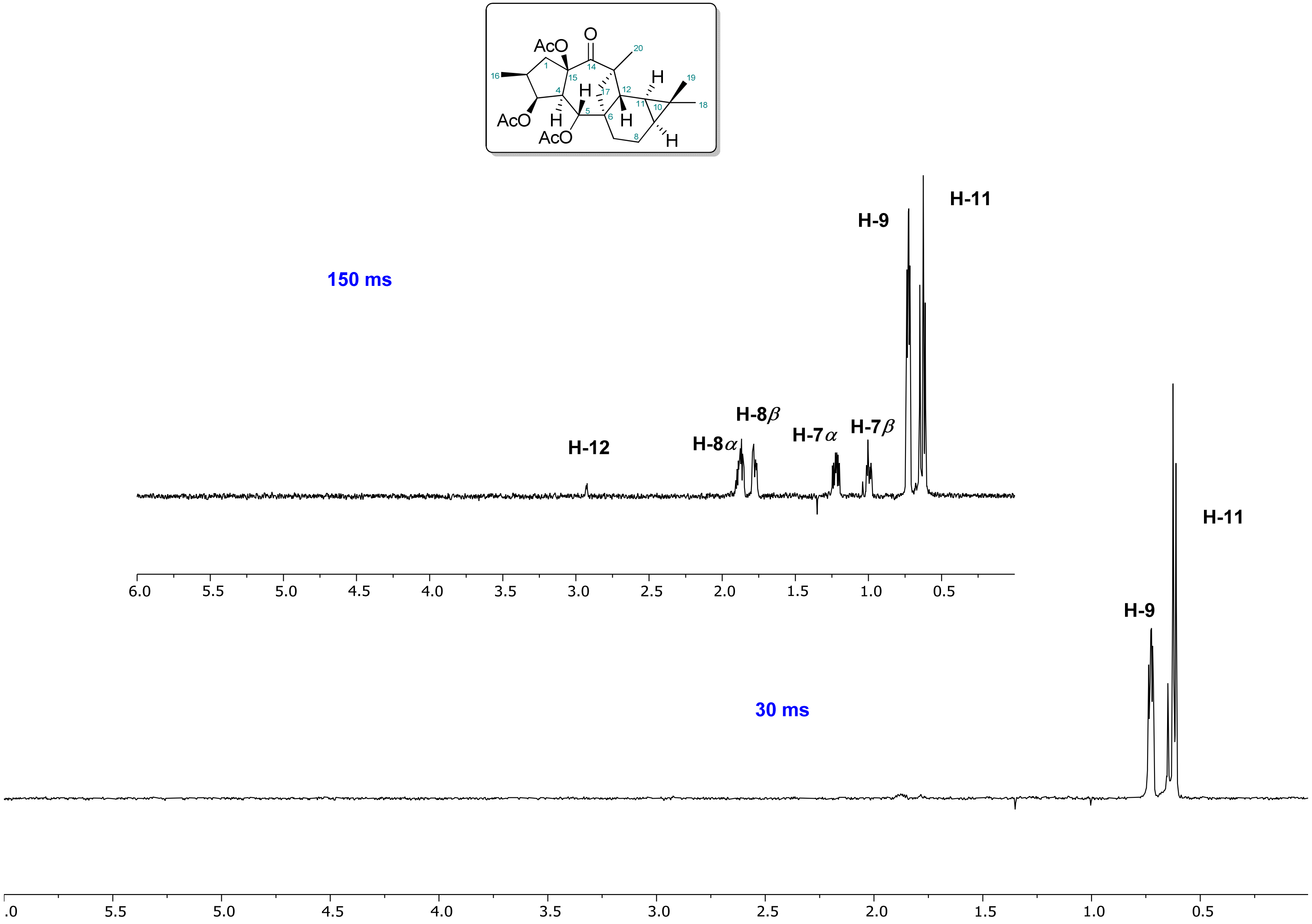

Figure S17b. 1D TOCSY spectrum of compound **2**.

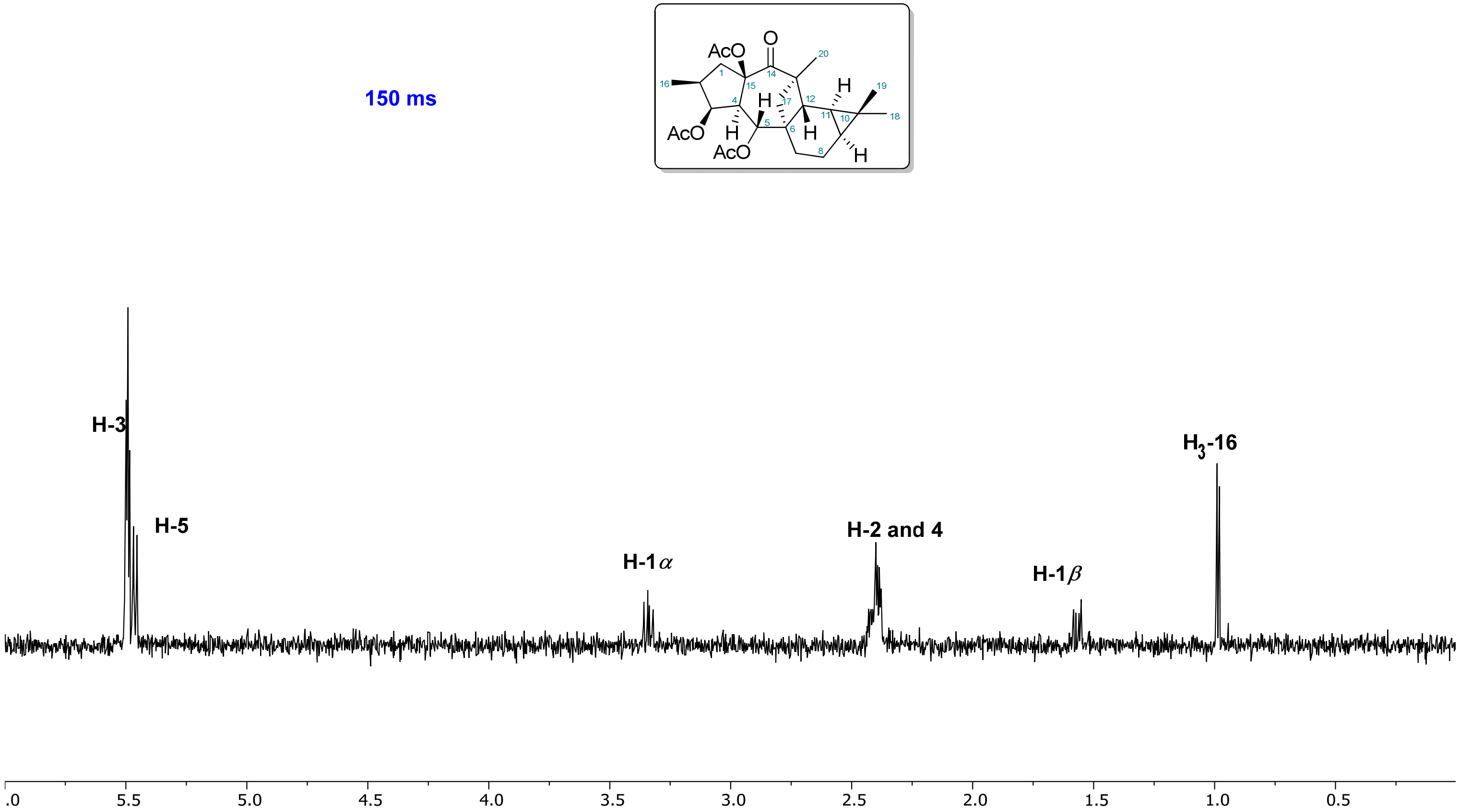

Figure S17c. 1D TOCSY spectrum of compound **2**.

Elemental Composition Report

Single Mass Analysis

Tolerance = 5.0 mDa / DBE: min = -1.5, max = 80.0
Element prediction: Off
Number of isotope peaks used for i-FIT = 5

Monoisotopic Mass, Even Electron Ions
93 formula(e) evaluated with 2 results within limits (all results (up to 1000) for each mass)
Elements Used:
C: 0-30 H: 0-50 O: 0-15 23Na: 0-1

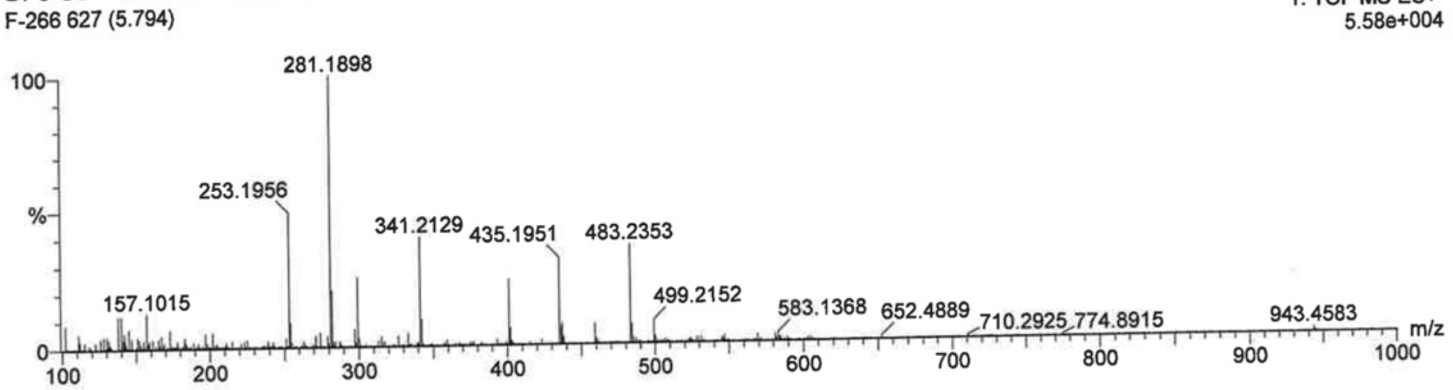

Minimum:				-1.5				
Maximum:		5.0	10.0	80.0				
Mass	Calc. Mass	mDa	PPM	DBE	i-FIT	Norm	Conf(%)	Formula
483.2353	483.2359	-0.6	-1.2	8.5	461.1	0.311	73.24	C26 H36 O7 23Na
	483.2383	-3.0	-6.2	11.5	462.1	1.318	26.76	C28 H35 O7

Figure S18. HRMS spectrum of compound **2**.

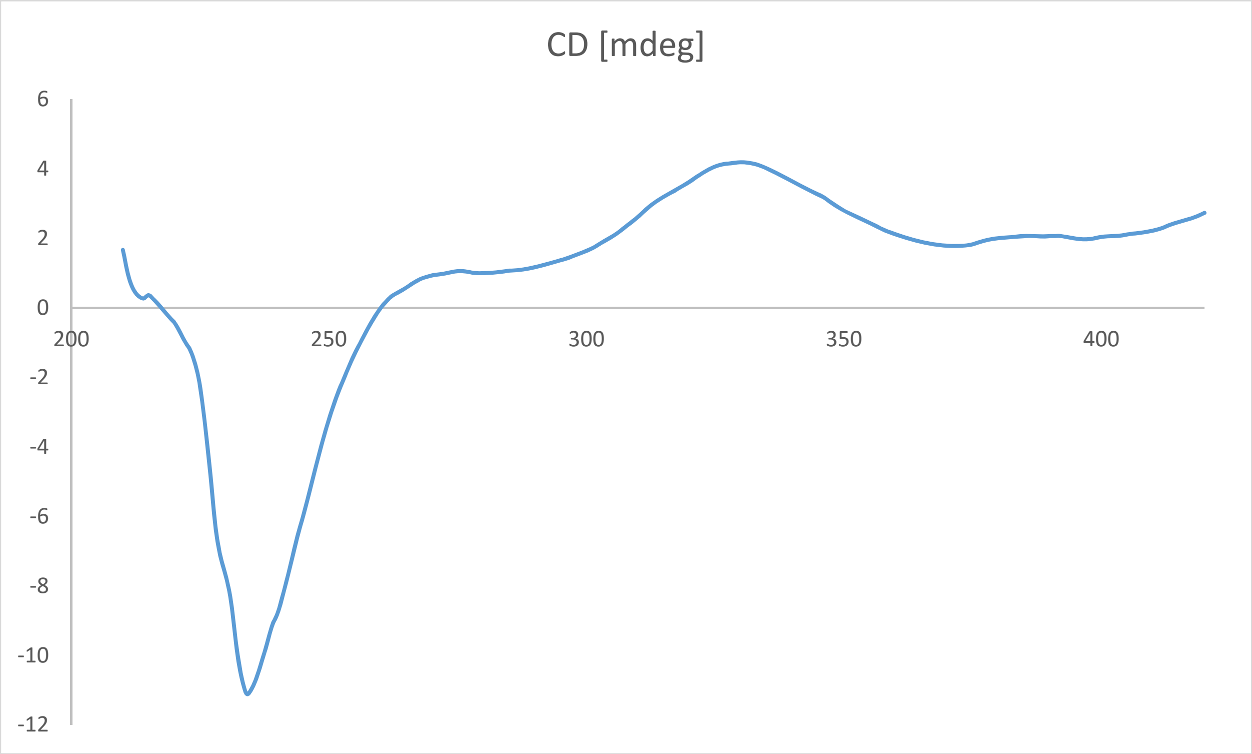

Figure S19. ECD spectrum of compound **2**.

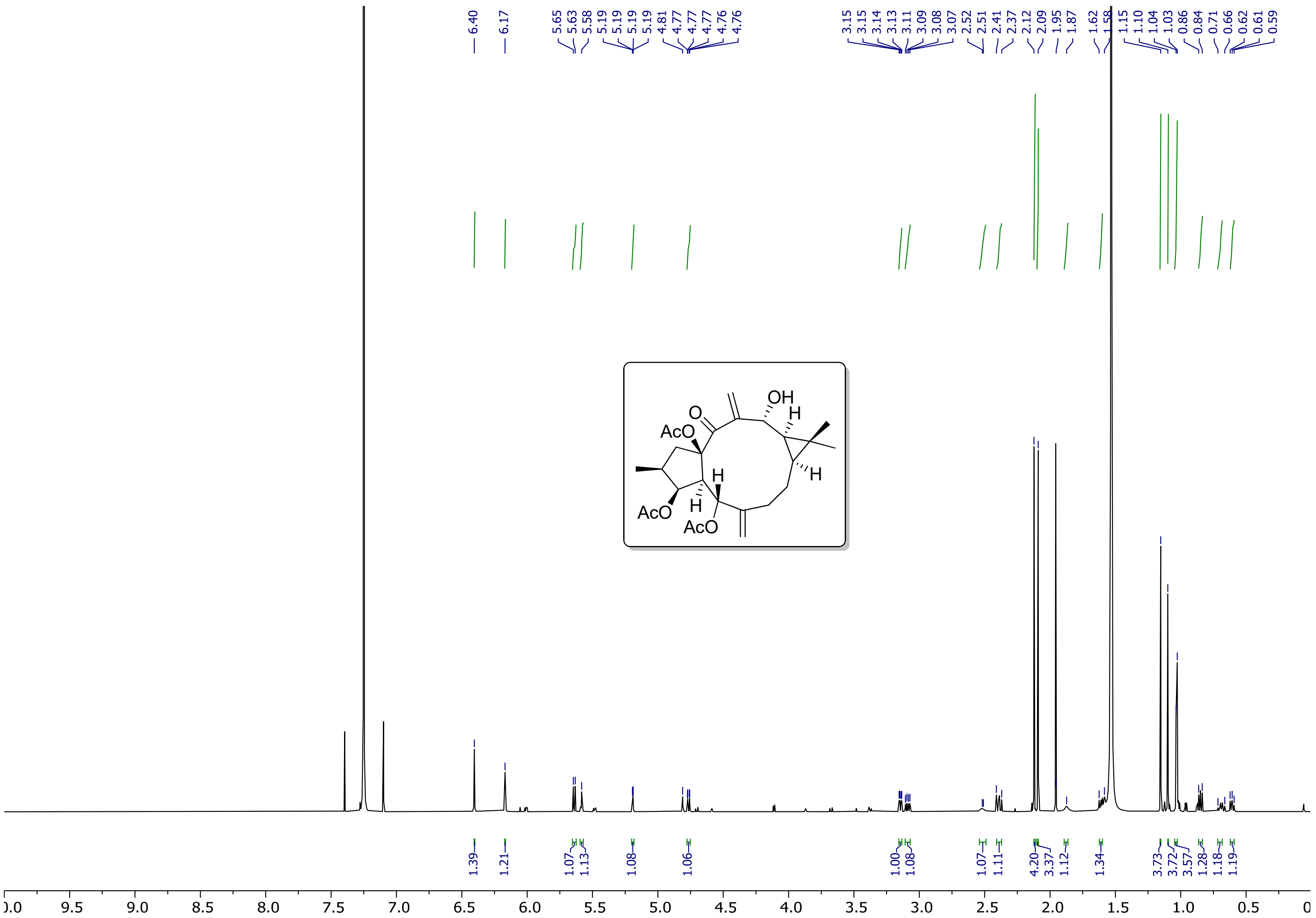

Figure S20. ^{1}H NMR spectrum (700 MHz) of compound **3** in $CDCl_3$.

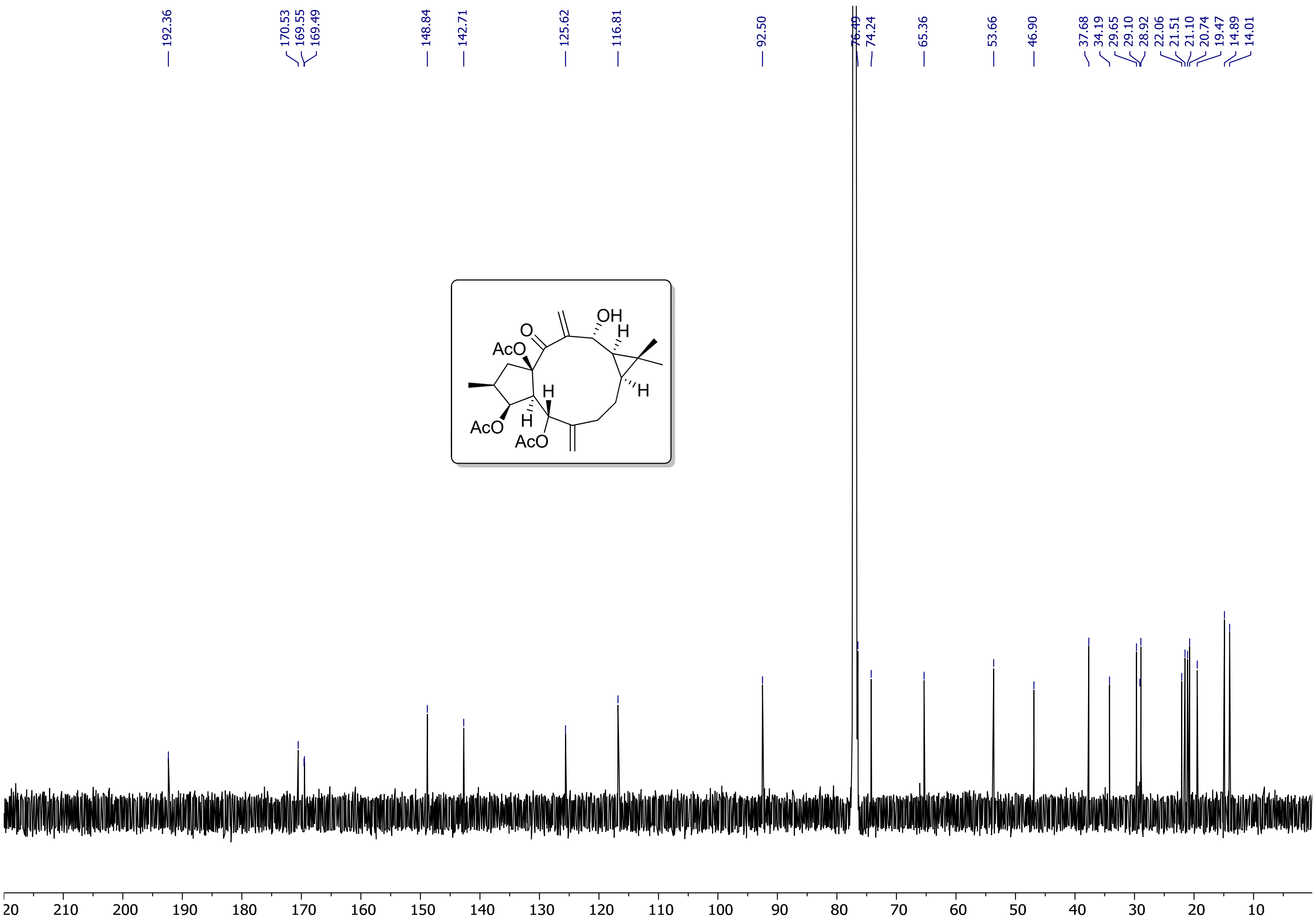

Figure S21. ^{13}C NMR spectrum (175 MHz) of compound **3** in $CDCl_3$.

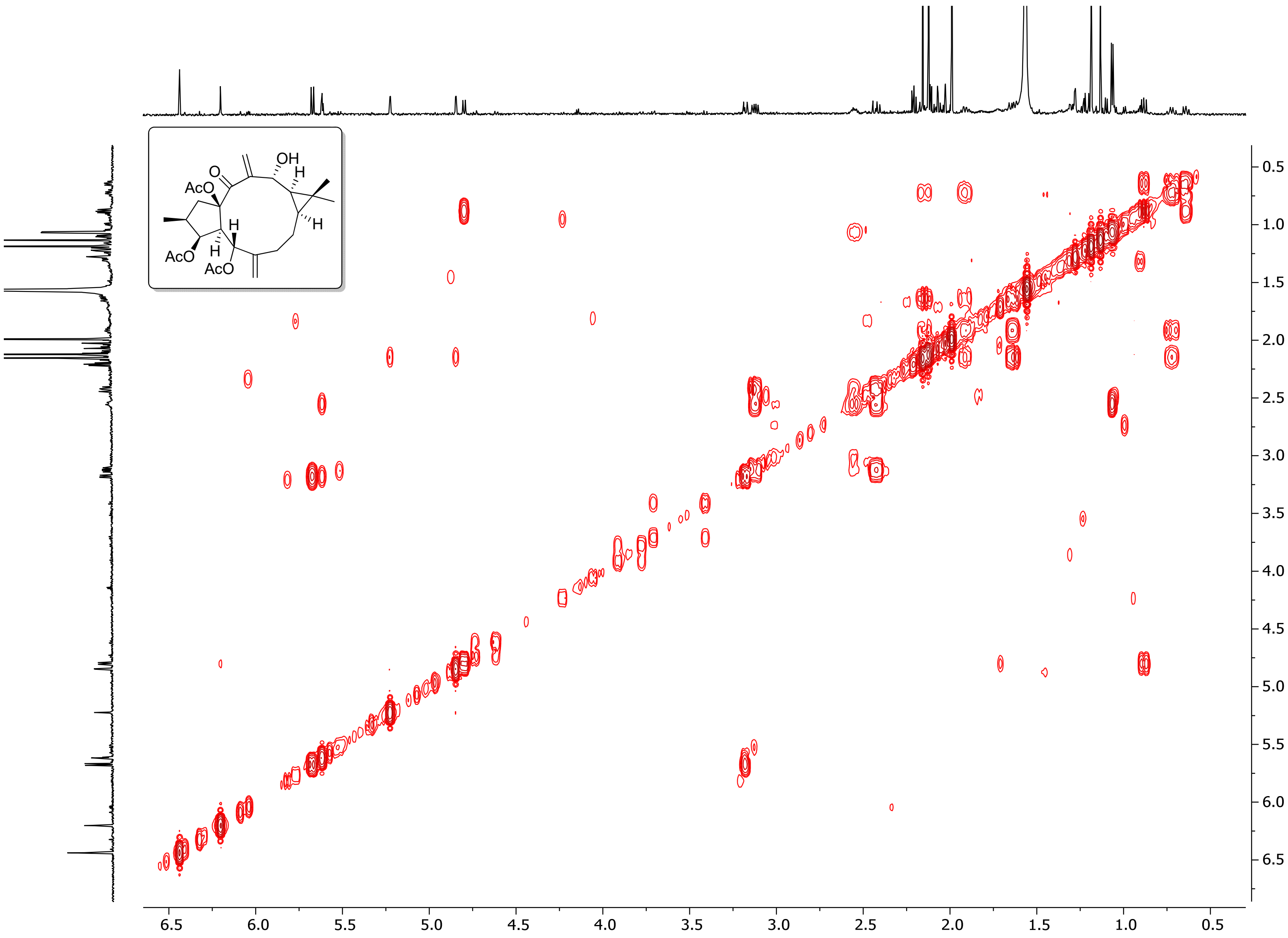

Figure S22. gCOSY spectrum of compound **3**.

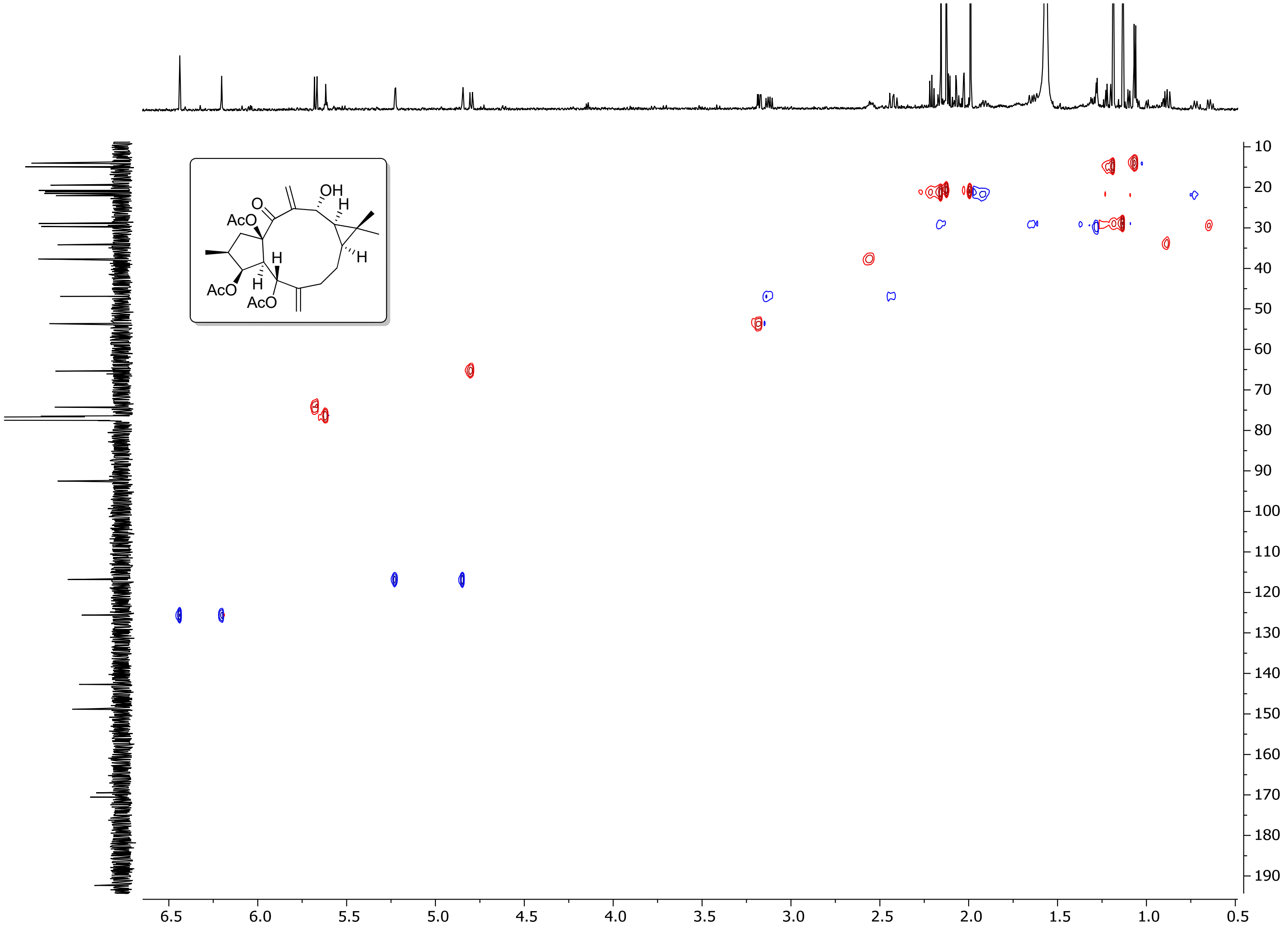

Figure S23. gHSQC spectrum of compound **3**.

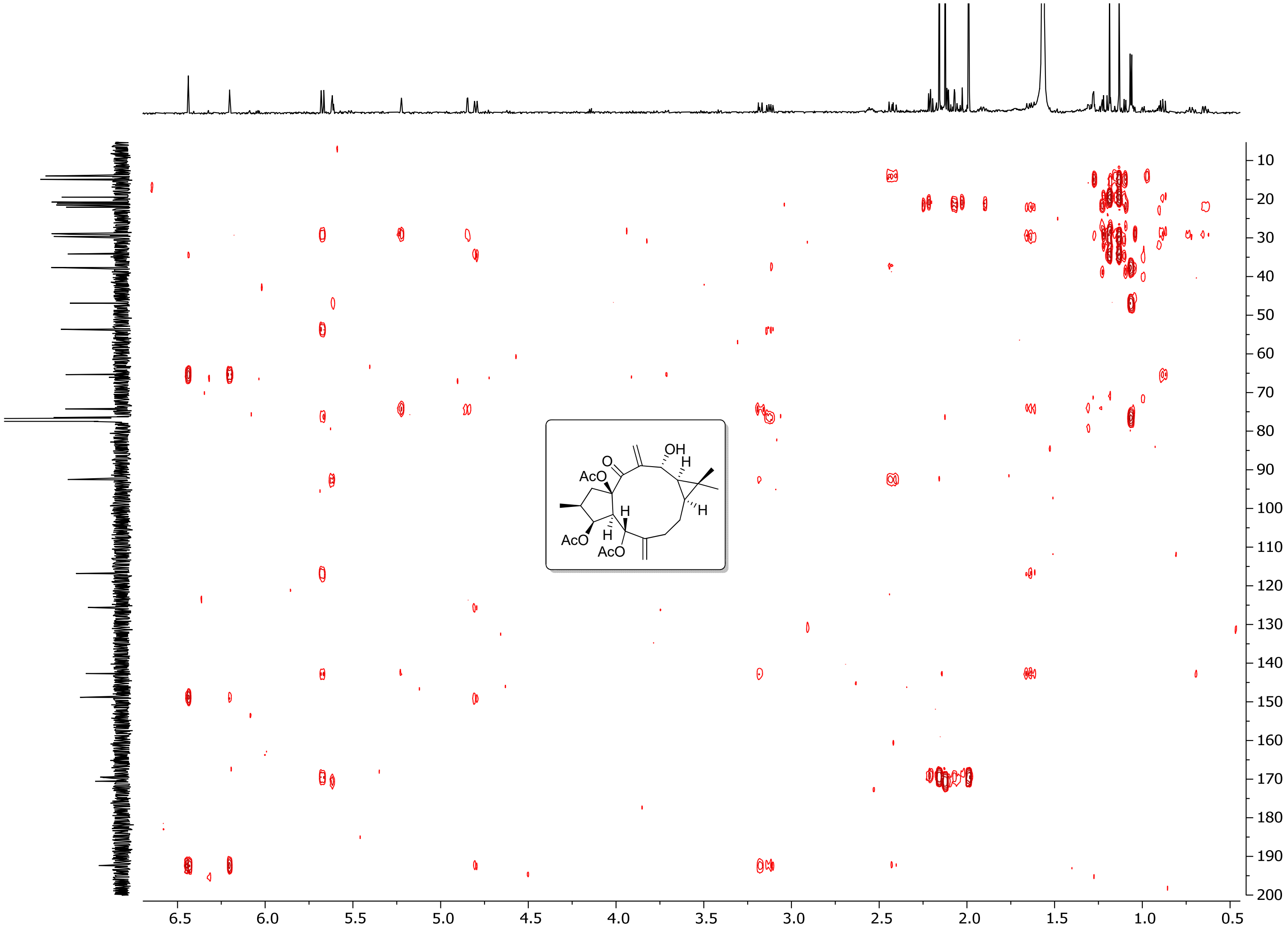

Figure S24. gHMBC spectrum of compound **3**.

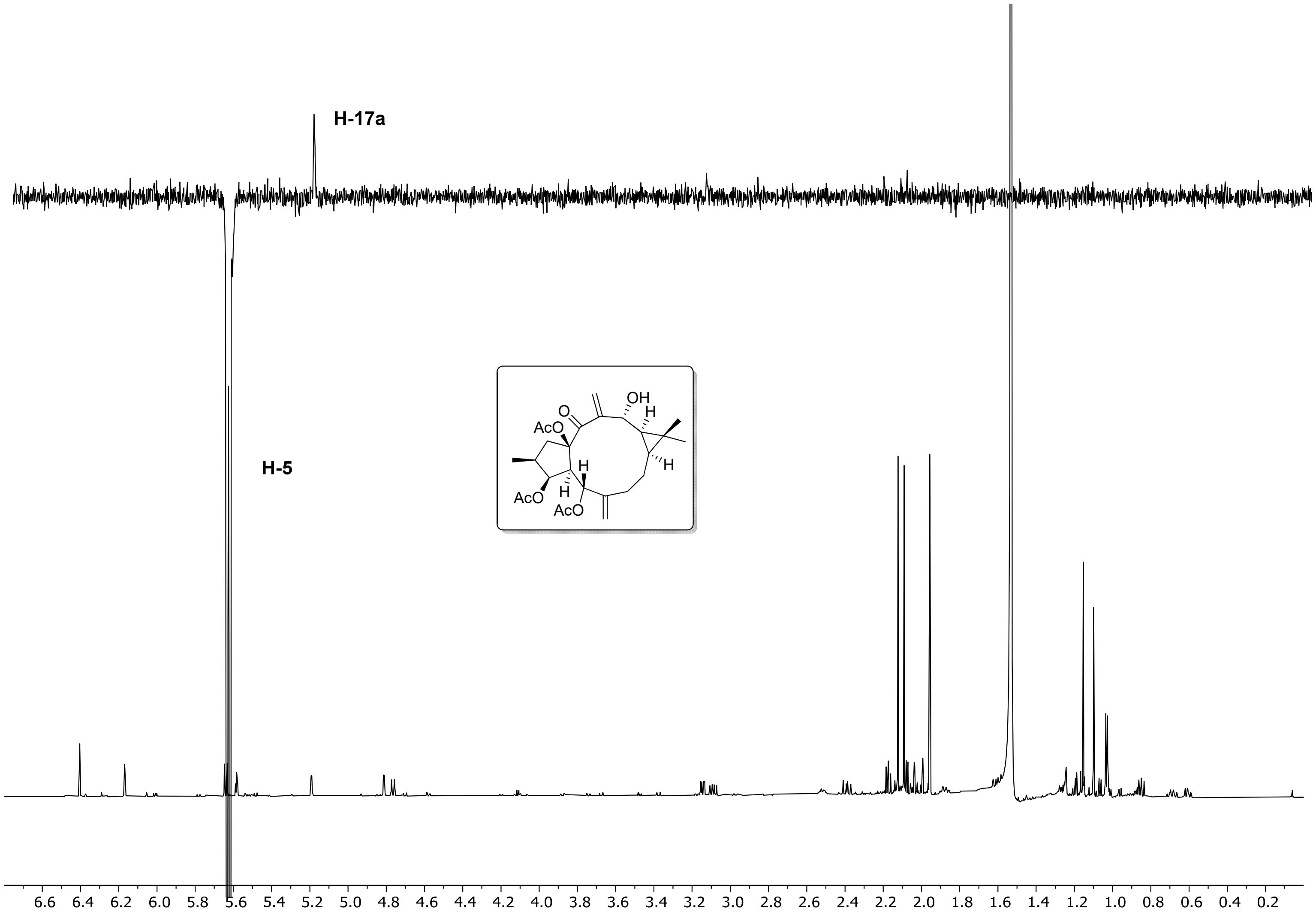

Figure S25a. 1D NOESY spectrum of compound **3**.

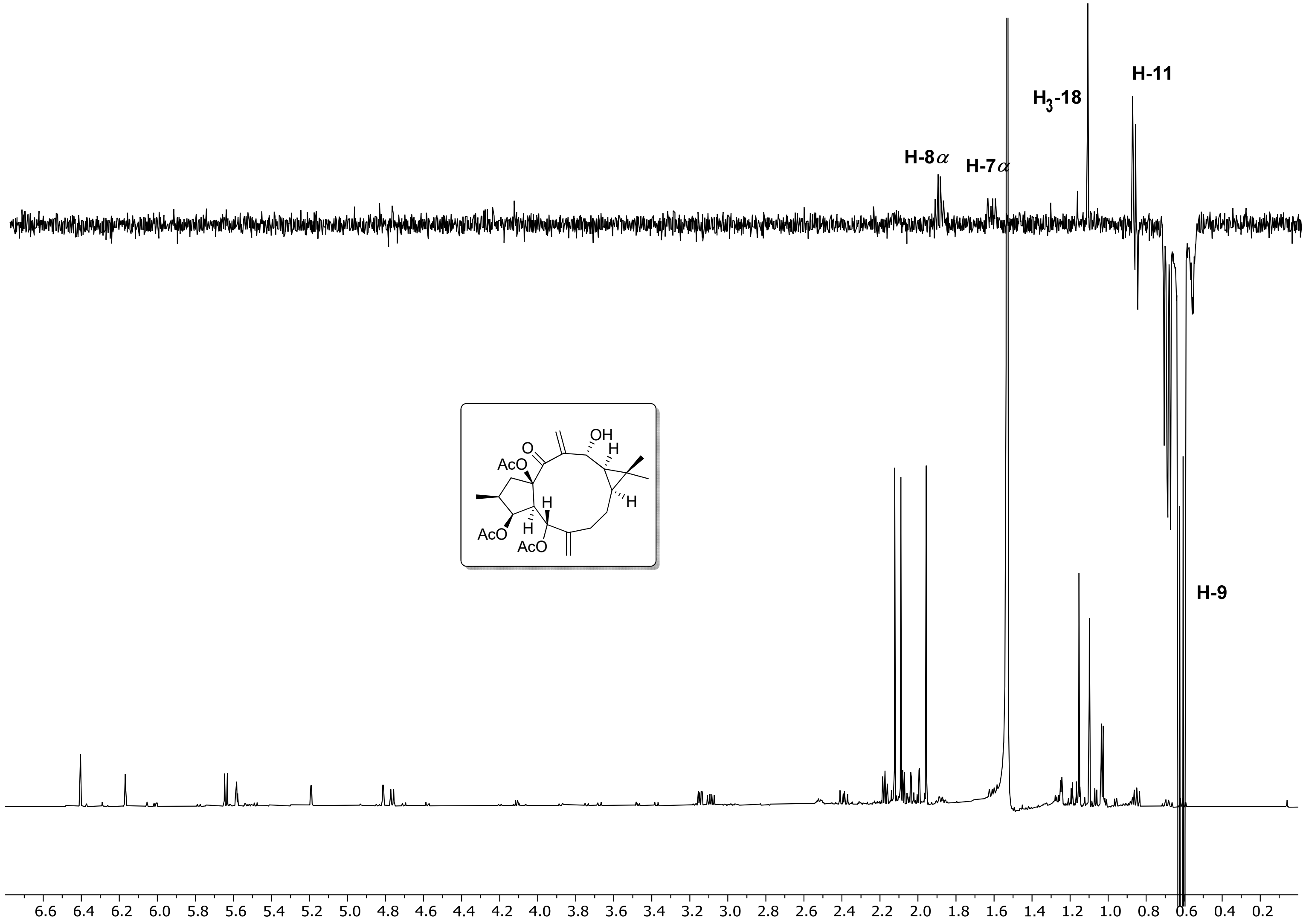

Figure S25b. 1D NOESY spectrum of compound **3**.

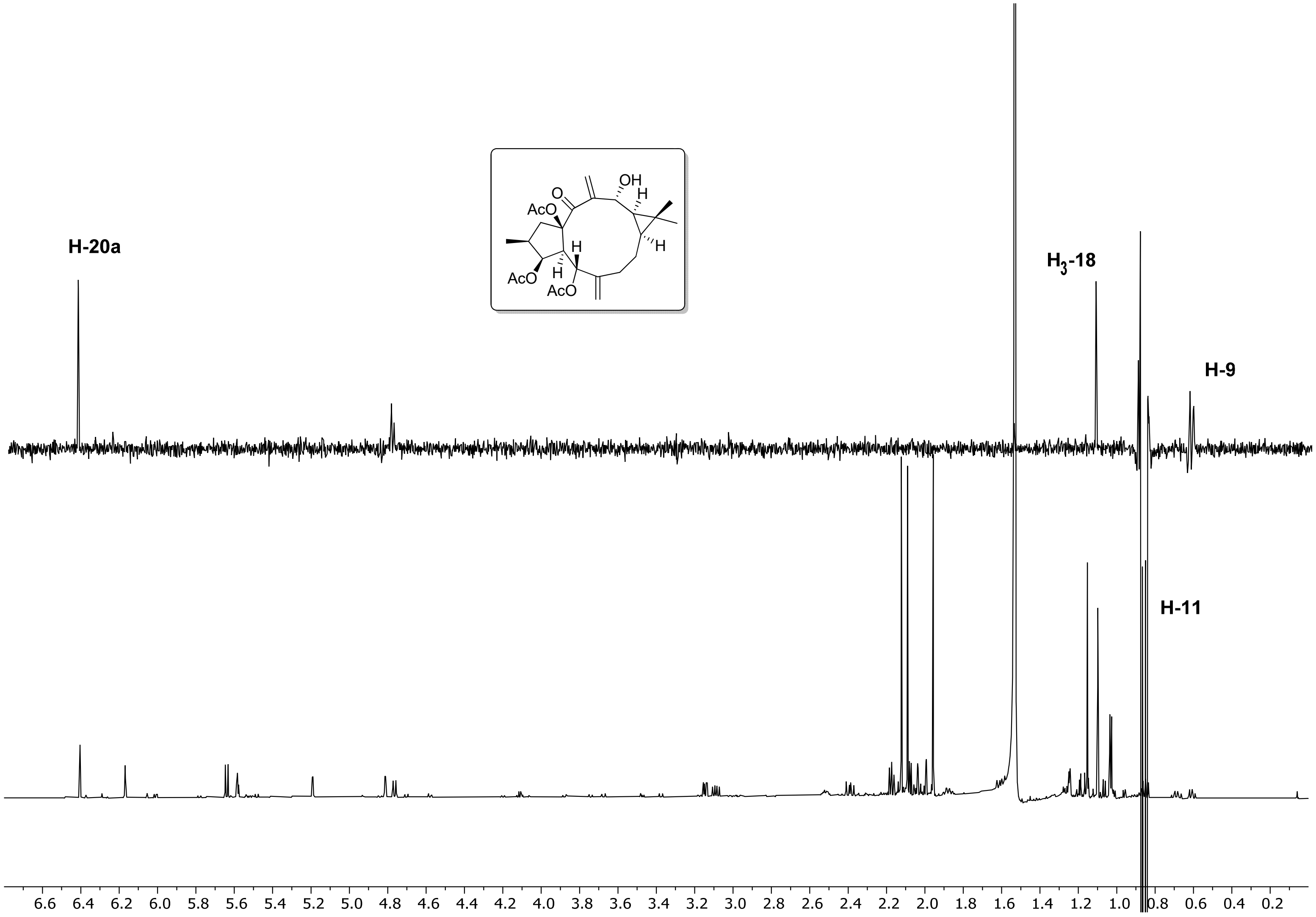

Figure S25c. 1D NOESY spectrum of compound **3**.

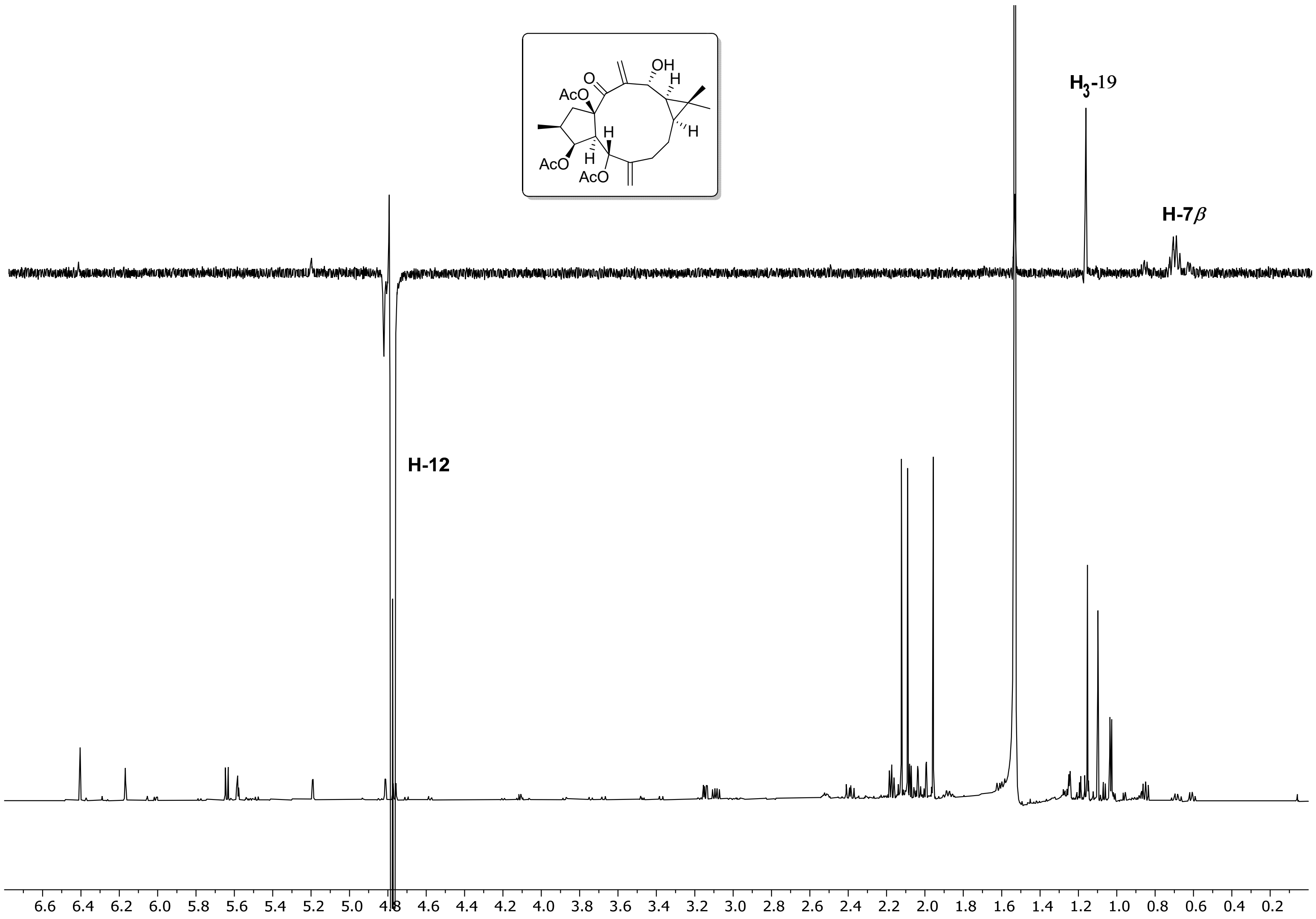

Figure S25d. 1D NOESY spectrum of compound **3**.

Elemental Composition Report

Page 1

Single Mass Analysis

Tolerance = 3.0 mDa / DBE: min = -1.5, max = 50.0
Element prediction: Off
Number of isotope peaks used for i-FIT = 2

Monoisotopic Mass, Even Electron Ions
389 formula(e) evaluated with 1 results within limits (up to 5 closest results for each mass)
Elements Used:
C: 0-500 H: 0-1000 O: 0-20 Na: 0-1 I: 0-5

FELIPE ESCOBAR en ACN
F-265 572 (5.288)

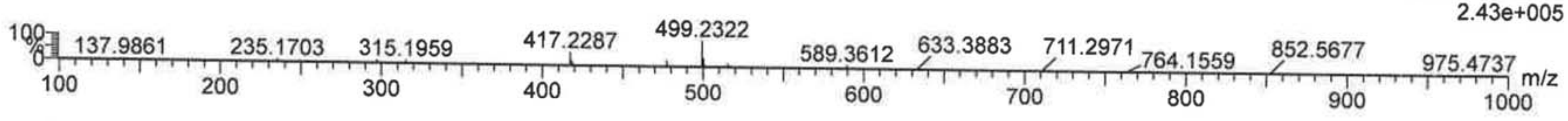

Minimum:				-1.5				
Maximum:		3.0	10.0	50.0				
Mass	Calc. Mass	mDa	PPM	DBE	i-FIT	Norm	Conf(%)	Formula
477.2505	477.2488	1.7	3.6	8.5	207.7	n/a	n/a	C26 H37 O8

Figure S26. HRMS spectrum of compound **3**.

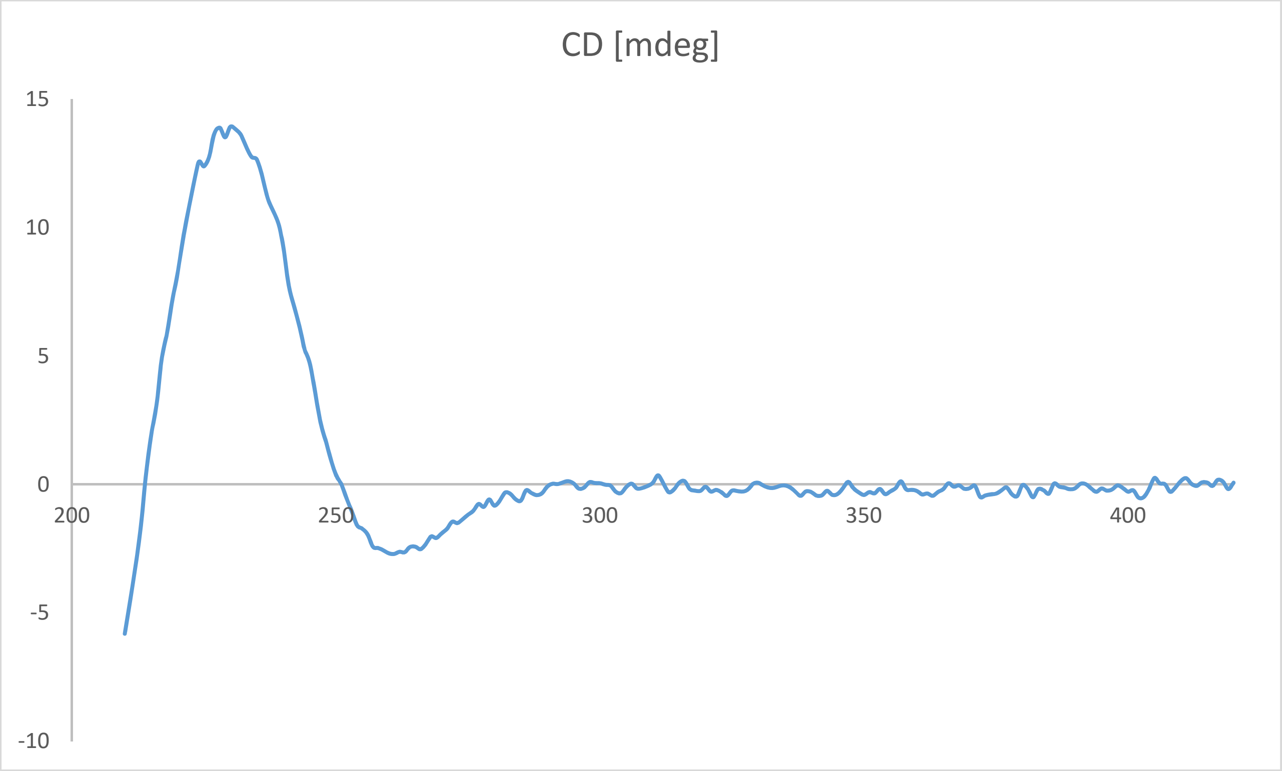

Figure S27. ECD spectrum of compound **3**.

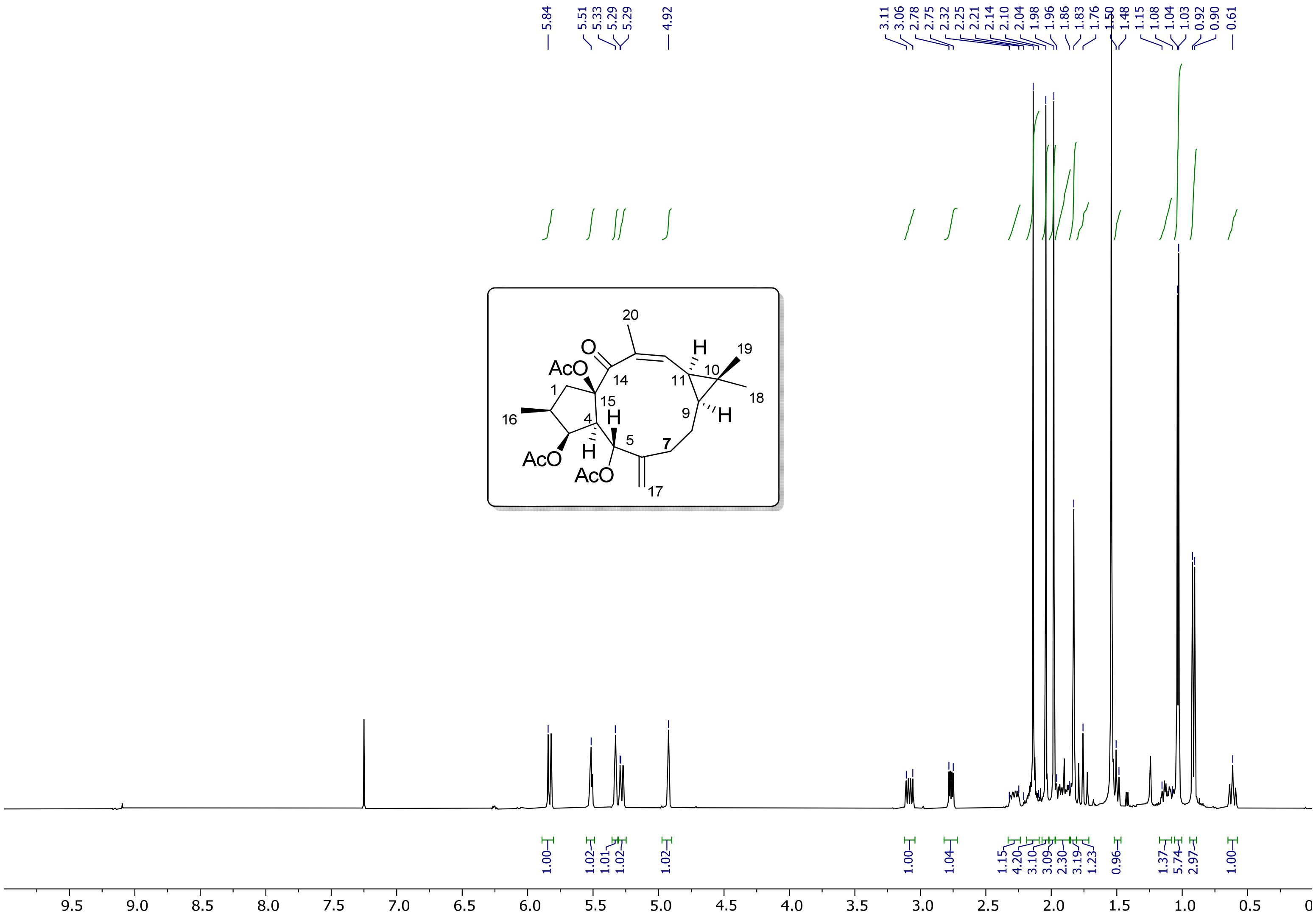

Figure S28. ^{1}H NMR spectrum (400 MHz) of compound **4** in $CDCl_3$.

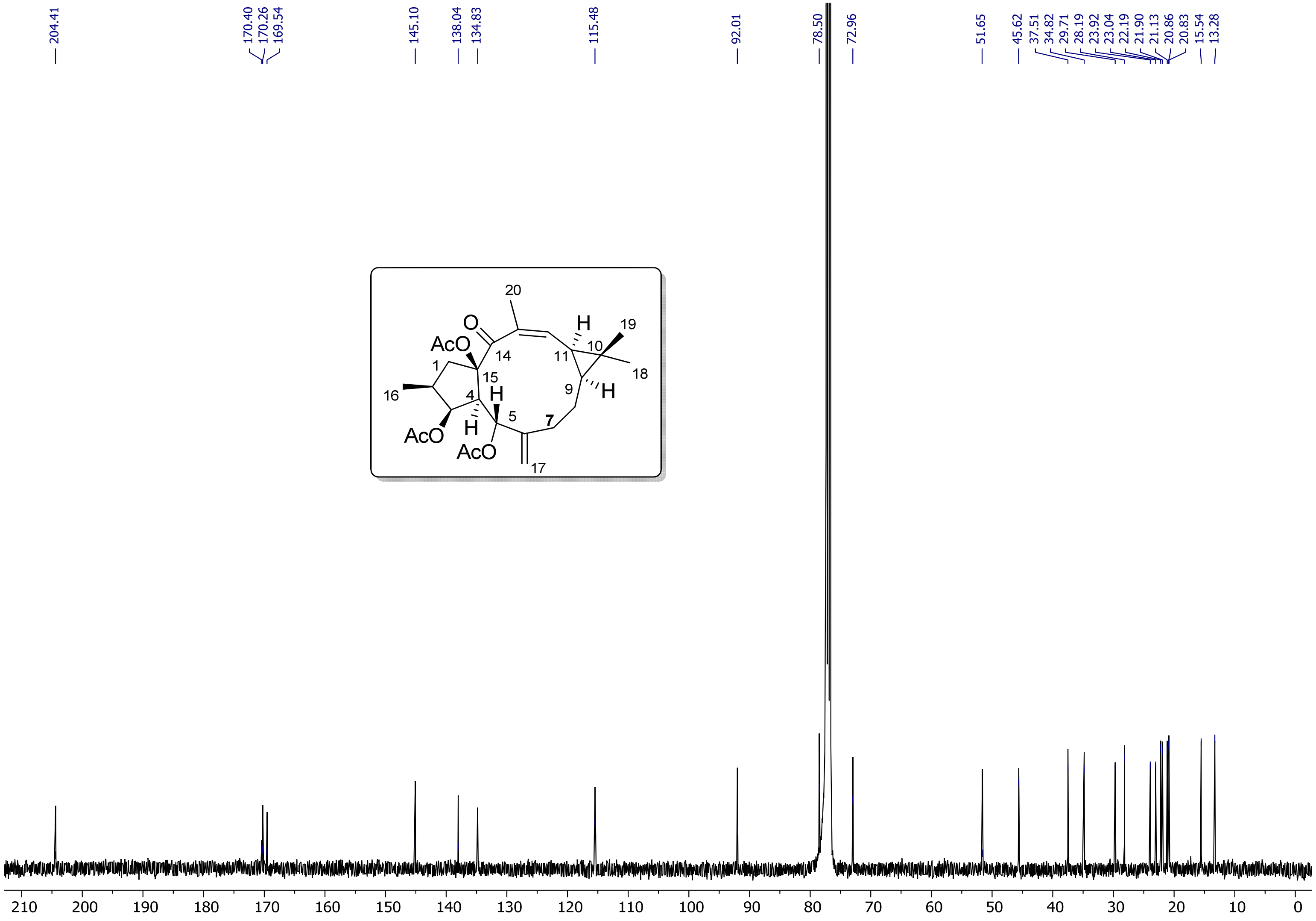

Figure S29. ^{13}C NMR spectrum (100 MHz) of compound **4** in $CDCl_3$.

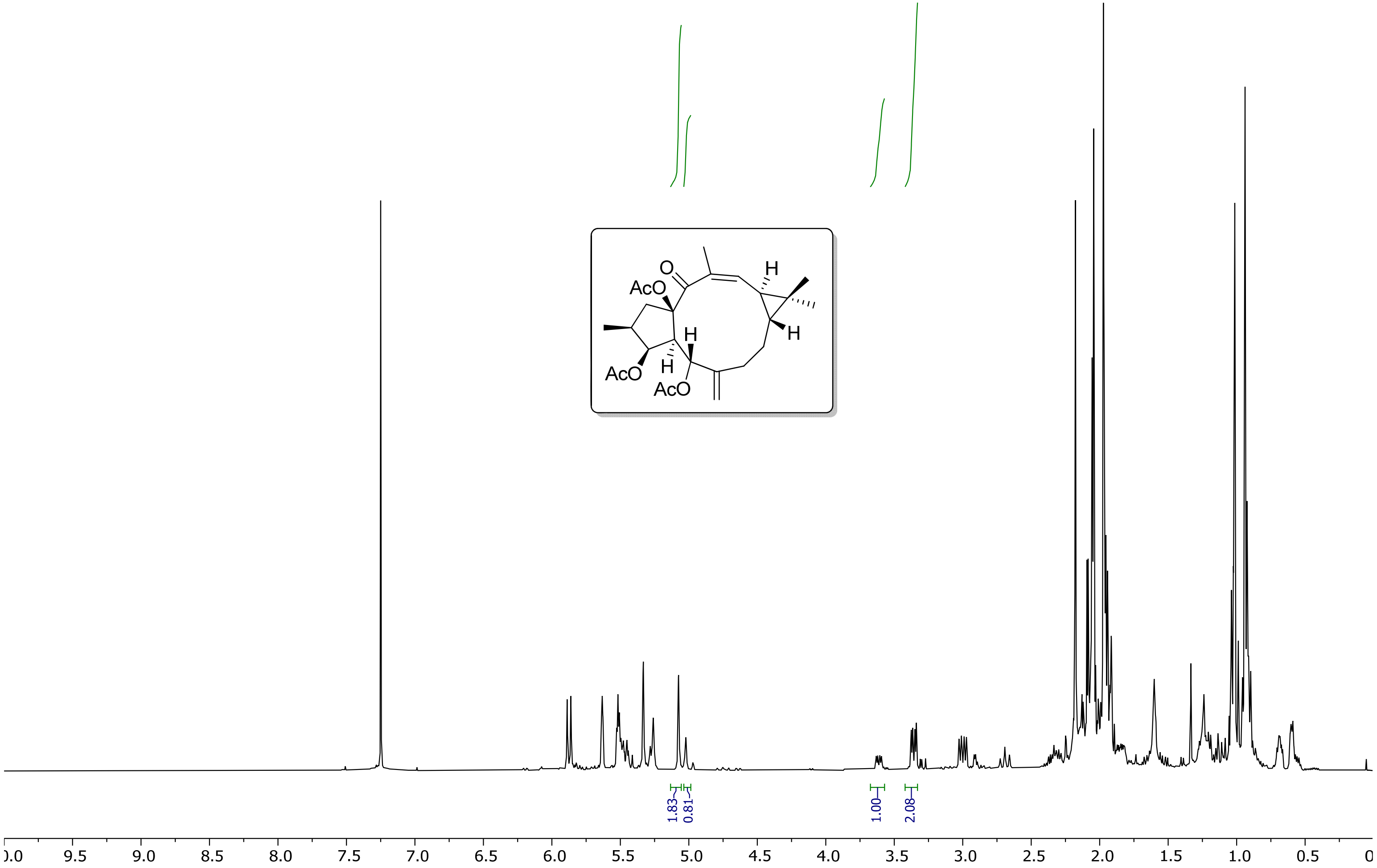

Figure S30. ^{1}H NMR spectrum (400 MHz) of mixture of atropoisomers **5** in $CDCl_3$.

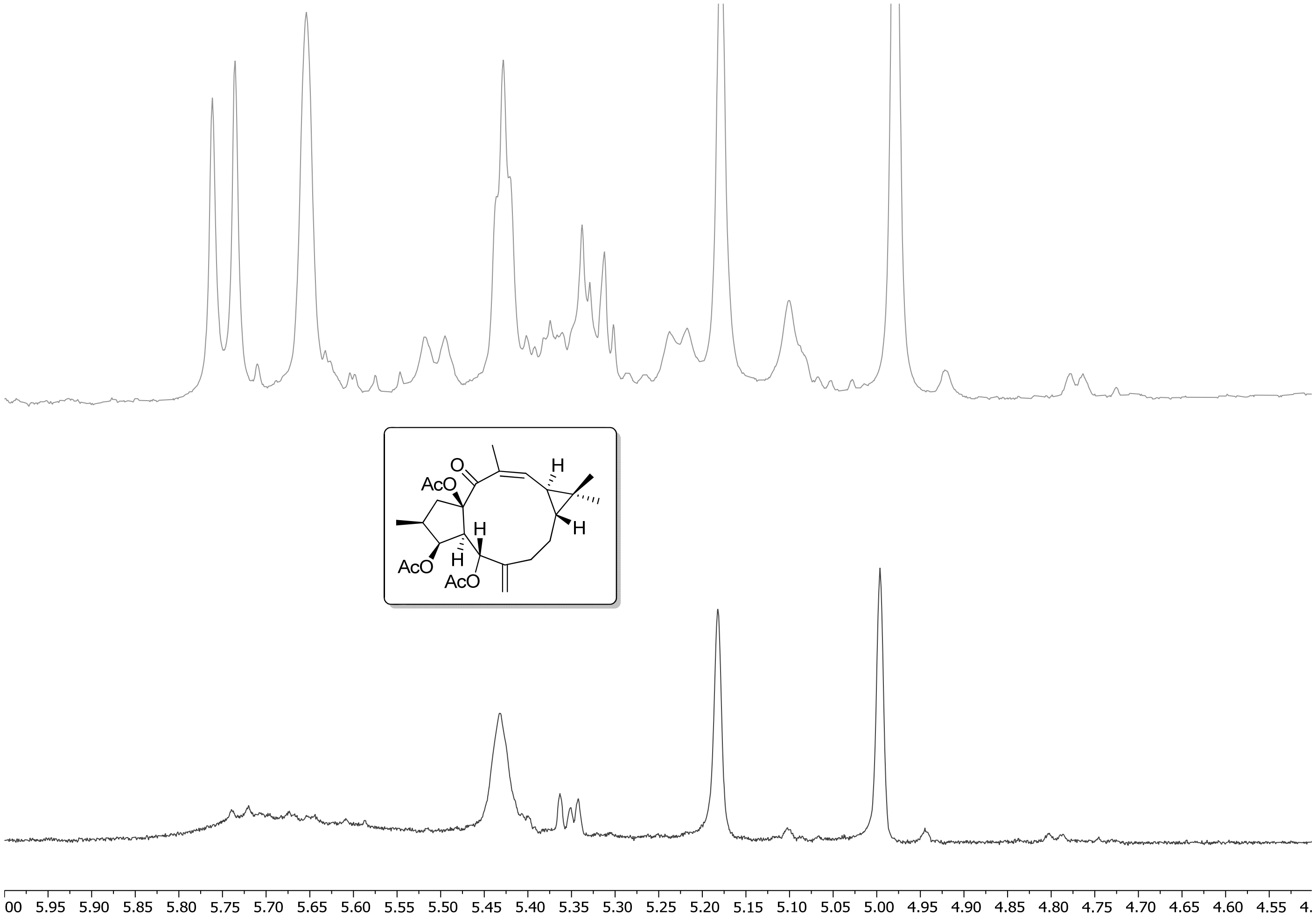

Figure S31. ^{1}H NMR spectrum (400 MHz) of mixture of atropoisomers **5** in DMSO-d_6 (25ºC for upper spectrum and 80ºC for lower spectrum).

Elemental Composition Report

Single Mass Analysis
Tolerance = 5.0 mDa / DBE: min = -1.5, max = 50.0
Element prediction: Off
Number of isotope peaks used for i-FIT = 3

Monoisotopic Mass, Even Electron Ions
1119 formula(e) evaluated with 8 results within limits (up to 50 best isotopic matches for each mass)
Elements Used:
C: 0-500 H: 0-1000 O: 0-200 Na: 0-1 Cl: 0-8
EB12--Benz-5--MSe2pos 321 (2.607)
2: TOF MS ES+

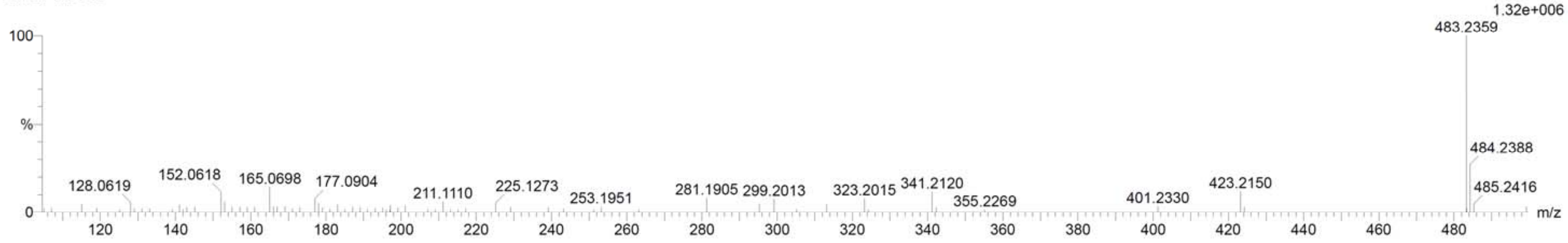

Minimum:				-1.5				
Maximum:		5.0	10.0	50.0				
Mass	Calc. Mass	mDa	PPM	DBE	i-FIT	Norm	Conf(%)	Formula
483.2359	483.2359	0.0	0.0	8.5	43.6	0.016	98.41	C26 H36 O7 Na
	483.2383	-2.4	-5.0	11.5	47.7	4.156	1.57	C28 H35 O7
	483.2324	3.5	7.2	20.5	52.2	8.579	0.02	C35 H31 O2
	483.2361	-0.2	-0.4	2.5	65.6	21.992	0.00	C22 H40 O9 Cl
	483.2337	2.2	4.6	-0.5	65.7	22.118	0.00	C20 H41 O9 Na Cl
	483.2409	-5.0	-10.3	3.5	66.9	23.306	0.00	C25 H42 O3 Na Cl2
	483.2352	0.7	1.4	6.5	67.4	23.785	0.00	C28 H42 Cl3
	483.2328	3.1	6.4	3.5	67.5	23.880	0.00	C26 H43 Na Cl3

Figure S32. HRMS spectrum of compound **5**.

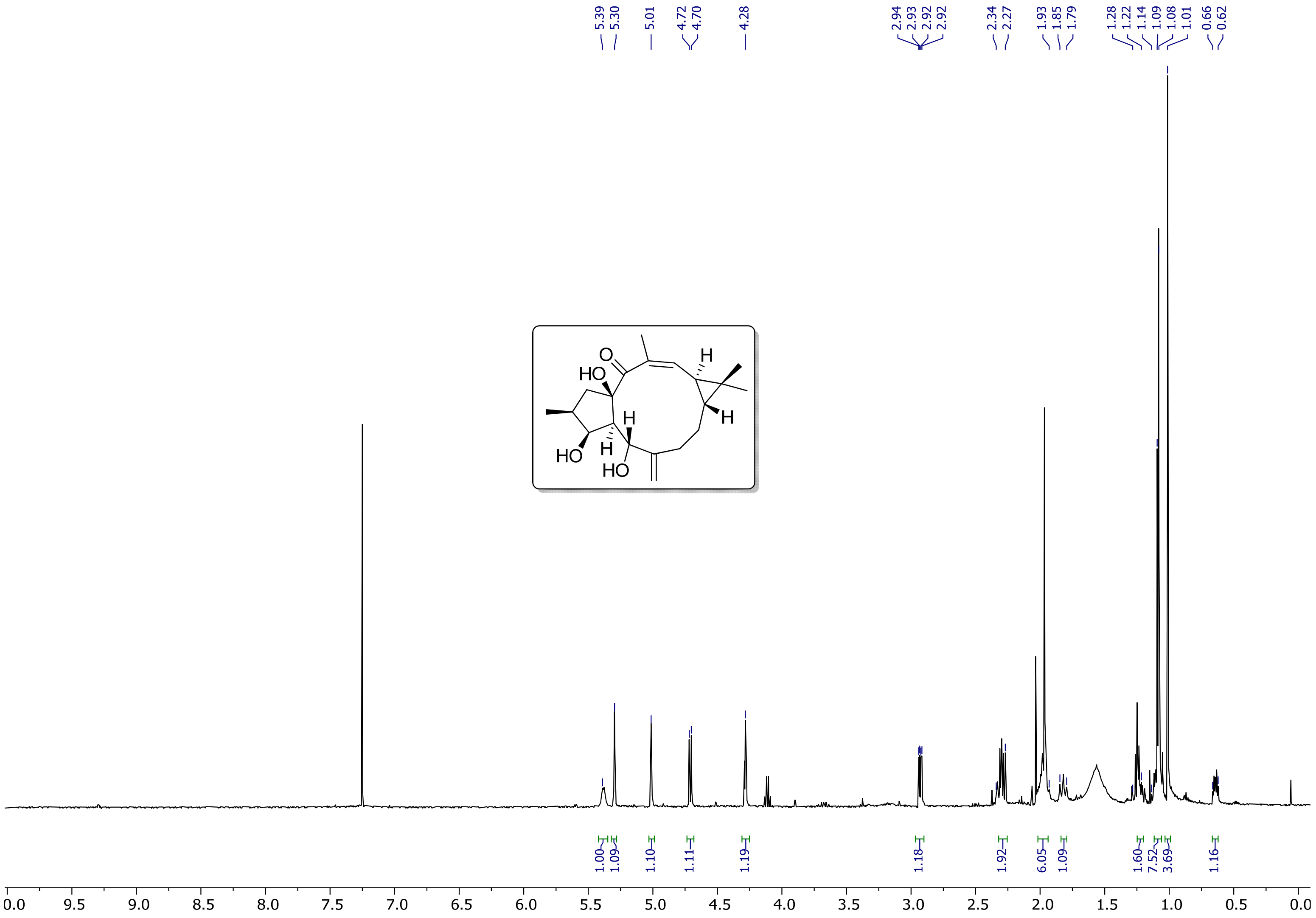

Figure S33. ^{1}H NMR spectrum (500 MHz) of compound **5a** in $CDCl_3$.

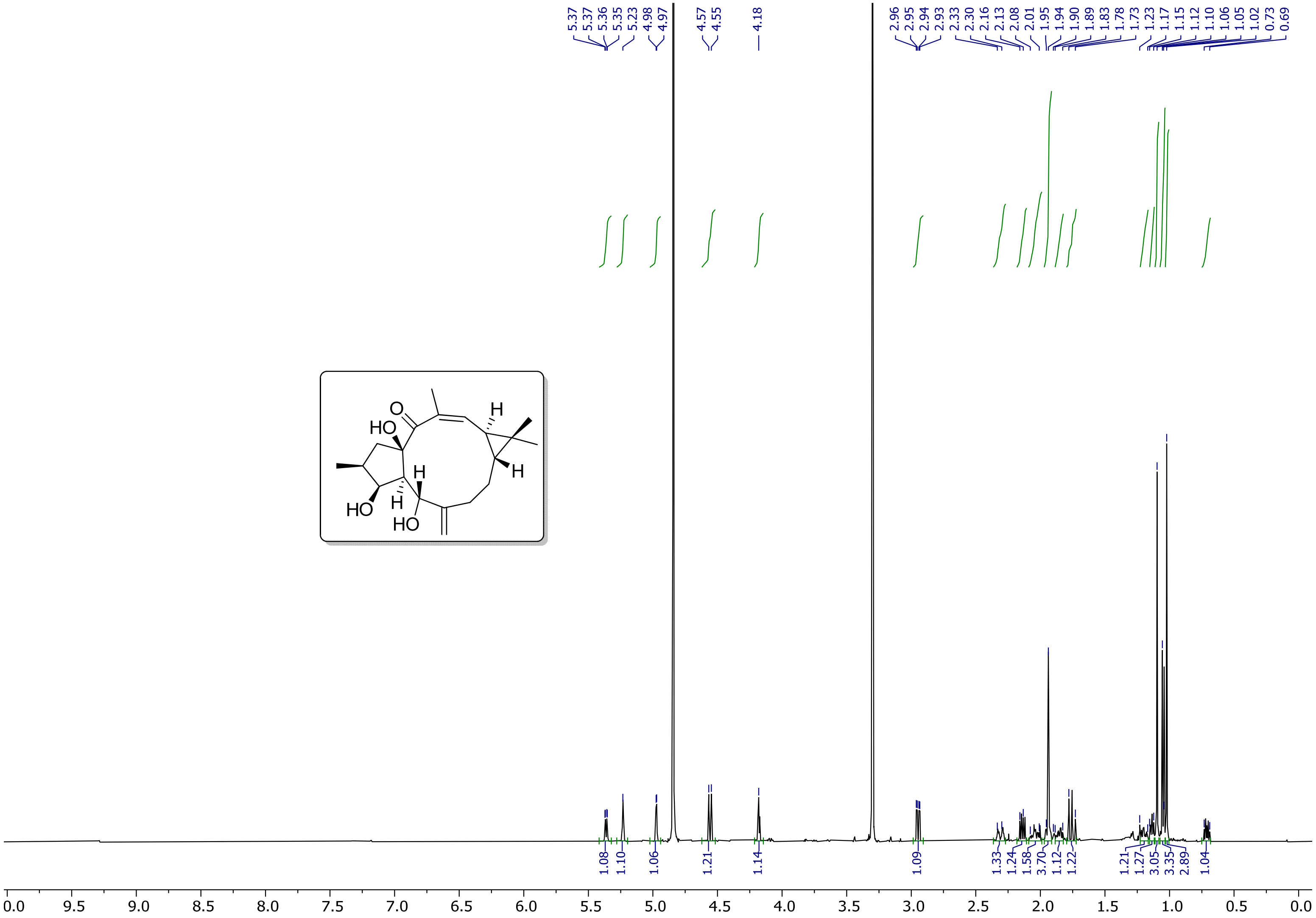

Figure S34. ^{1}H NMR spectrum (500 MHz) of compound **5a** in CD_3OD.

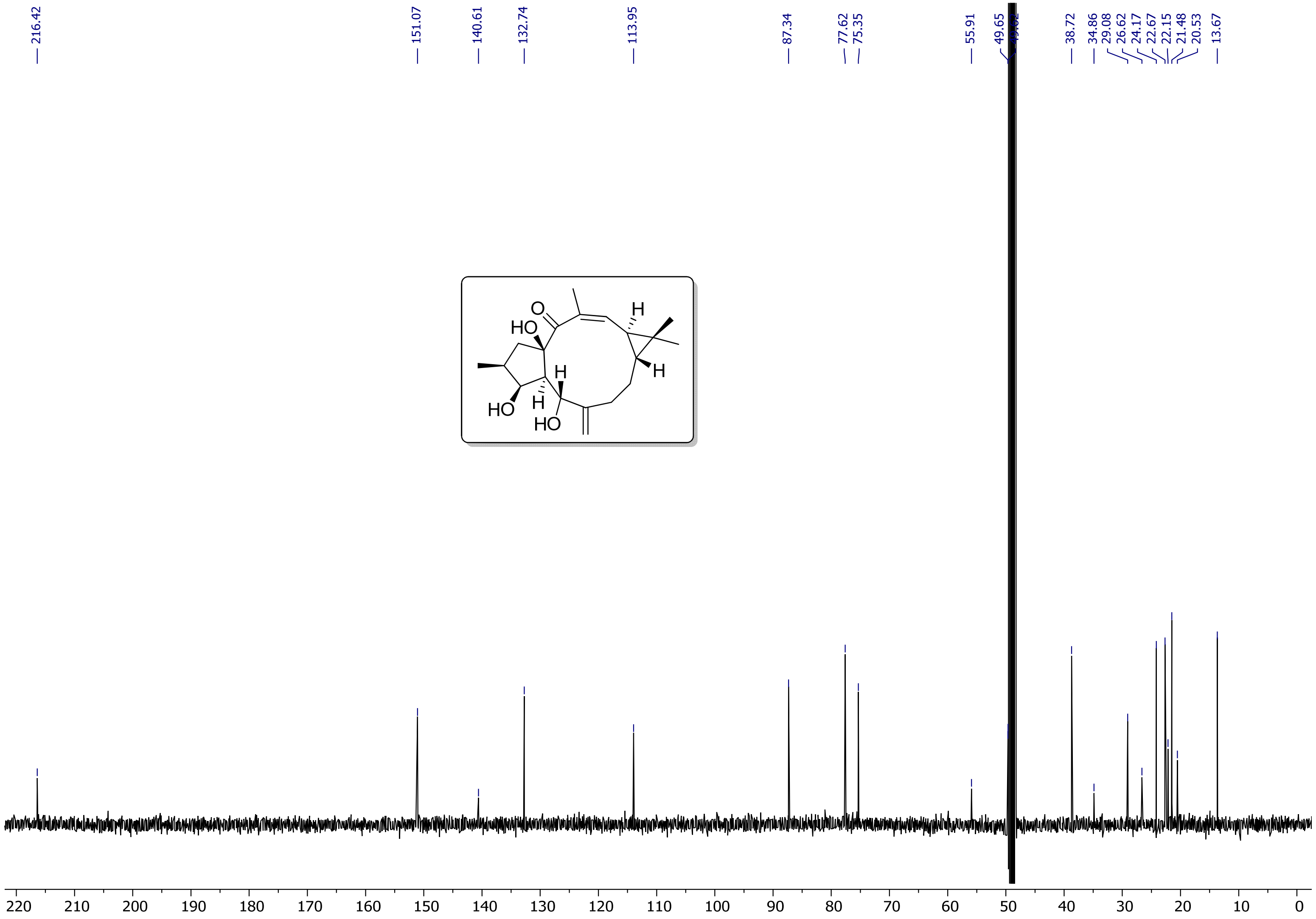

Figure S35. ^{13}C NMR spectrum (125 MHz) of compound **5a** in CD_3OD.

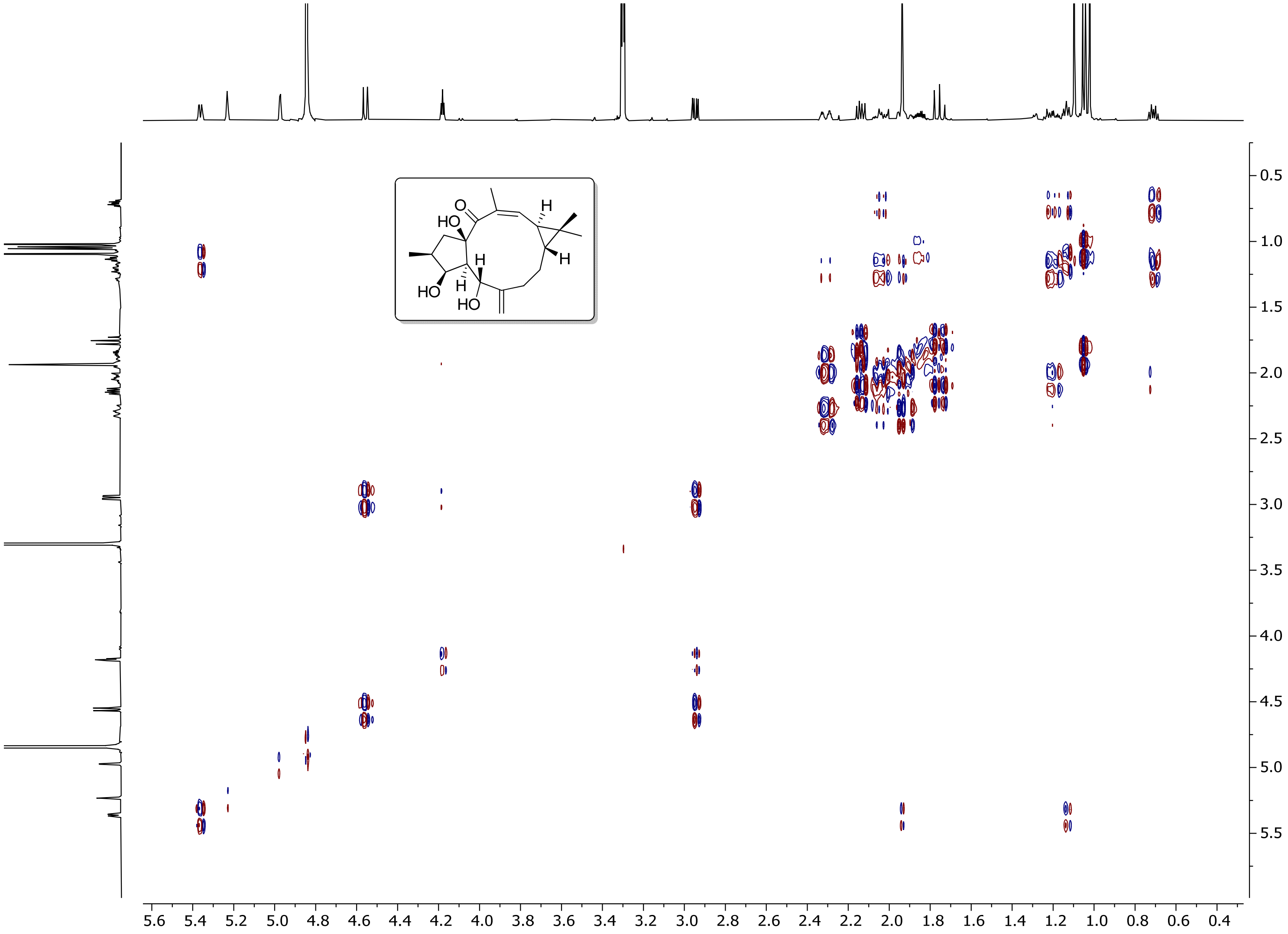

Figure S36. gCOSY spectrum of compound **5a** in CD_3OD.

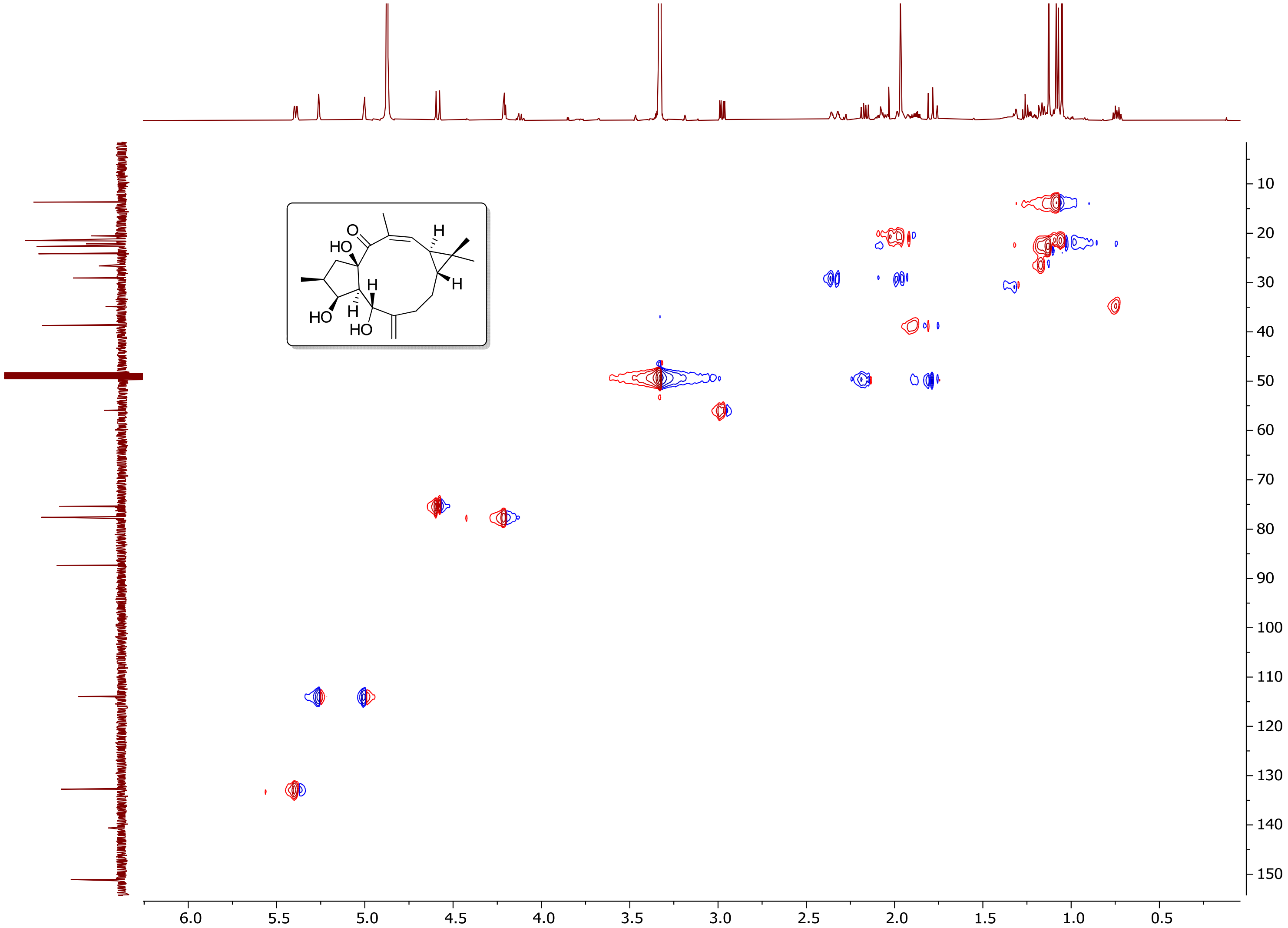

Figure S37. gHSQC spectrum of compound **5a** in CD_3OD.

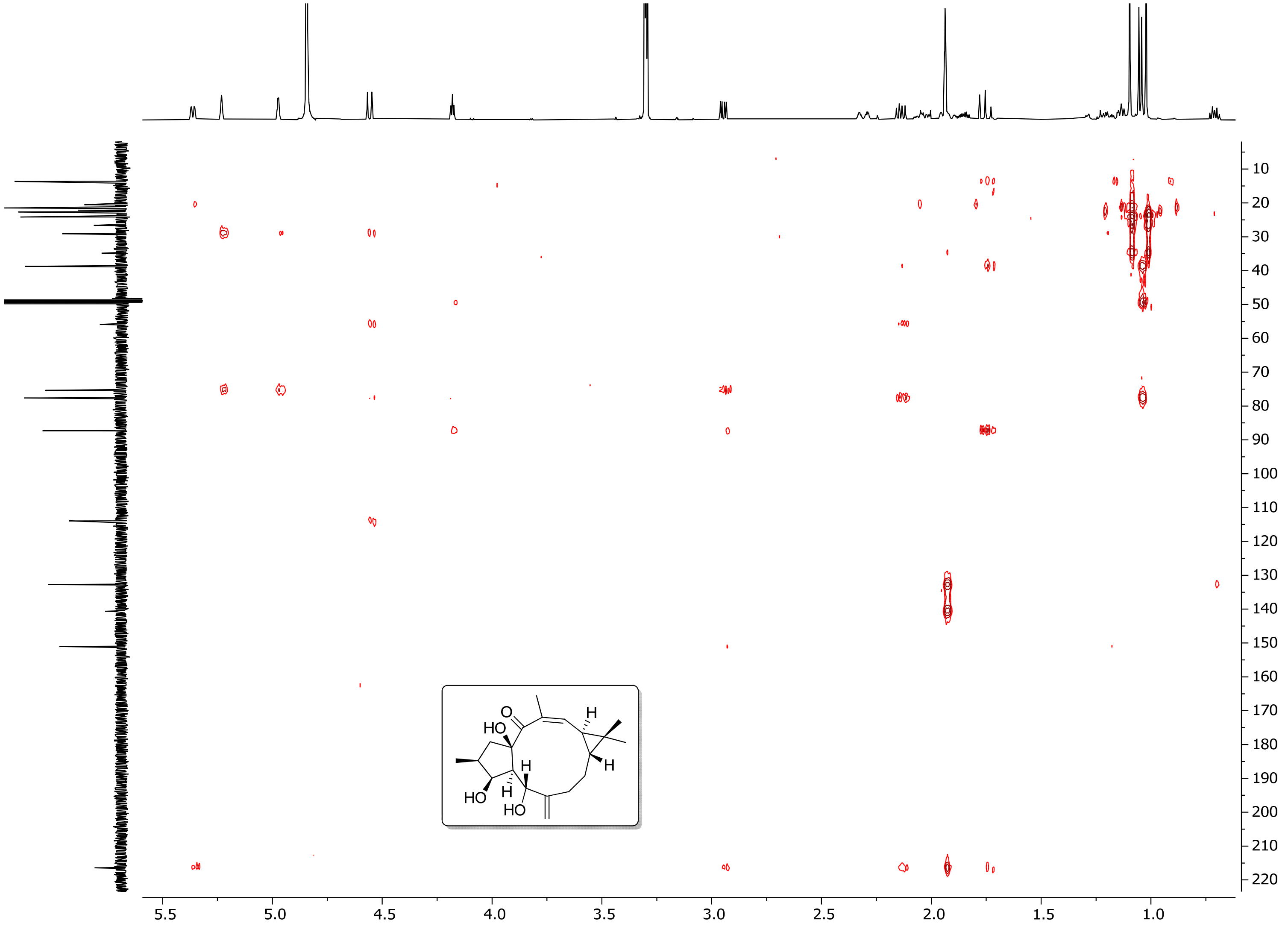

Figure S38. gHMBC spectrum of compound **5a** in CD_3OD.

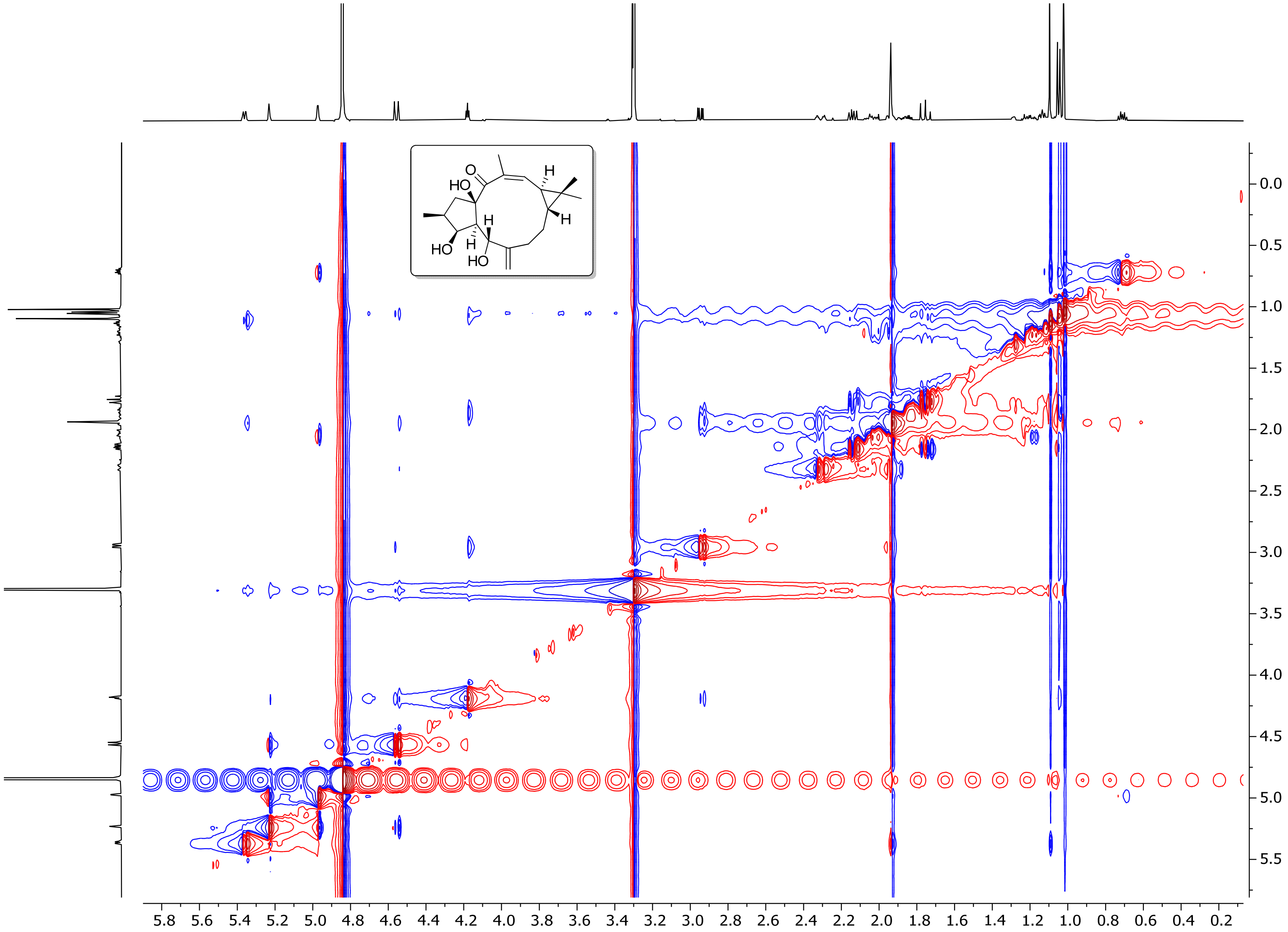

Figure S39. 2D NOESY spectrum of compound **5a** in CD_3OD.

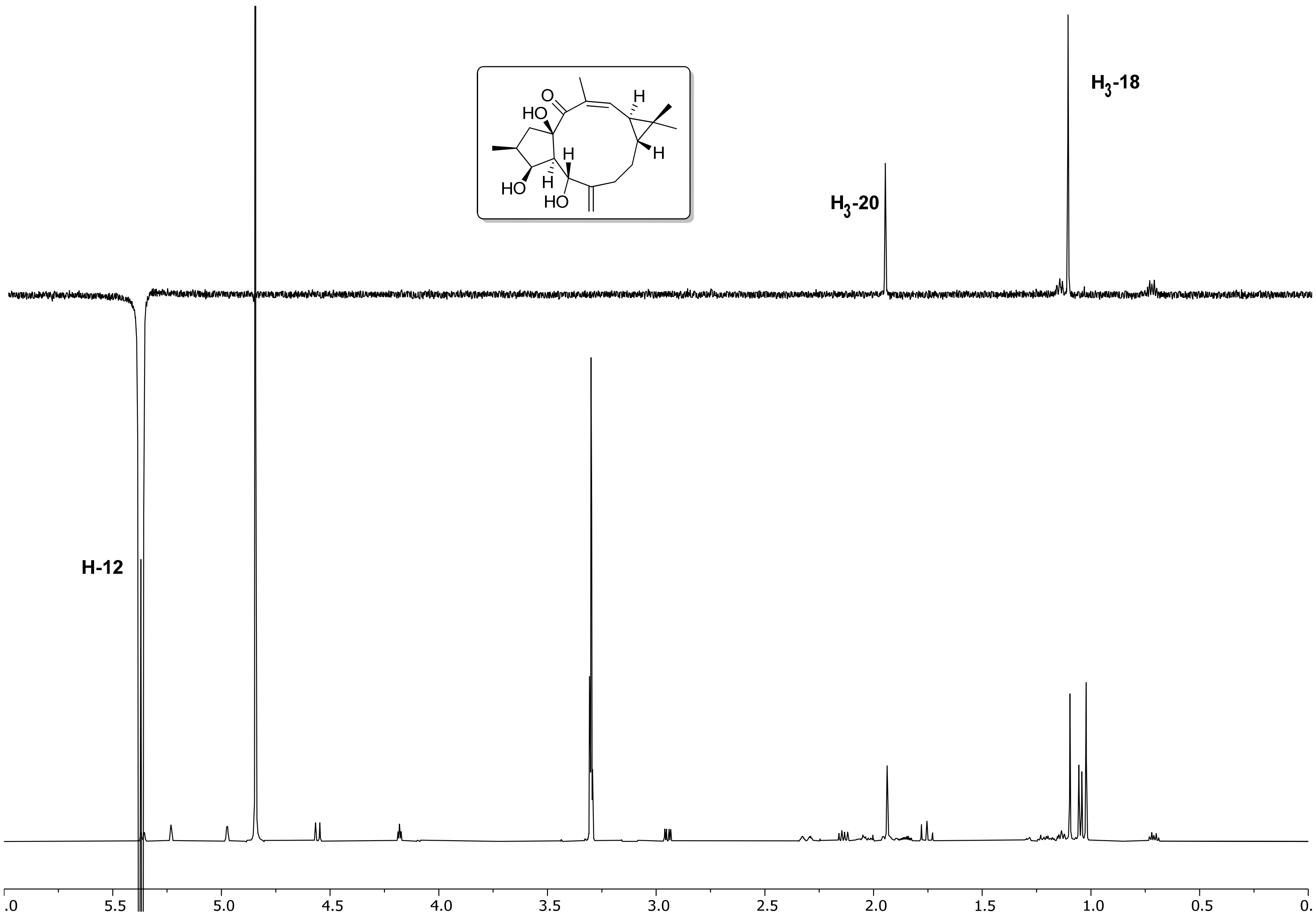

Figure S40a. 1D NOESY spectrum of compound **5a** in CD_3OD.

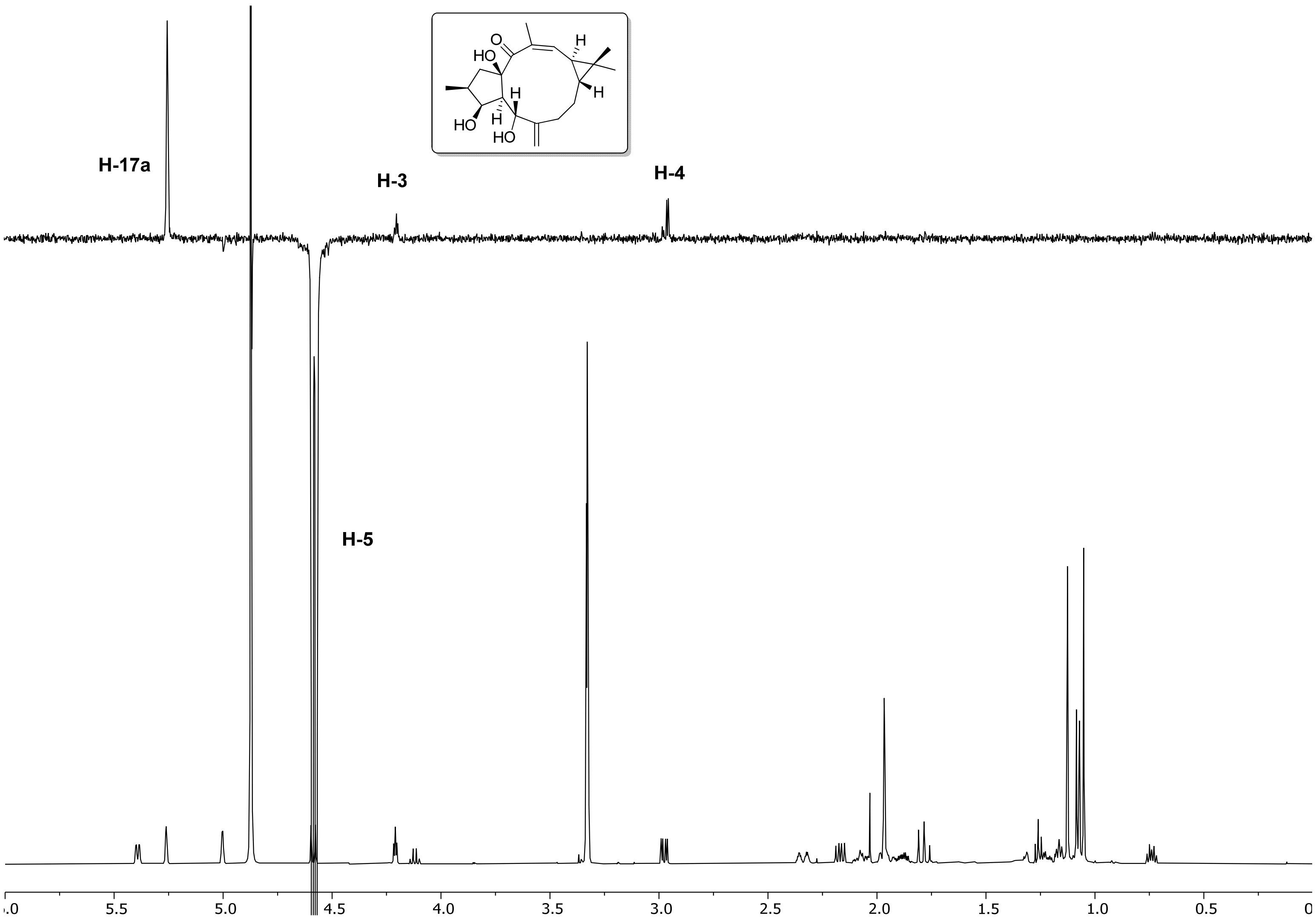

Figure S40b. 1D NOESY spectrum of compound **5a** in CD_3OD.

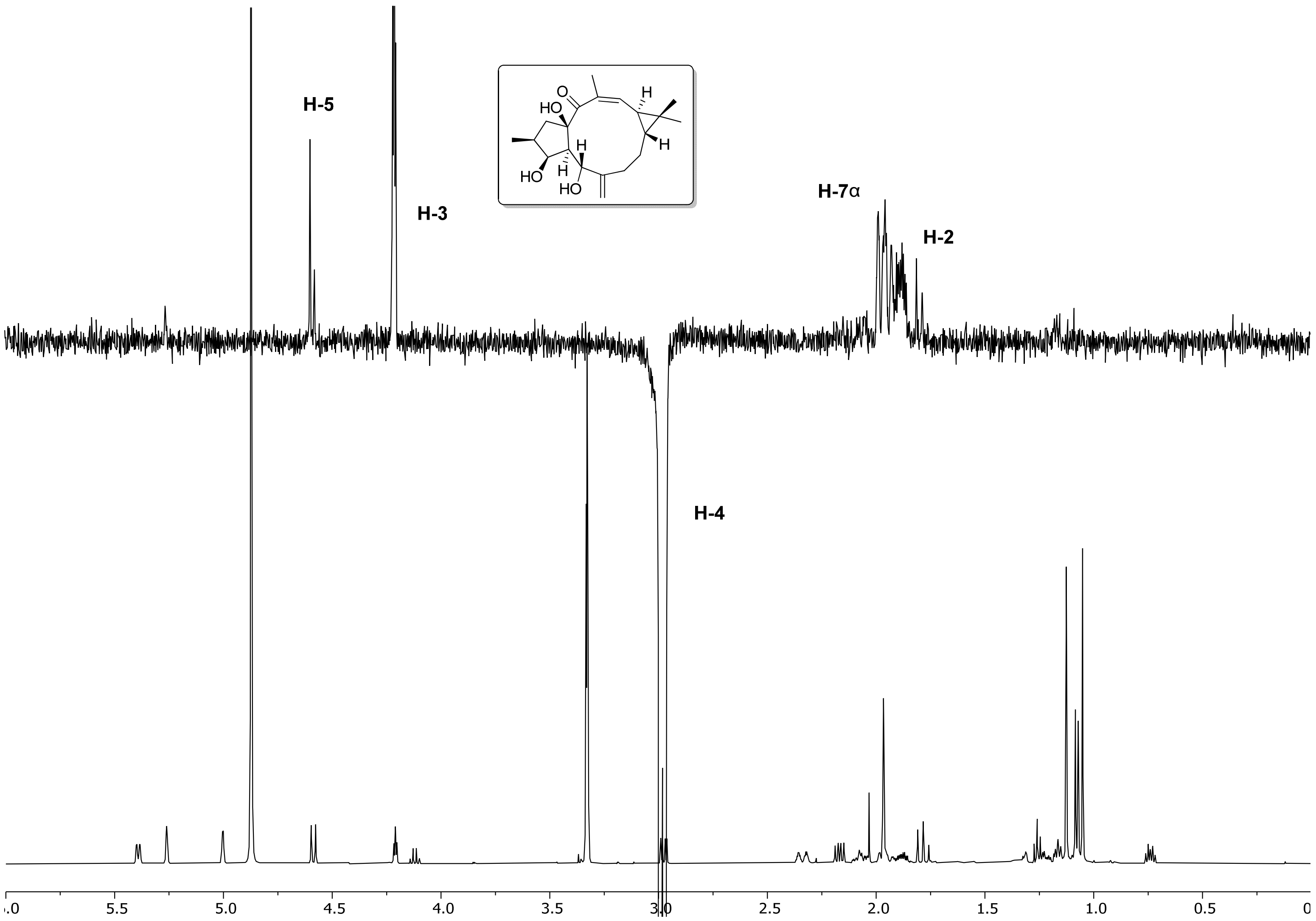

Figure S40c. 1D NOESY spectrum of compound **5a** in CD_3OD.

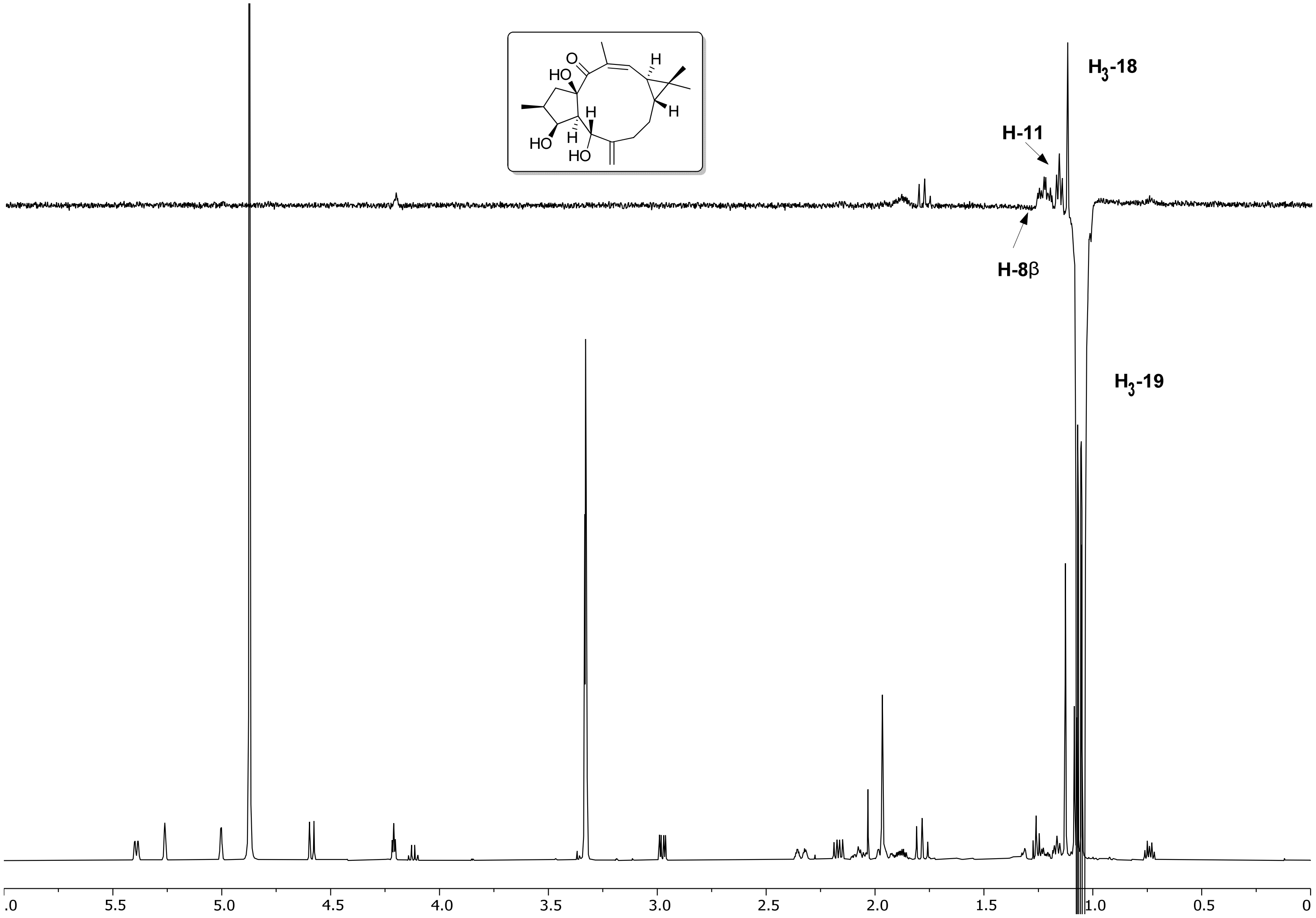

Figure S40d. 1D NOESY spectrum of compound **5a** in CD_3OD.

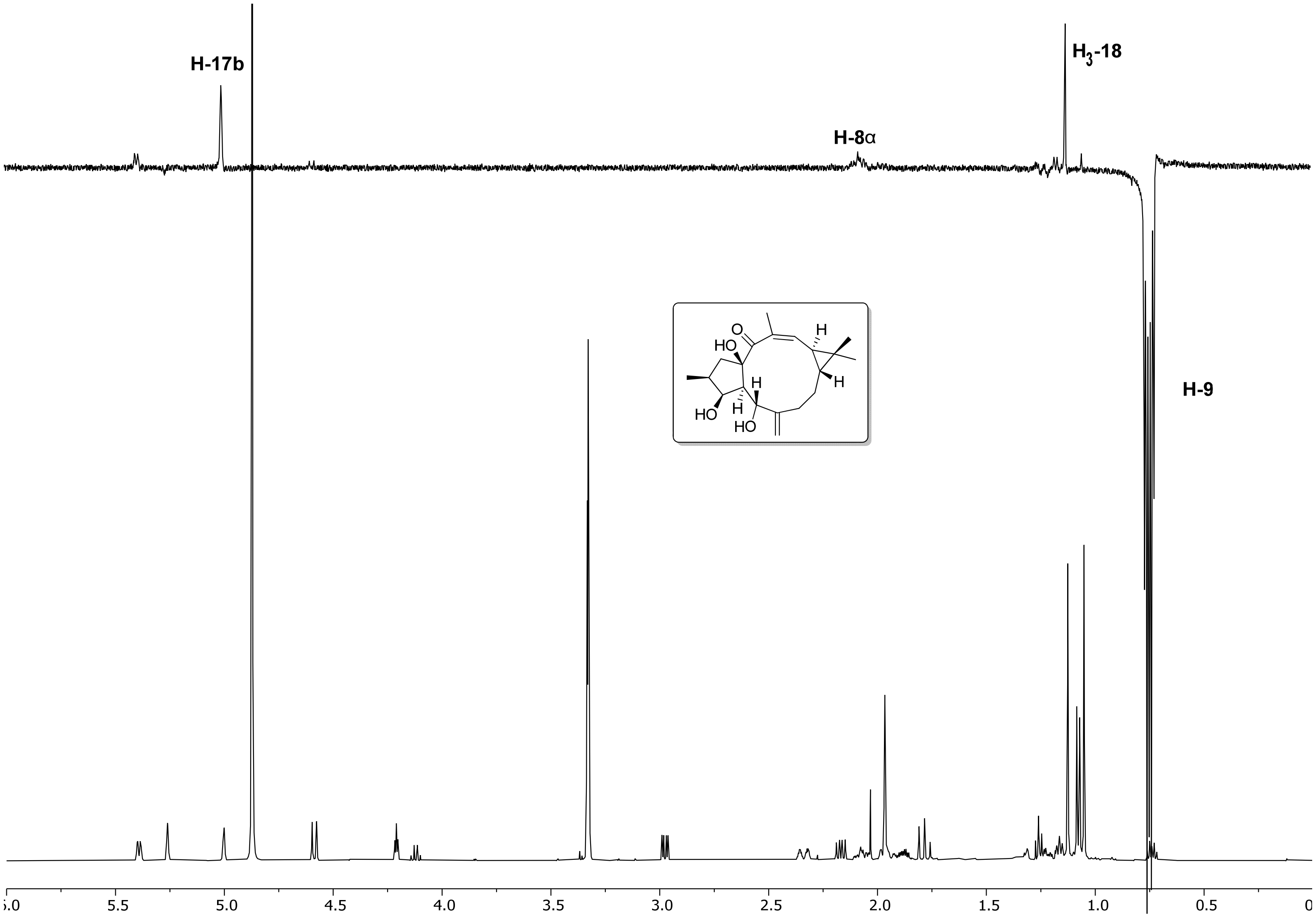

Figure S40e. 1D NOESY spectrum of compound **5a** in CD_3OD.

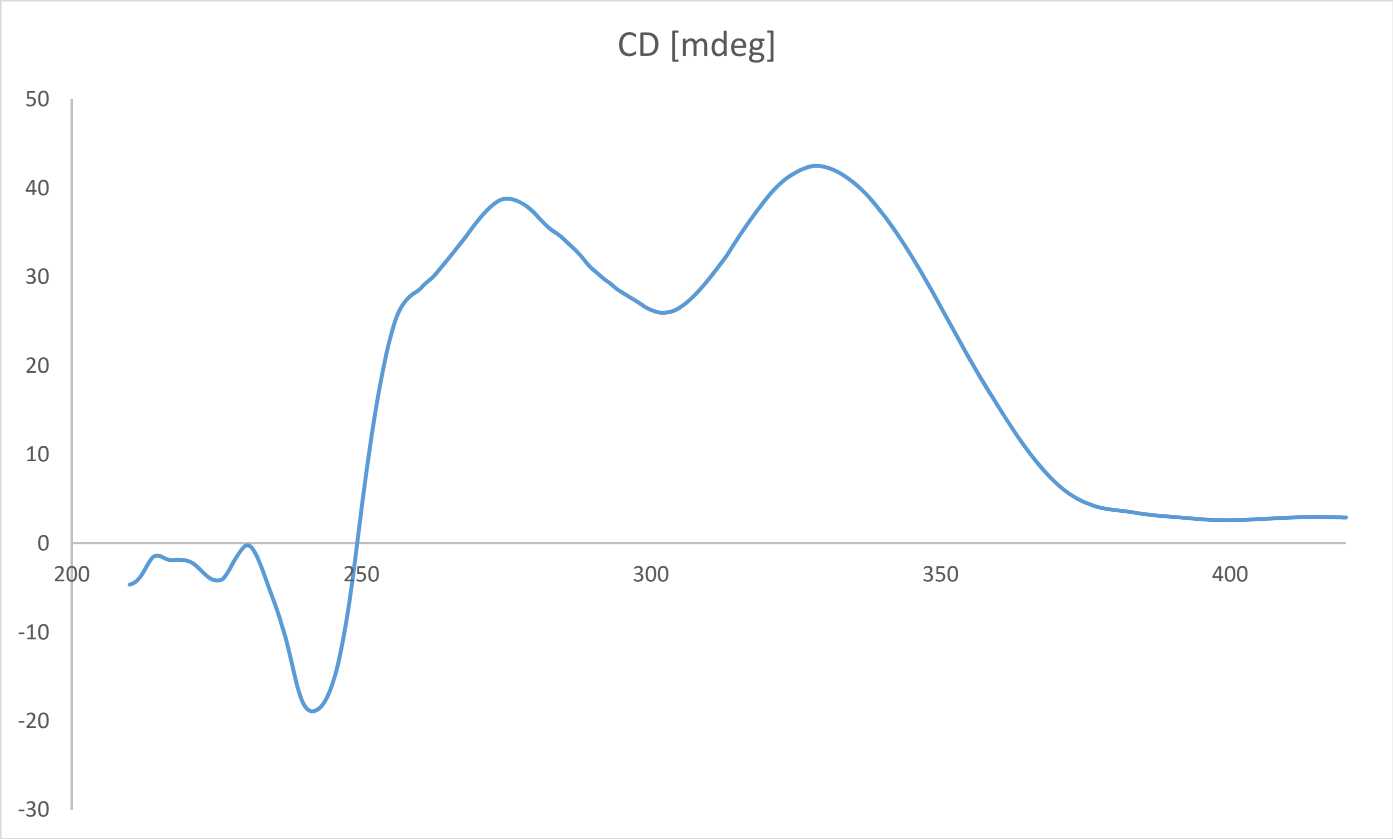

Figure S41. ECD spectrum of compound **5a**.

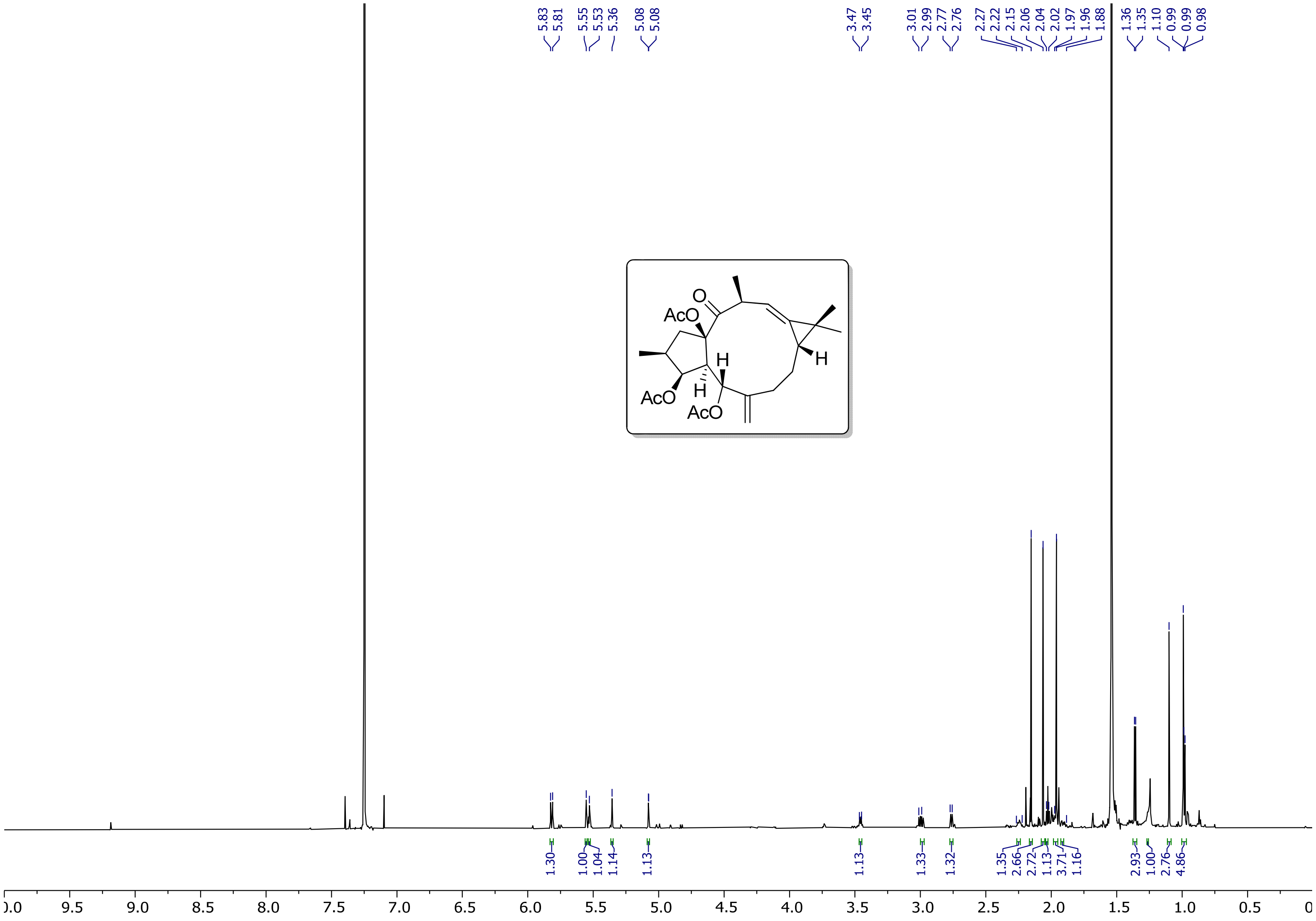

Figure S42. ^{1}H NMR spectrum (700 MHz) of compound **6** in $CDCl_3$.

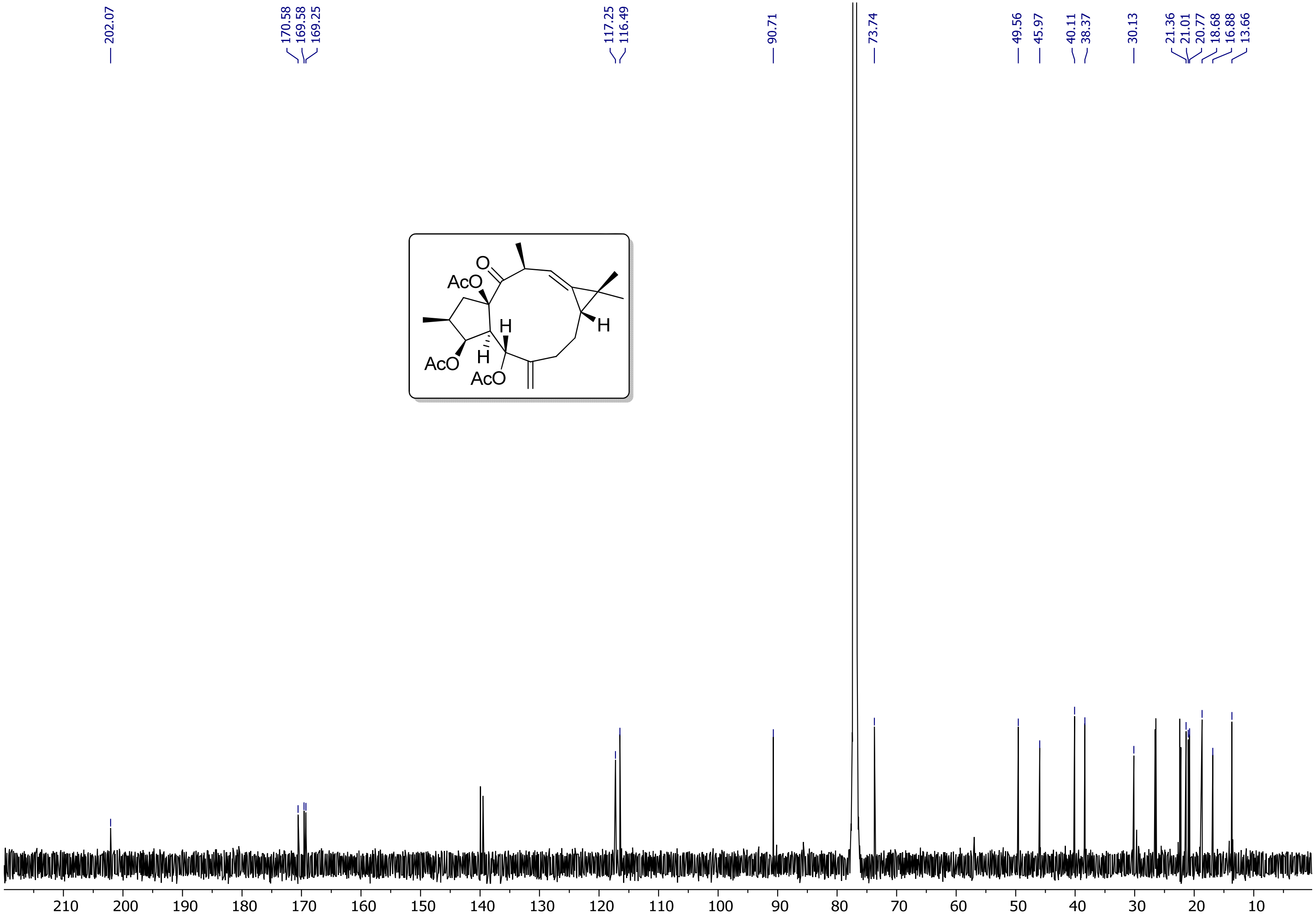

Figure S43. ^{13}C NMR spectrum (175 MHz) of compound **6** in $CDCl_3$.

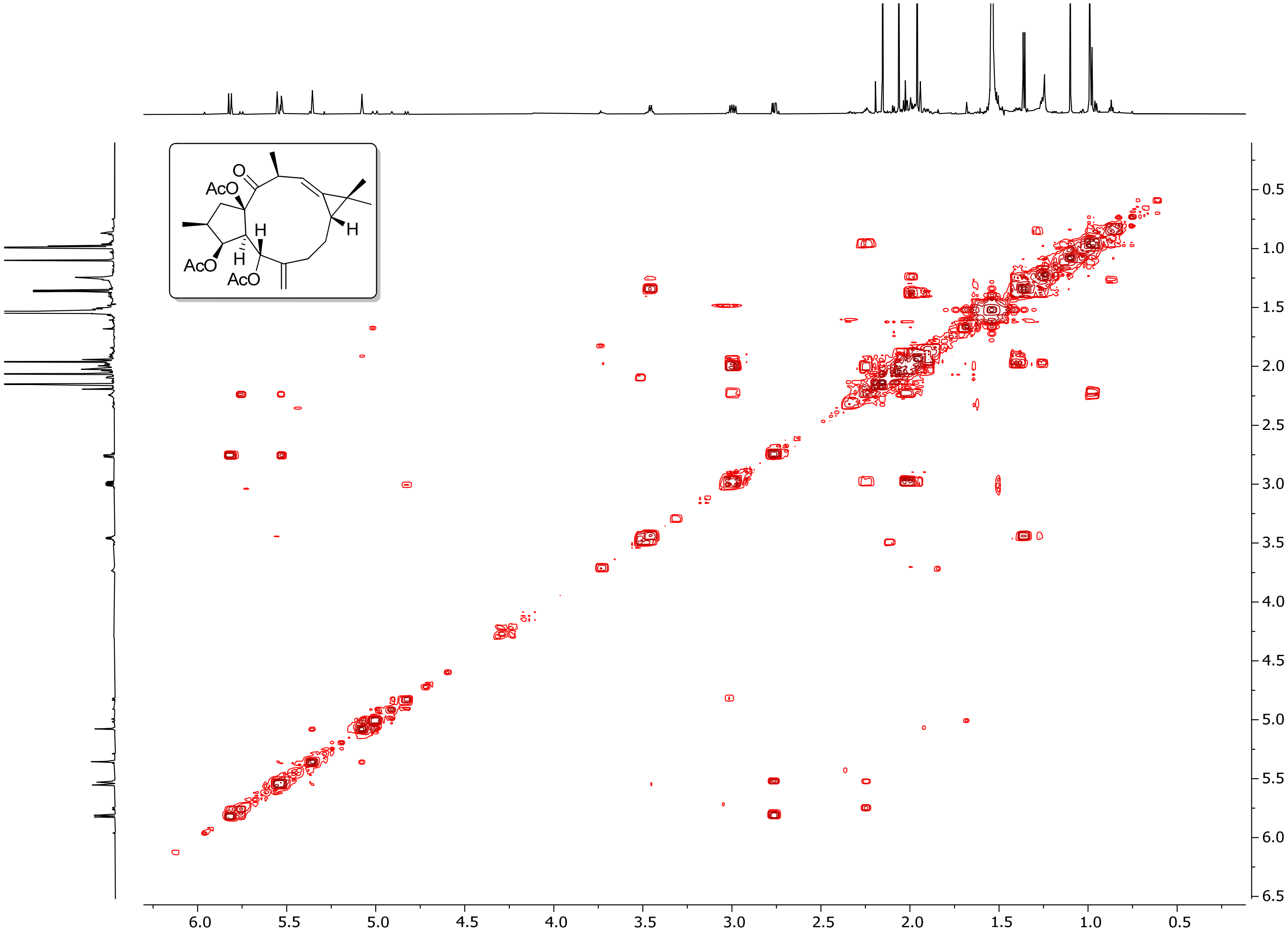

Figure S44. gCOSY spectrum of compound **6**.

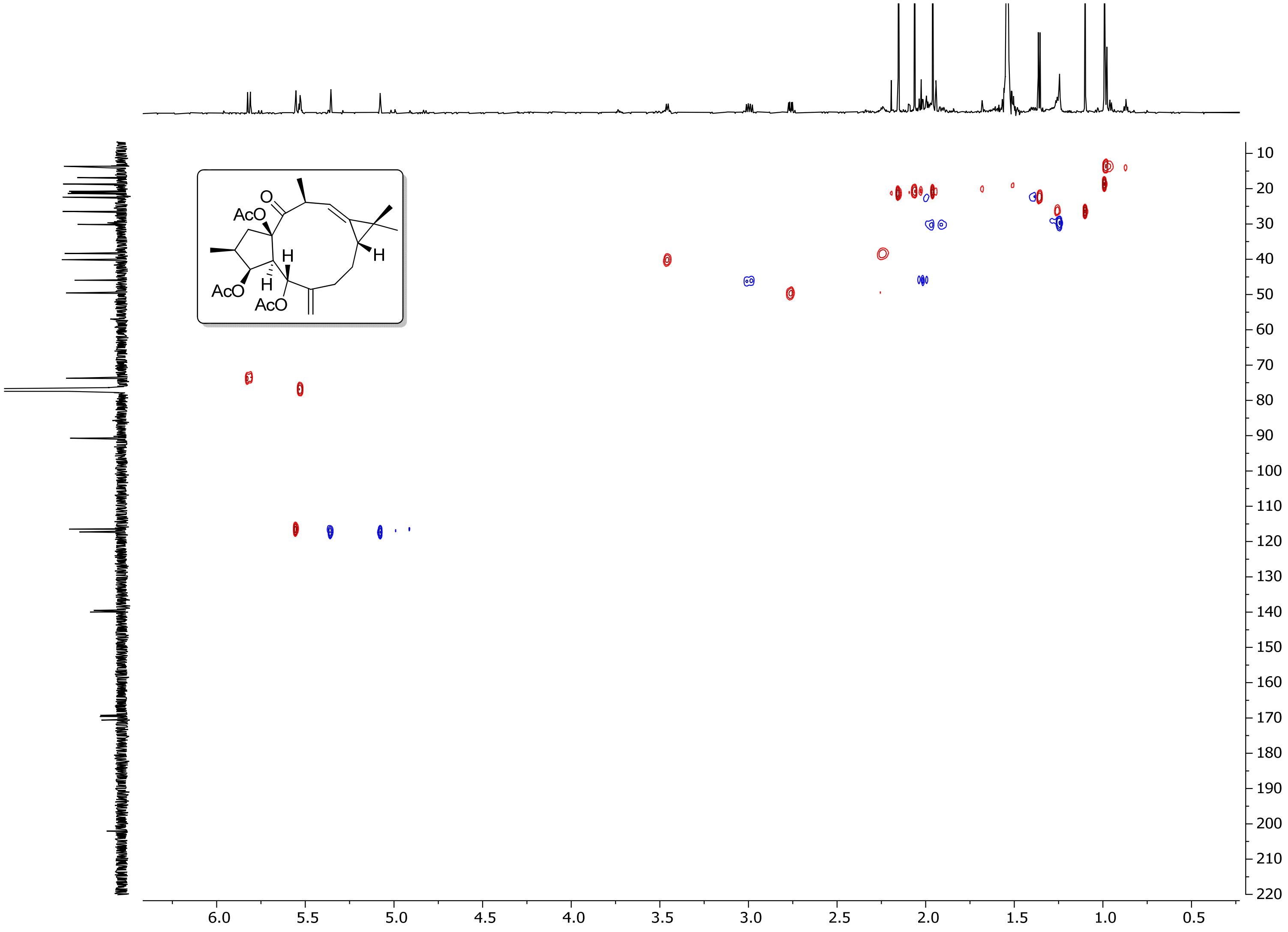

Figure S45. gHSQC spectrum of compound **6**.

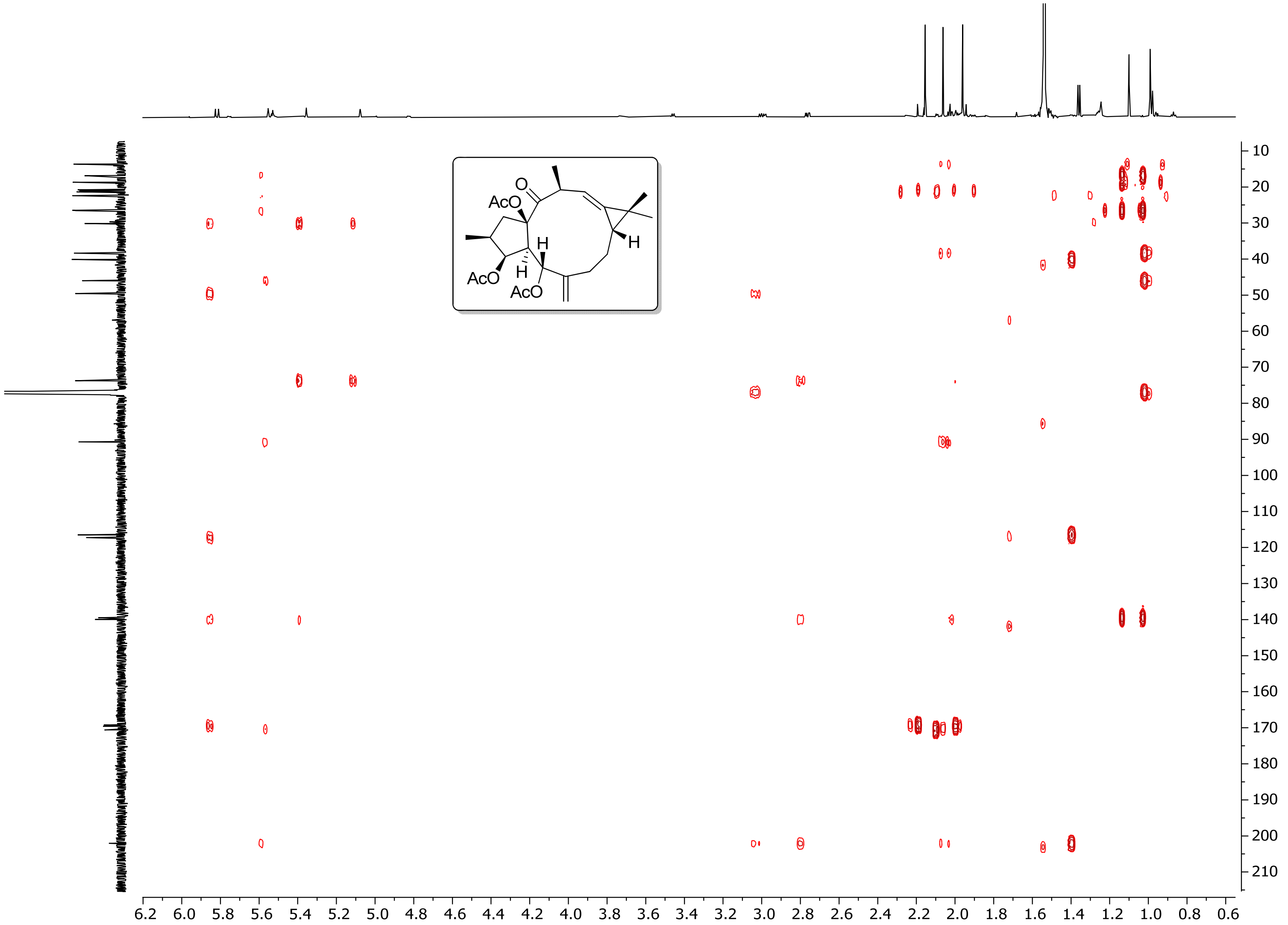

Figure S46. gHMBC spectrum of compound **6**.

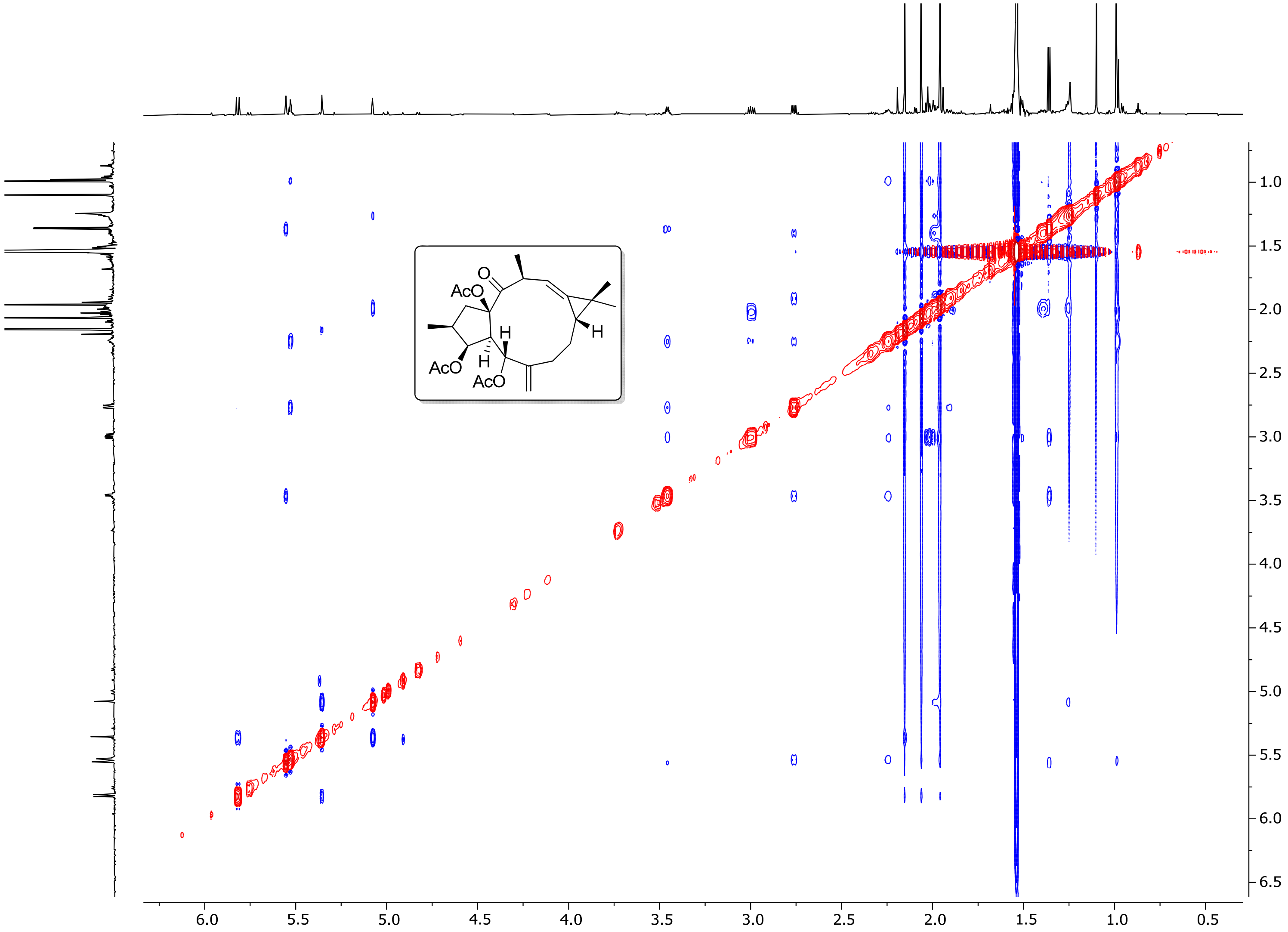

Figure S47. 2D NOESY spectrum of compound **6**.

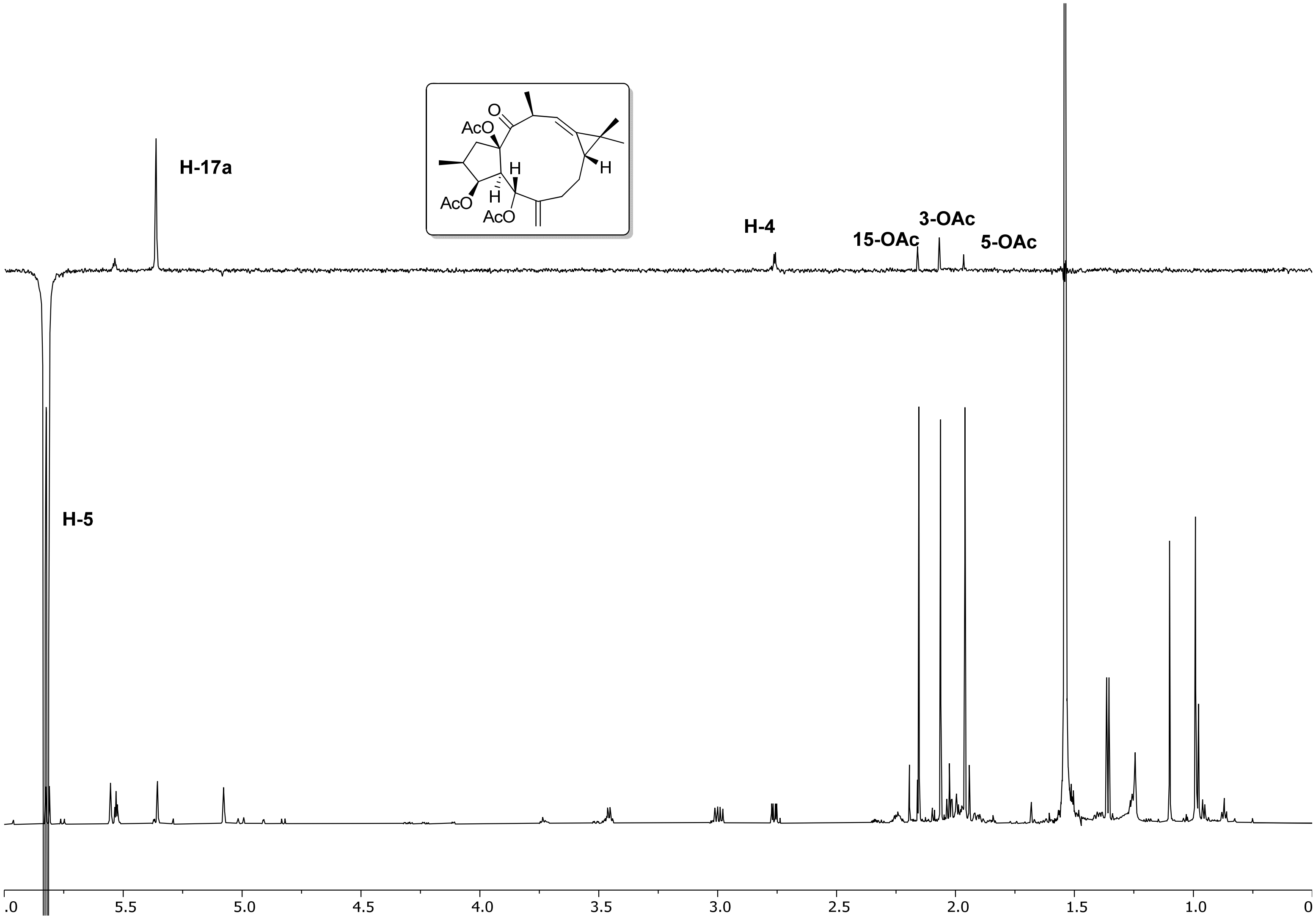
H-17a
H-4
15-OAc
3-OAc
5-OAc
H-5
AcO
O
AcO
H
H
H
AcO
.0
5.5
5.0
4.5
4.0
3.5
3.0
2.5
2.0
1.5
1.0
0

Figure S48a. 1D NOESY spectrum of compound **6**.

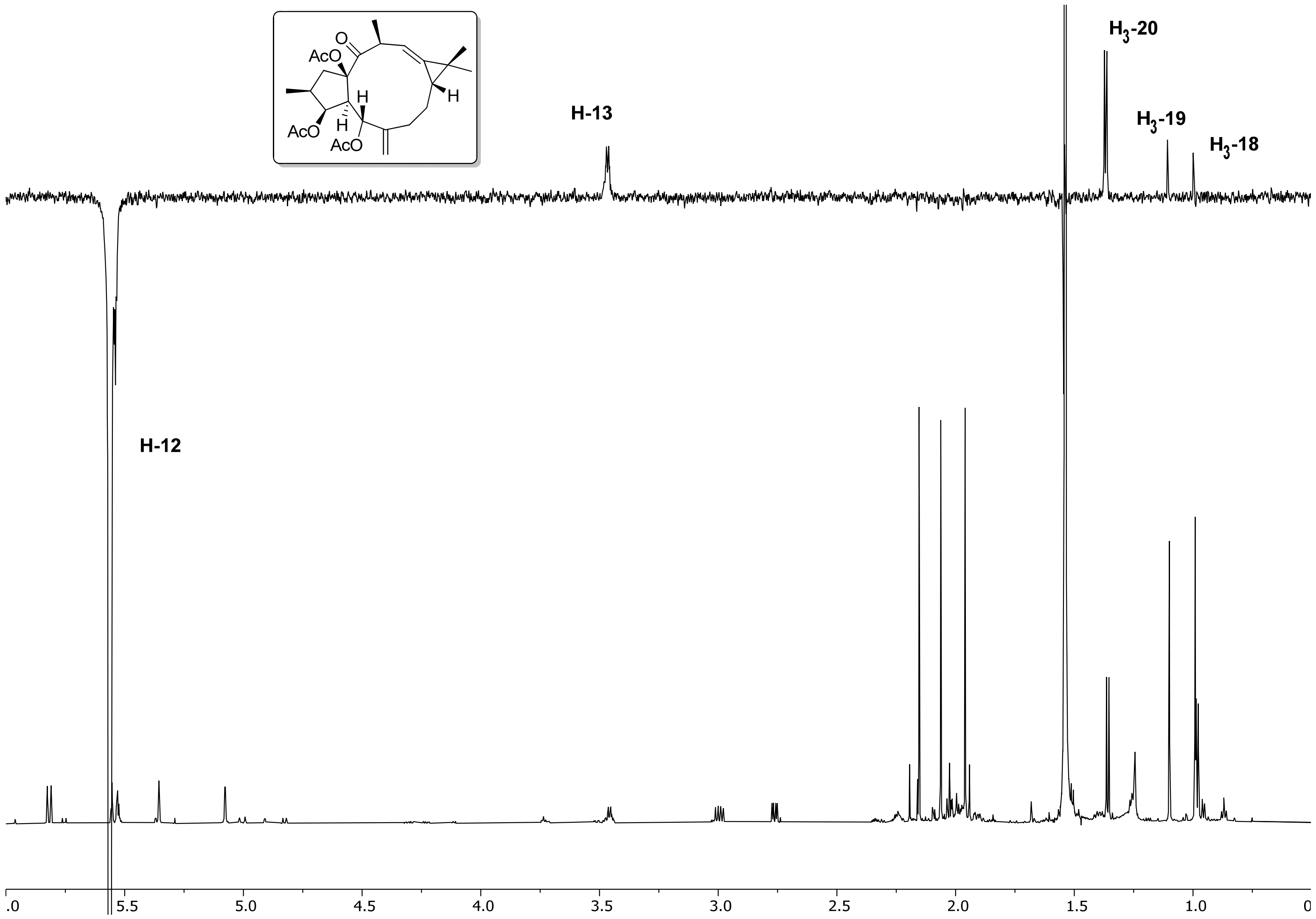

Figure S48b. 1D NOESY spectrum of compound **6**.

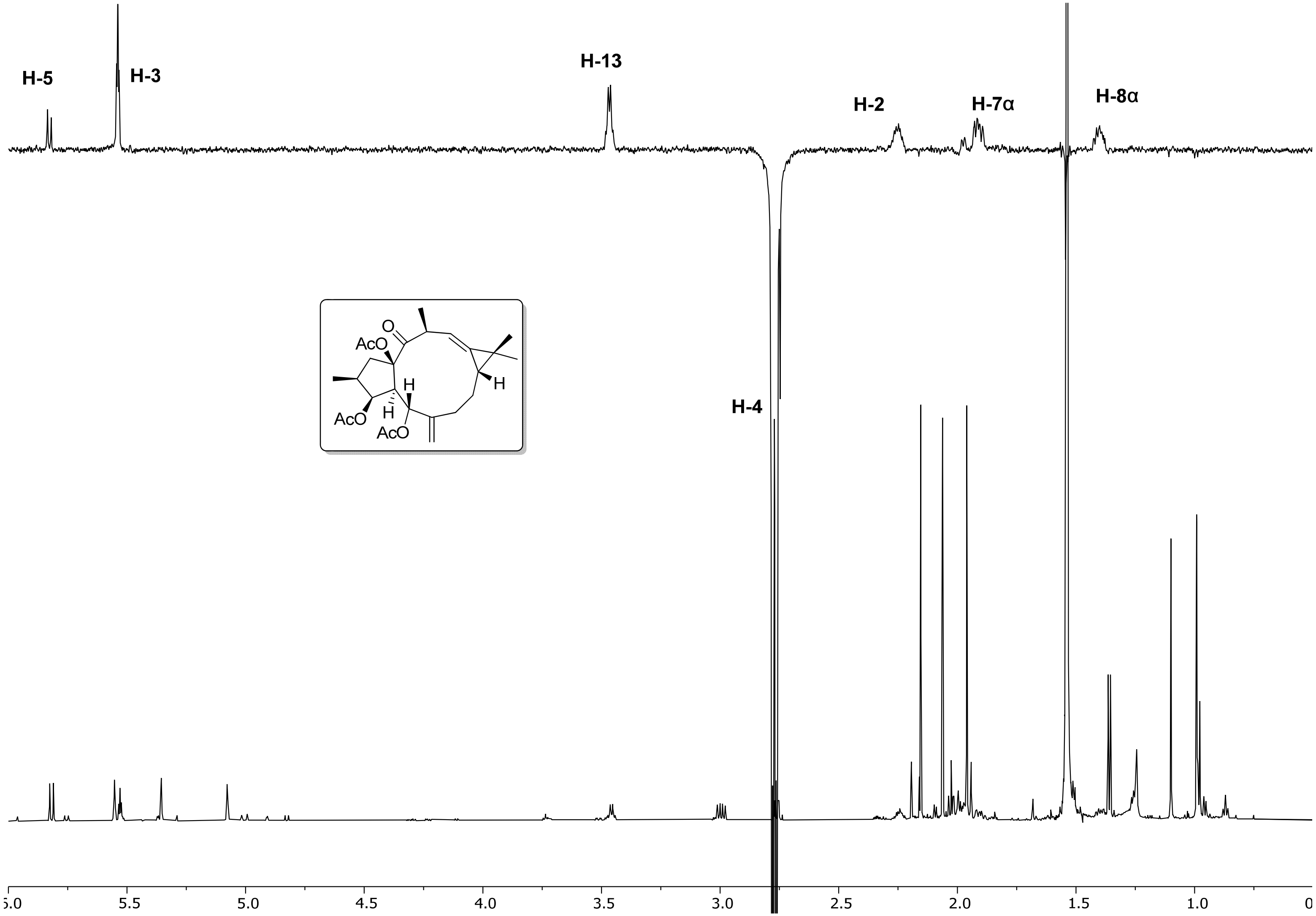

Figure S48c. 1D NOESY spectrum of compound **6**.

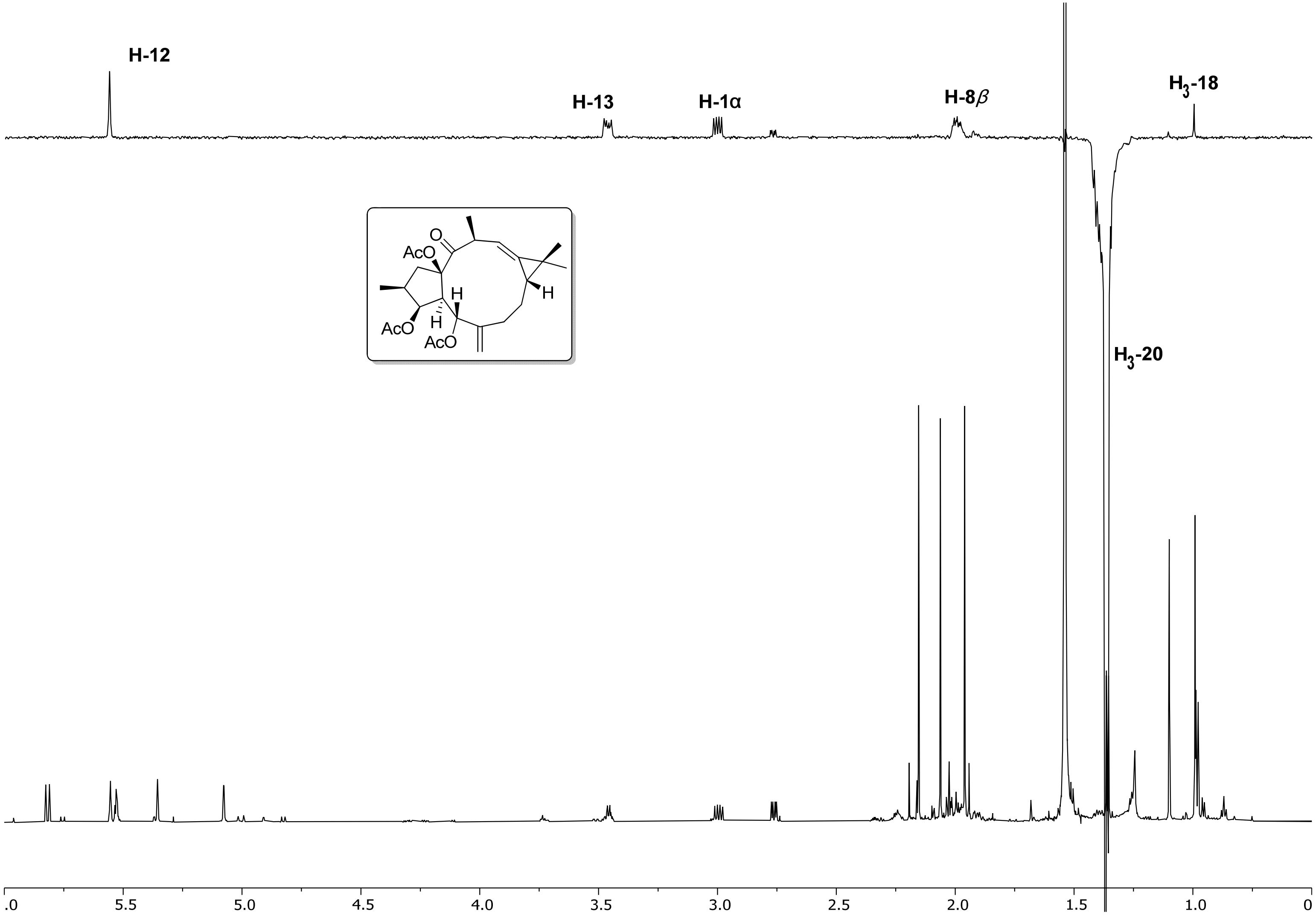

Figure S48d. 1D NOESY spectrum of compound **6**.

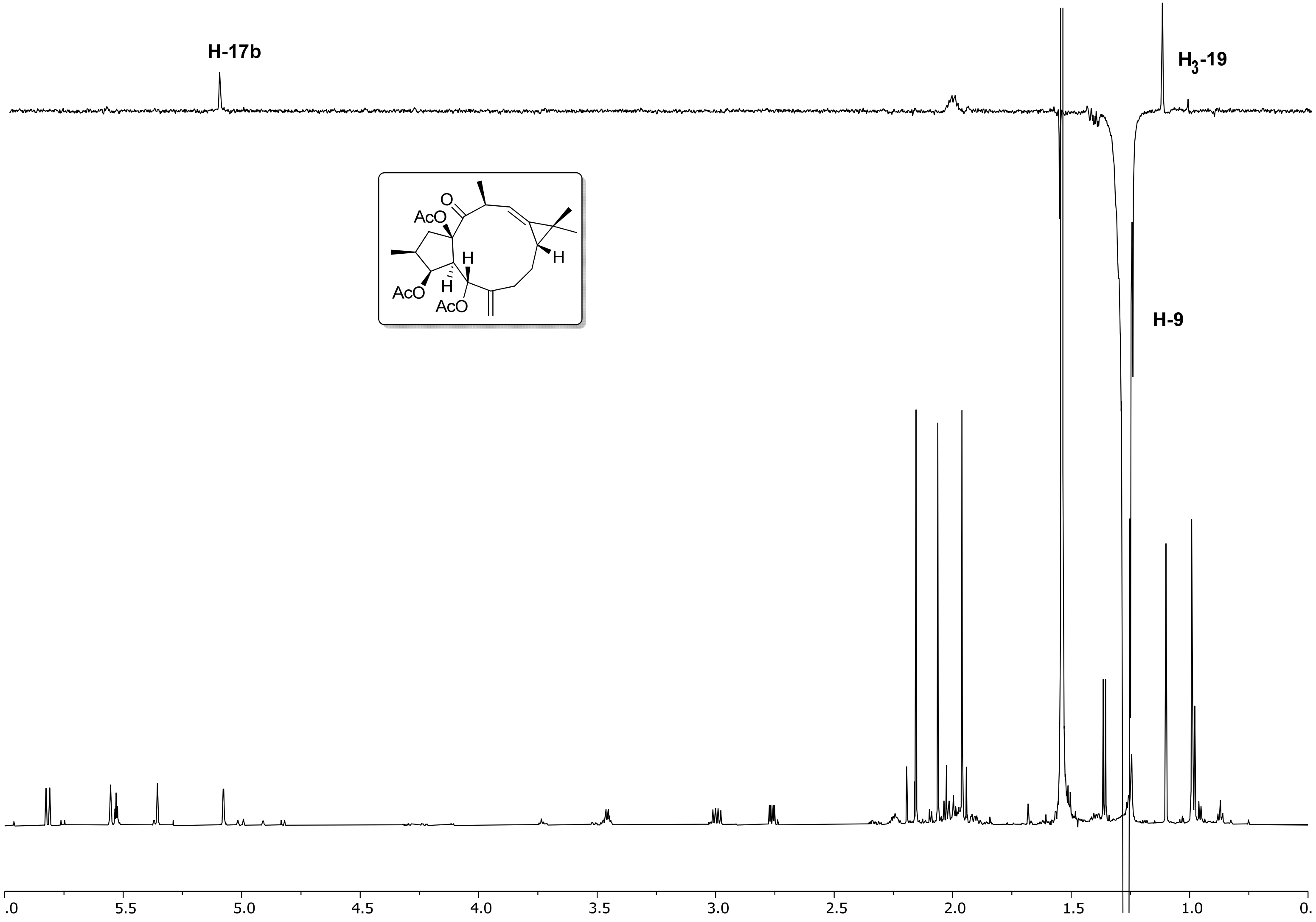

Figure S48e. 1D NOESY spectrum of compound **6**.

Elemental Composition Report

Page 1

Single Mass Analysis

Tolerance = 5.0 mDa / DBE: min = -1.5, max = 50.0
Element prediction: Off
Number of isotope peaks used for i-FIT = 3

Monoisotopic Mass, Even Electron Ions
233 formula(e) evaluated with 3 results within limits (up to 50 closest results for each mass)
Elements Used:
C: 0-300 H: 0-100 O: 0-200 23Na: 0-1
HPLC17--MSe2pos 287 (2.324)
2: TOF MS ES+

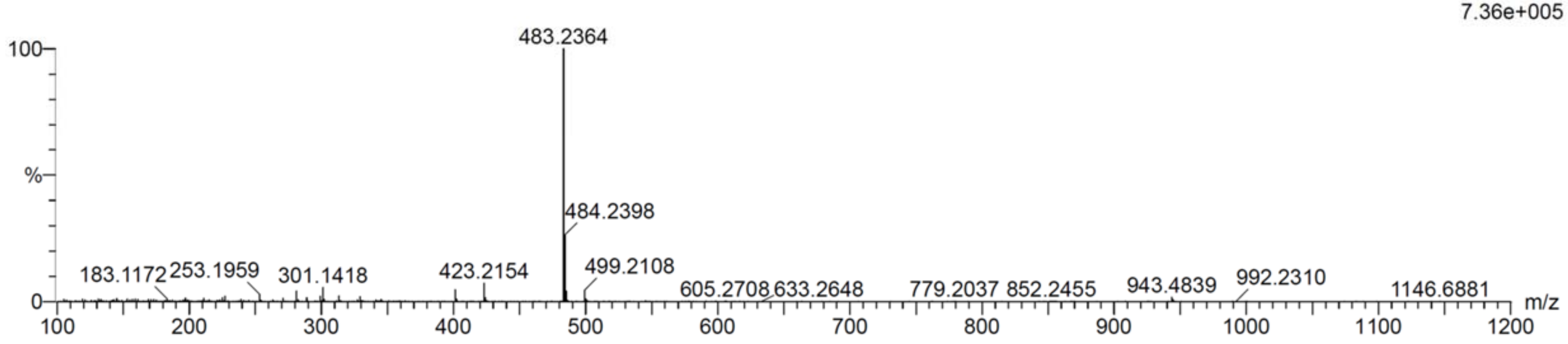

Minimum:				-1.5				
Maximum:		5.0	10.0	50.0				

Mass	Calc. Mass	mDa	PPM	DBE	i-FIT	Norm	Conf(%)	Formula
483.2364	483.2359	0.5	1.0	8.5	794.9	0.076	92.72	C26 H36 O7 23Na
	483.2383	-1.9	-3.9	11.5	797.4	2.628	7.23	C28 H35 O7
	483.2324	4.0	8.3	20.5	802.3	7.491	0.06	C35 H31 O2

Figure S49. HRMS spectrum of compound **6**.

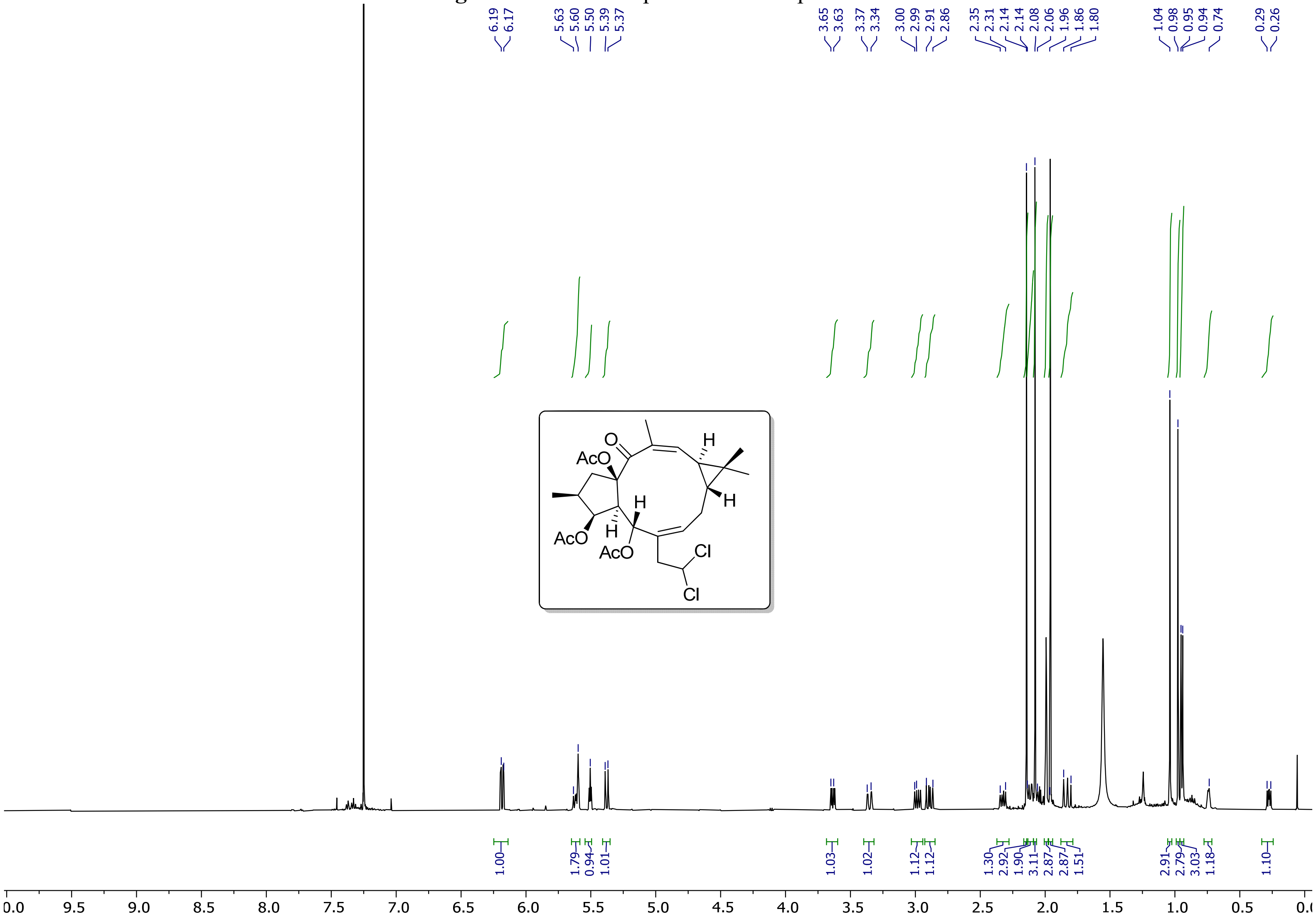

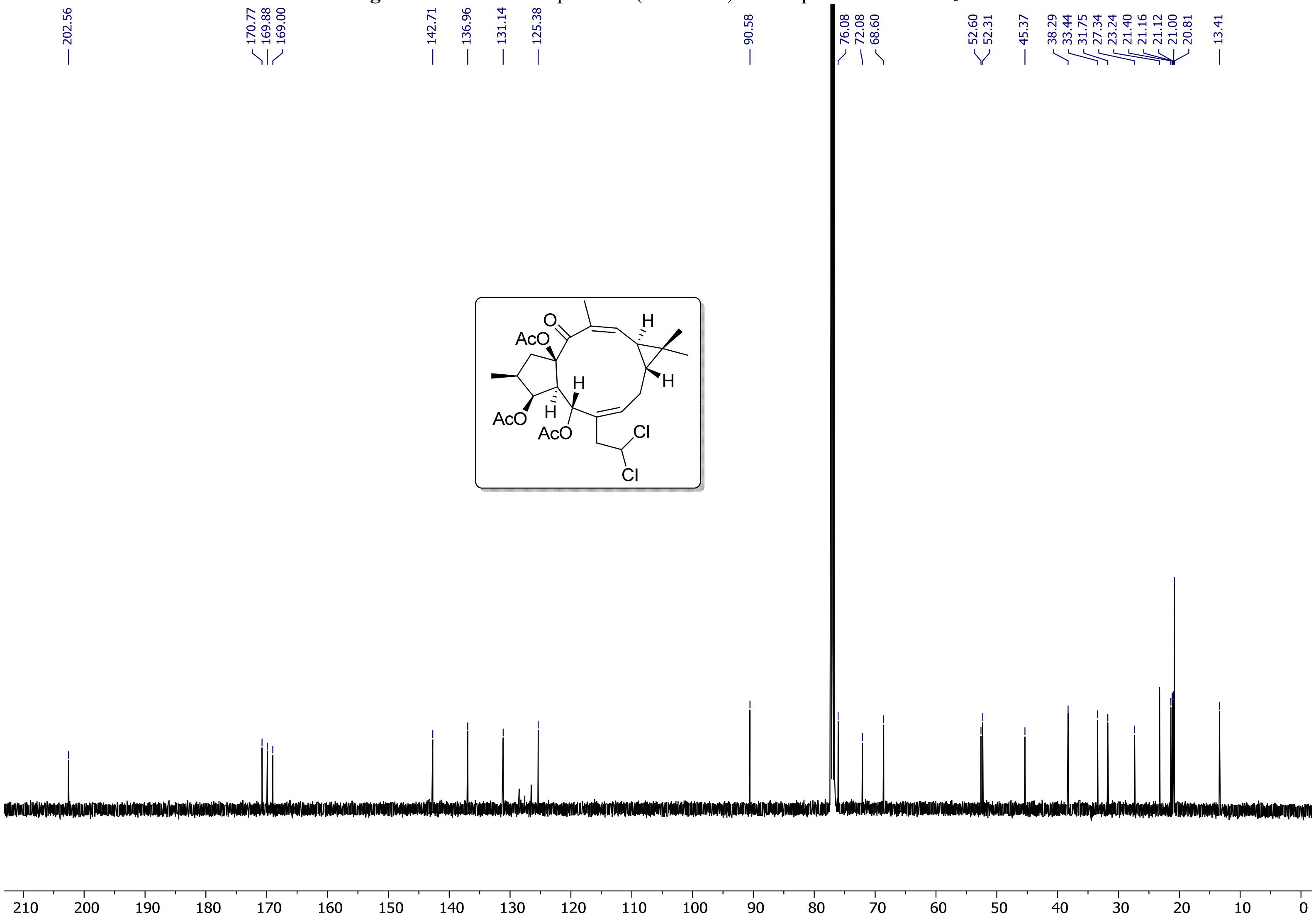

Figure S50. ^{1}H NMR spectrum (500 MHz) of compound **7** in $CDCl_3$.

Figure S51. ^{13}C NMR spectrum (125 MHz) of compound **7** in $CDCl_3$.

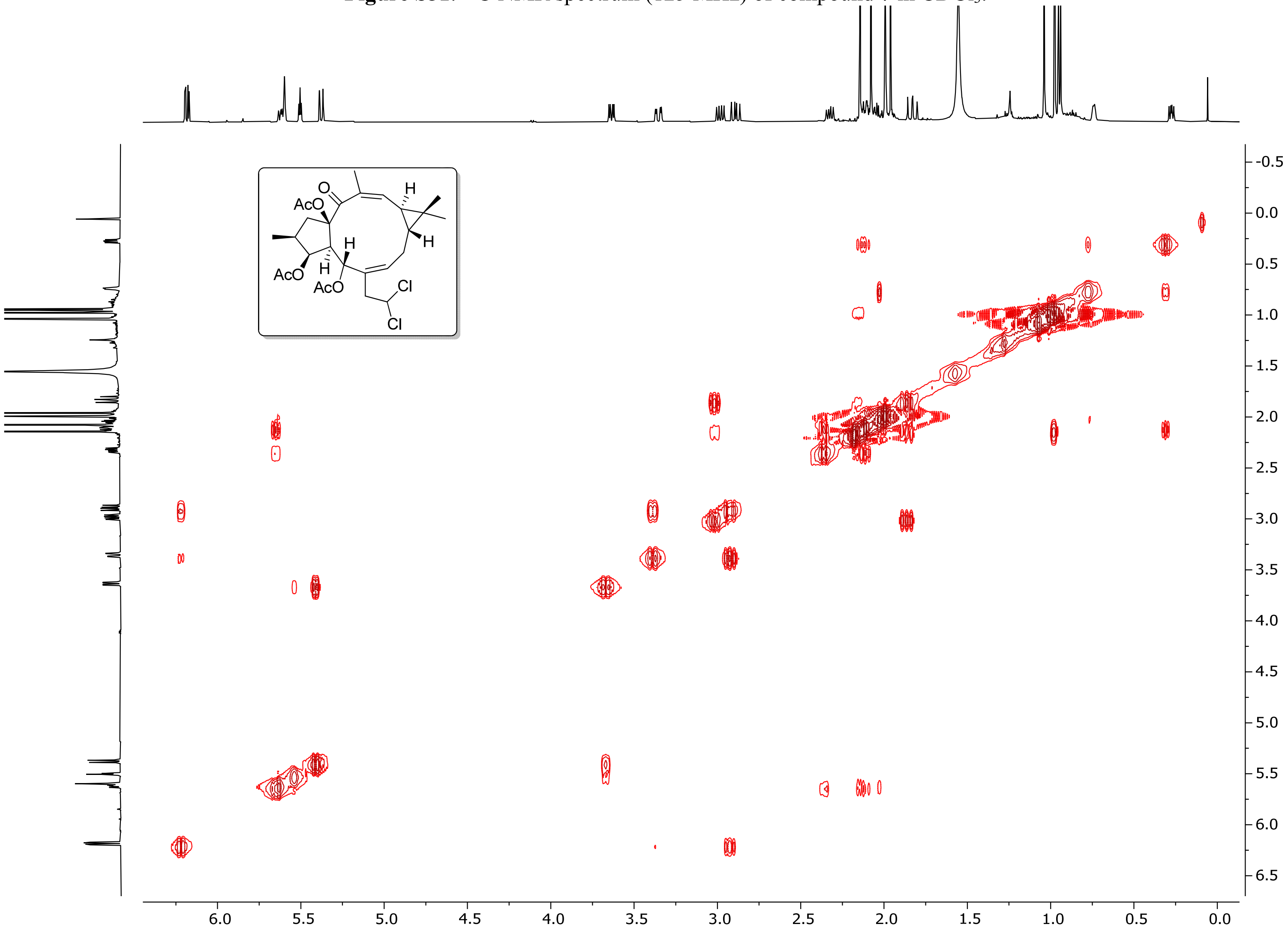

Figure S52. gCOSY spectrum of compound **7**.

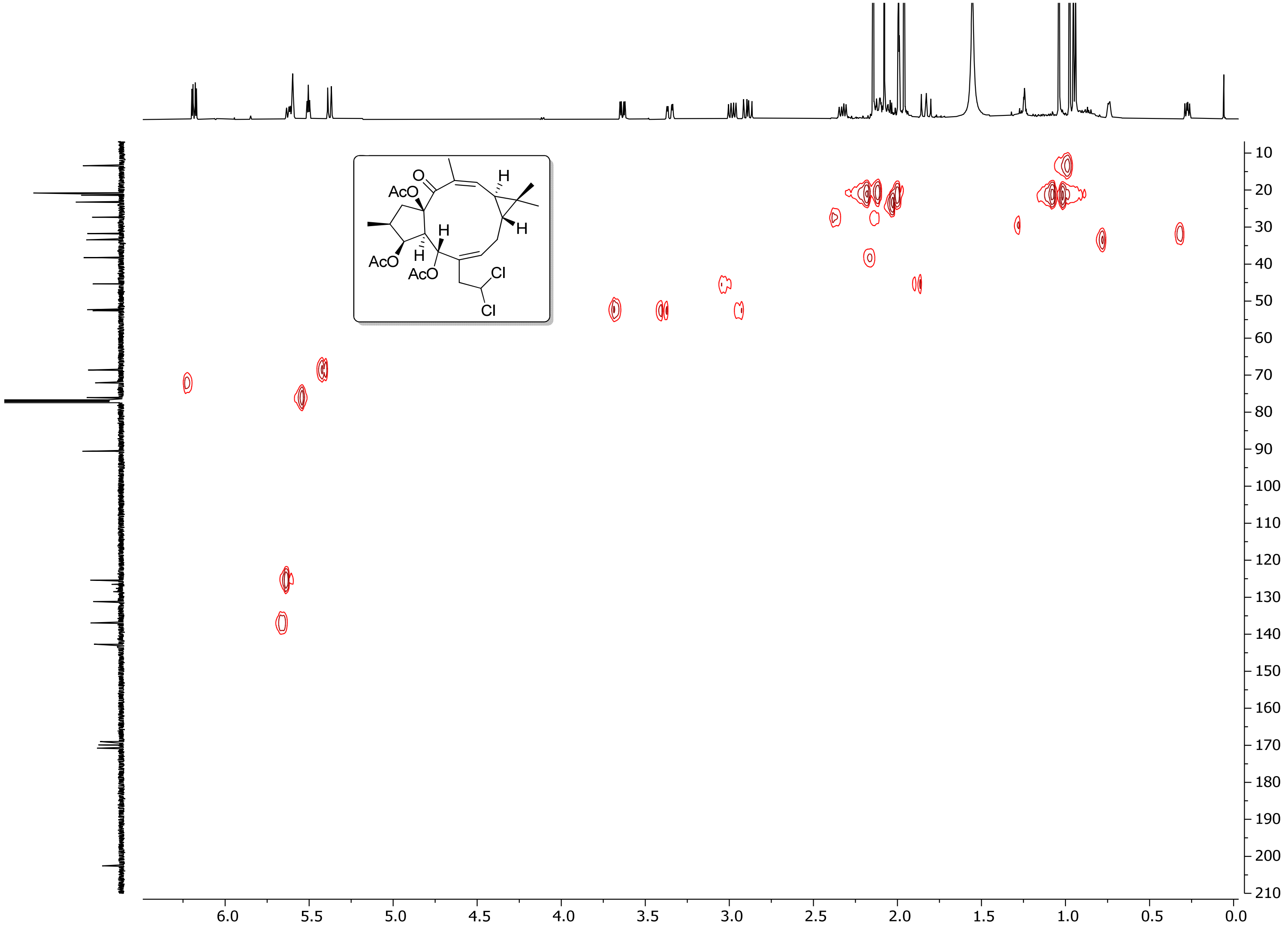

Figure S53. gHSQC spectrum of compound **7**.

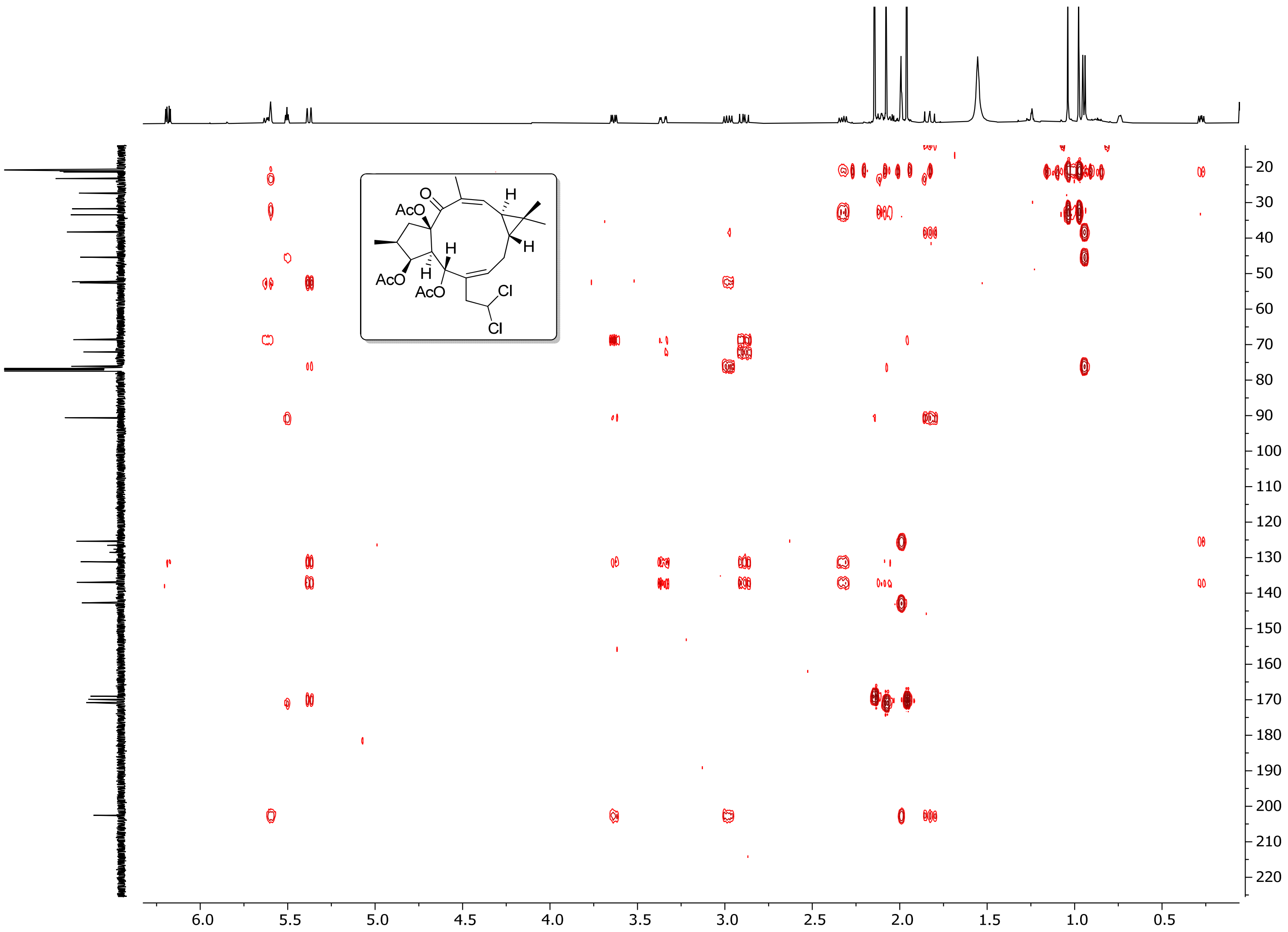

Figure S54. gHMBC spectrum of compound **7**.

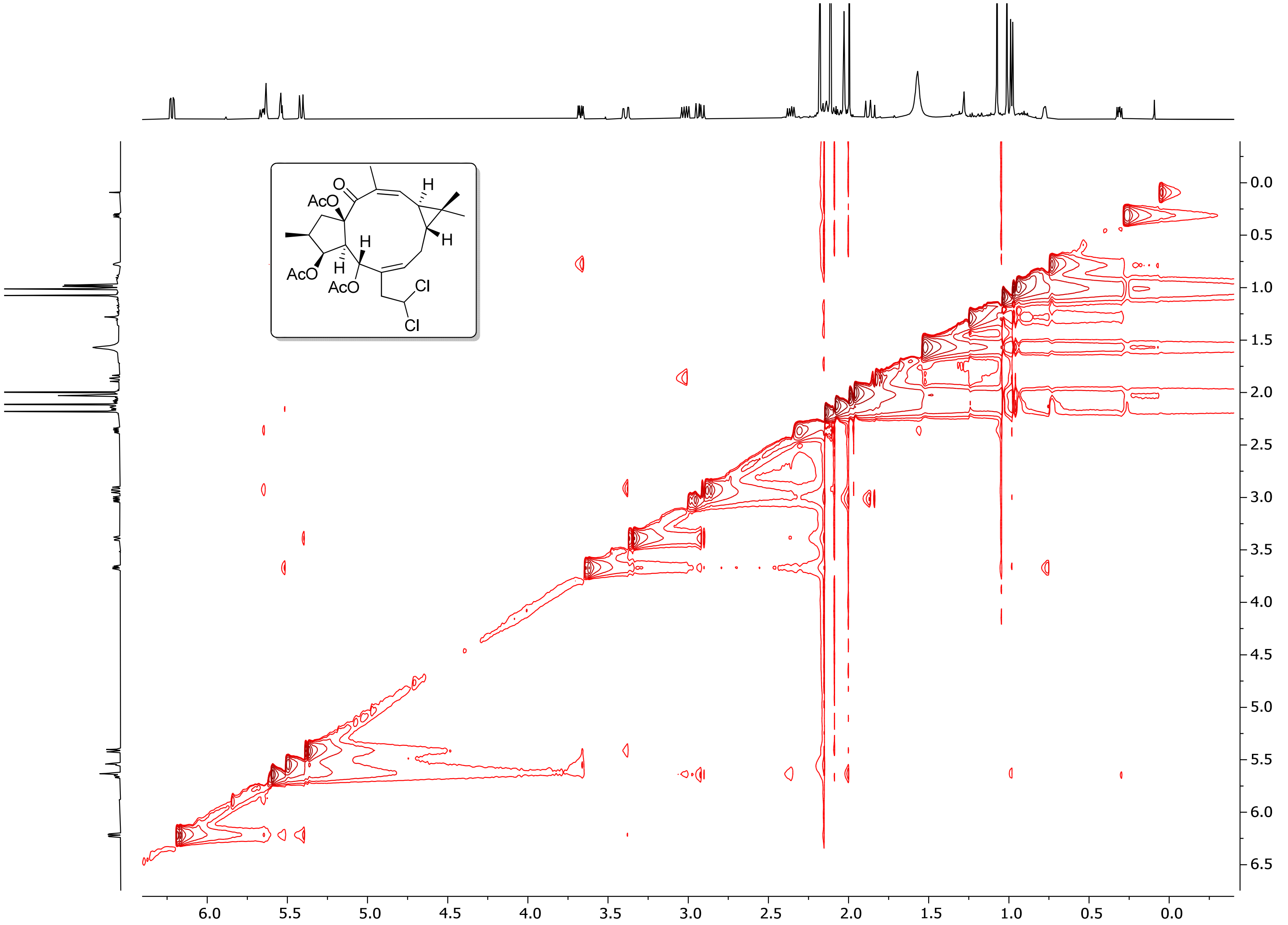

Figure S55. 2D NOESY spectrum of compound **7**.

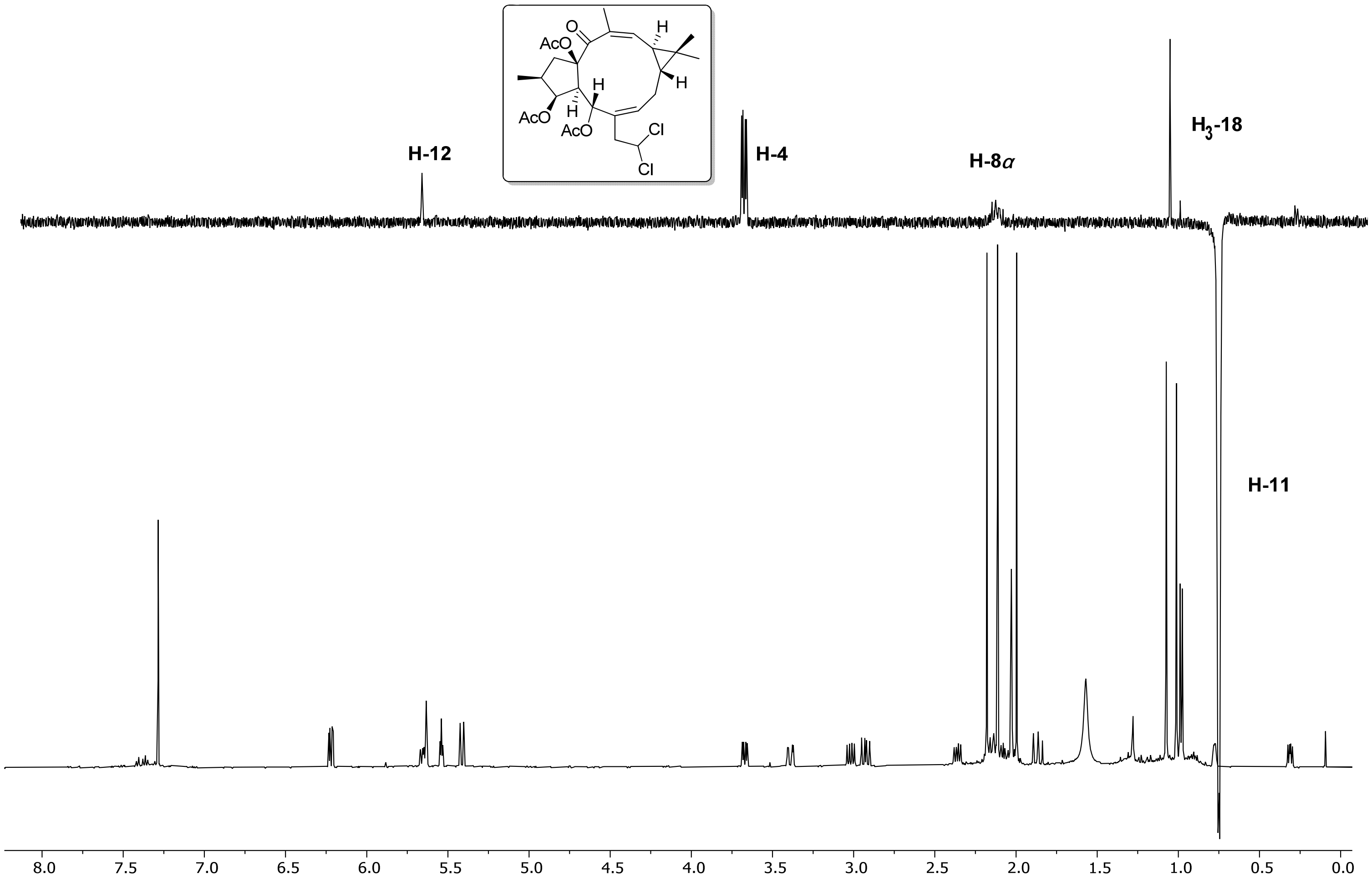

Figure S56a. 1D NOESY spectrum of compound **7**.

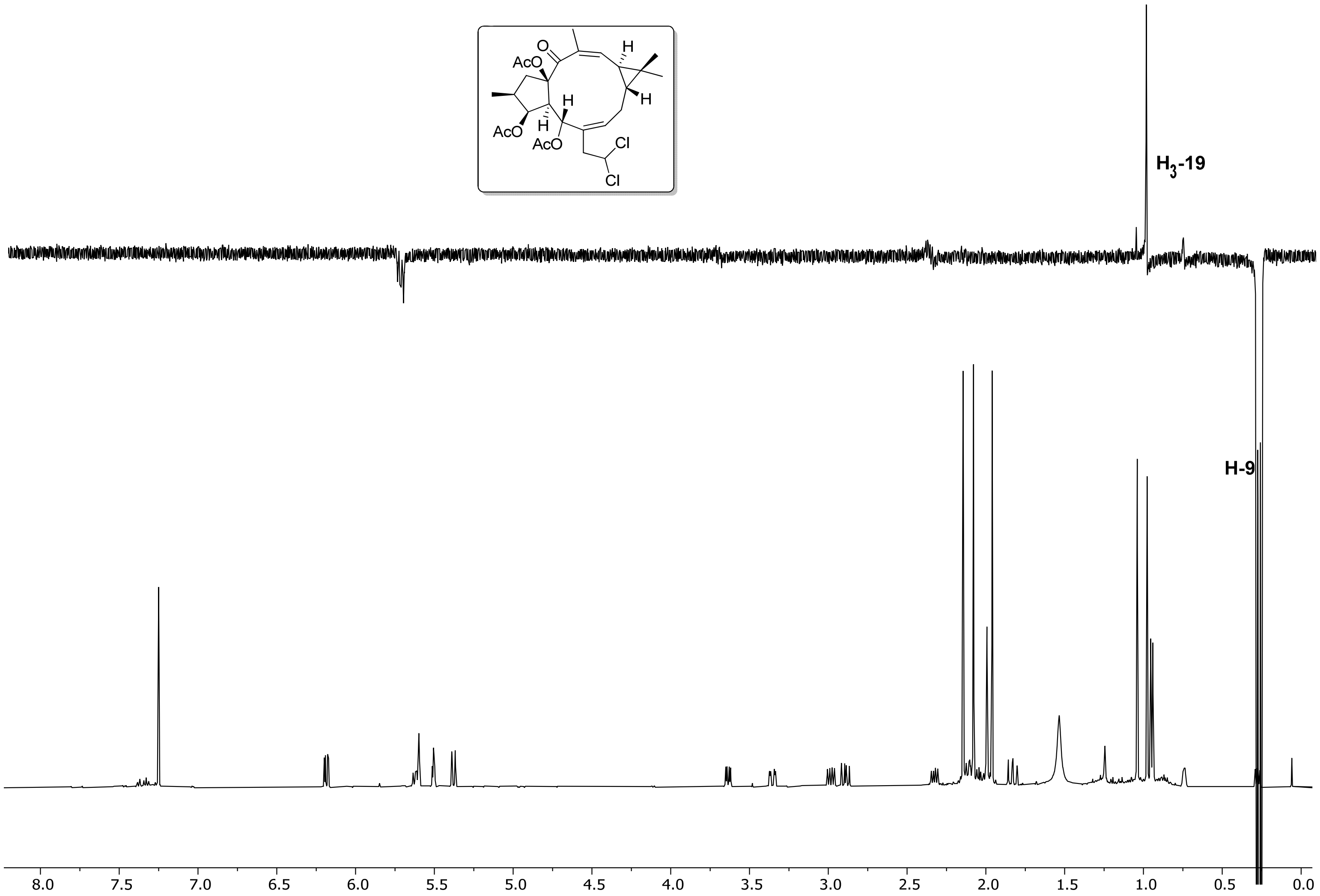

Figure S56b. 1D NOESY spectrum of compound **7**.

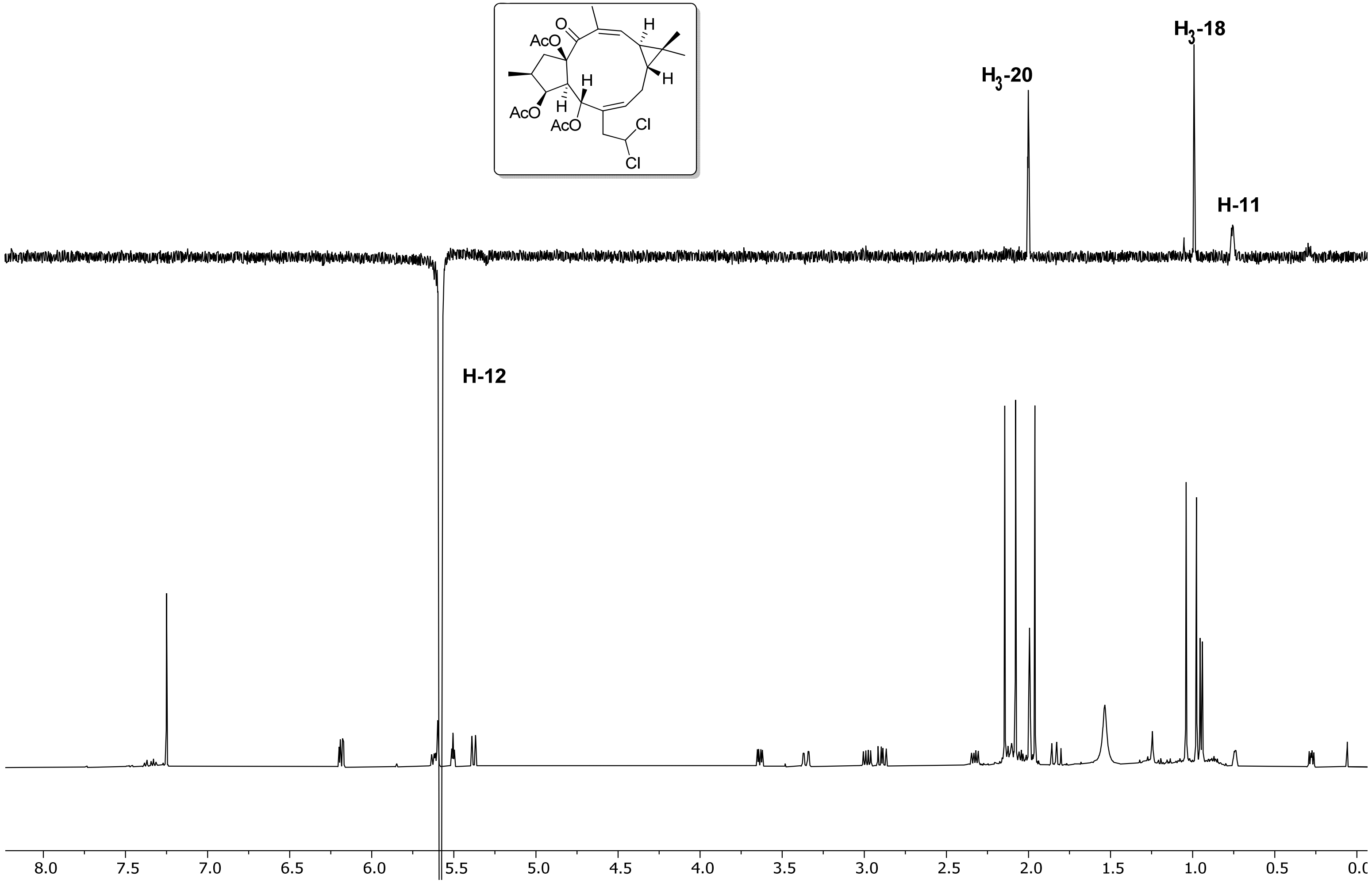

Figure S56c. 1D NOESY spectrum of compound **7**.

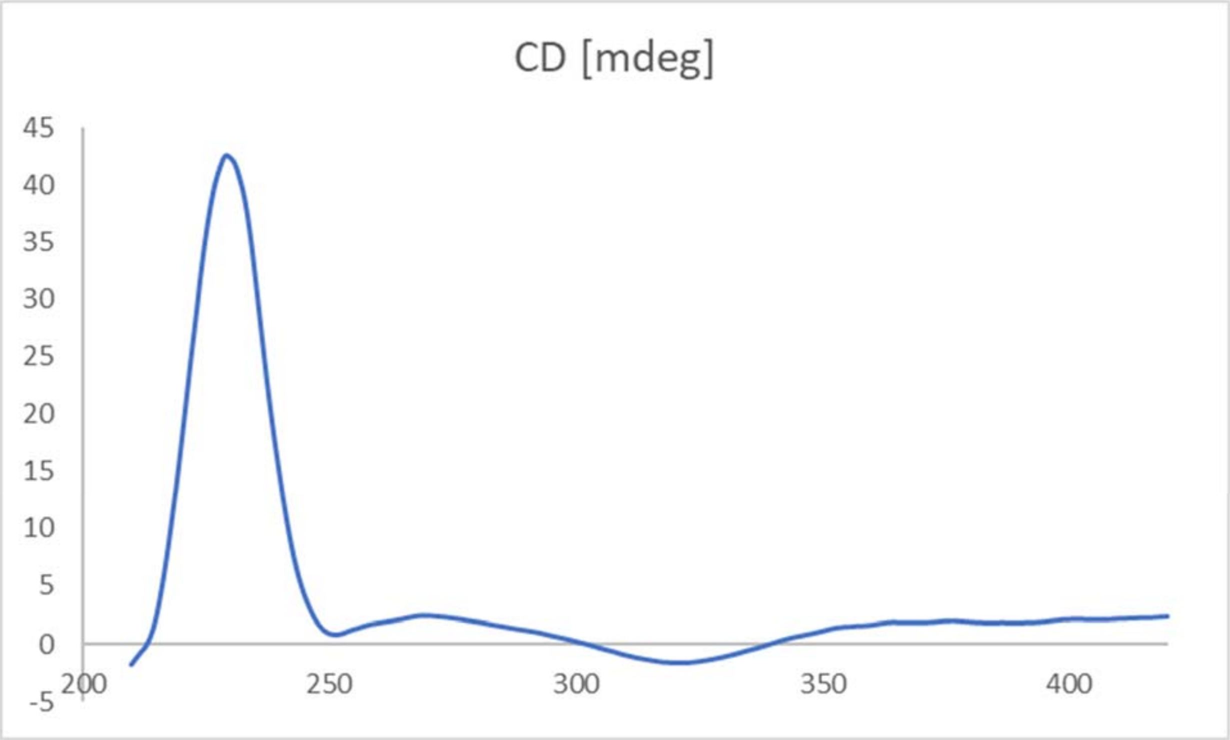

Figure S57. ECD spectrum of compound **7**.

Elemental Composition Report

Single Mass Analysis
Tolerance = 5.0 mDa / DBE: min = -1.5, max = 50.0
Element prediction: Off
Number of isotope peaks used for i-FIT = 3

Monoisotopic Mass, Even Electron Ions
1592 formula(e) evaluated with 16 results within limits (up to 50 best isotopic matches for each mass)
Elements Used:
C: 0-500 H: 0-1000 O: 0-200 Na: 0-1 Cl: 0-8
EB12--Benz-3--MSe2pos 451 (3.654)
2: TOF MS ES+

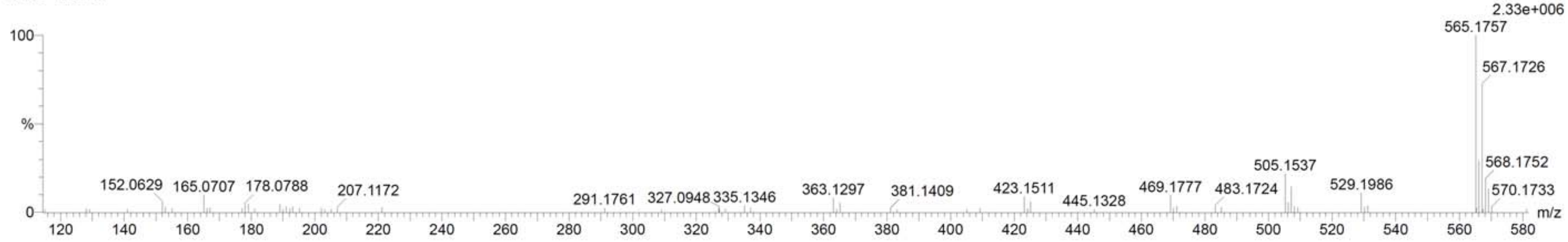

Minimum:				-1.5				
Maximum:		5.0	10.0	50.0				

Mass	Calc. Mass	mDa	PPM	DBE	i-FIT	Norm	Conf(%)	Formula
565.1757	565.1736	2.1	3.7	8.5	70.7	0.096	90.81	C27 H36 O7 Na Cl2
	565.1760	-0.3	-0.5	11.5	73.0	2.404	9.03	C29 H35 O7 Cl2
	565.1795	-3.8	-6.7	-0.5	77.2	6.690	0.12	C20 H40 O12 Na Cl2
	565.1738	1.9	3.4	2.5	78.9	8.374	0.02	C23 H40 O9 Cl3
	565.1714	4.3	7.6	-0.5	80.3	9.762	0.01	C21 H41 O9 Na Cl3
	565.1747	1.0	1.8	-1.5	80.8	10.283	0.00	C17 H38 O18 Cl
	565.1758	-0.1	-0.2	17.5	81.8	11.196	0.00	C33 H31 O5 Na Cl
	565.1786	-2.9	-5.1	3.5	82.0	11.444	0.00	C26 H42 O3 Na Cl4
	565.1729	2.8	5.0	6.5	82.4	11.835	0.00	C29 H42 Cl5
	565.1782	-2.5	-4.4	20.5	82.8	12.285	0.00	C35 H30 O5 Cl
	565.1723	3.4	6.0	29.5	83.4	12.882	0.00	C42 H26 Cl
	565.1745	1.2	2.1	4.5	90.6	20.030	0.00	C21 H34 O16 Na
	565.1769	-1.2	-2.1	7.5	90.6	20.067	0.00	C23 H33 O16
	565.1710	4.7	8.3	16.5	90.8	20.219	0.00	C30 H29 O11
	565.1780	-2.3	-4.1	26.5	91.2	20.626	0.00	C39 H26 O3 Na
	565.1804	-4.7	-8.3	29.5	91.3	20.720	0.00	C41 H25 O3

Figure S58. HRMS spectrum of compound **7**.

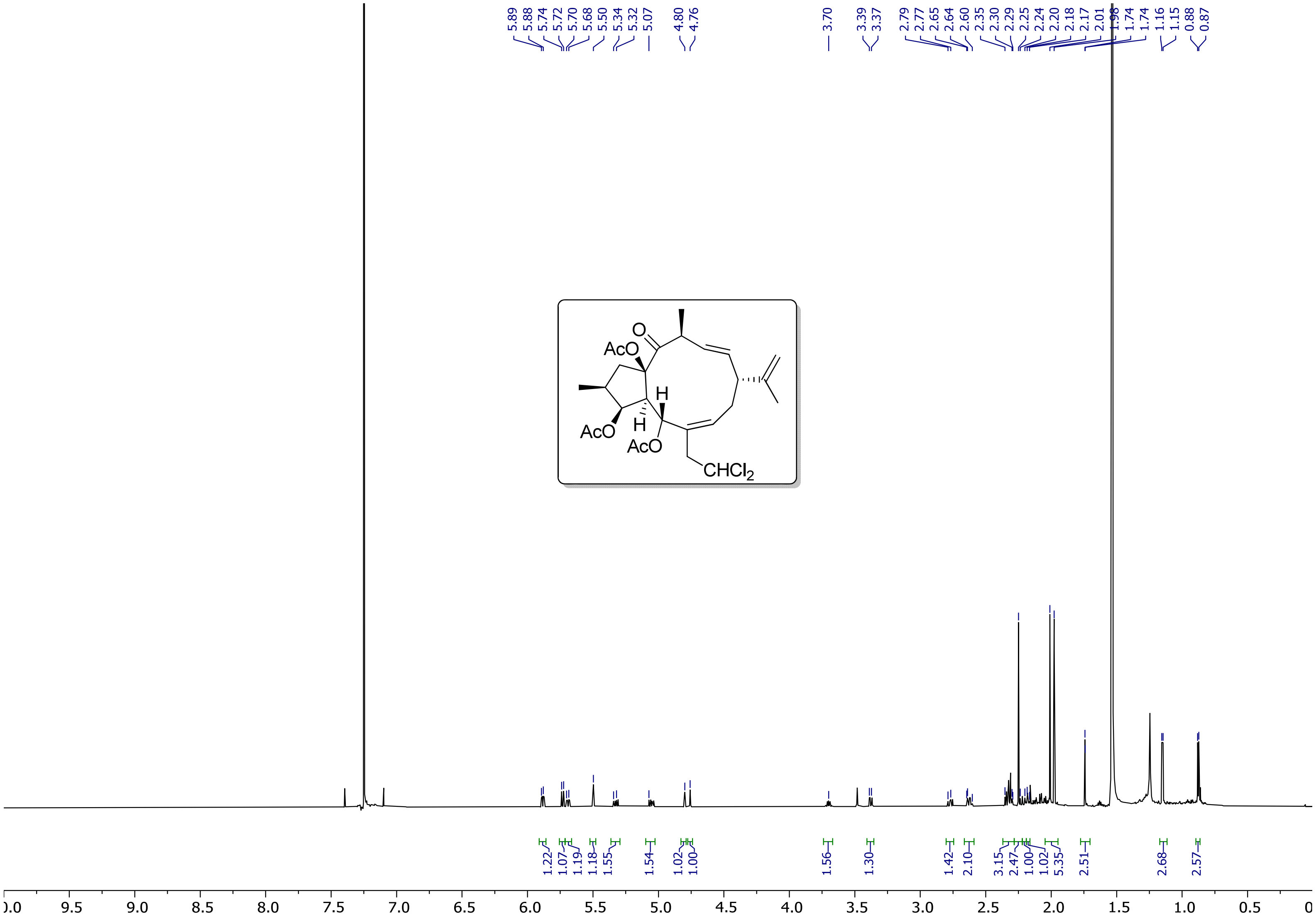

Figure S59. ^{1}H NMR spectrum (700 MHz) of compound **8** in $CDCl_3$.

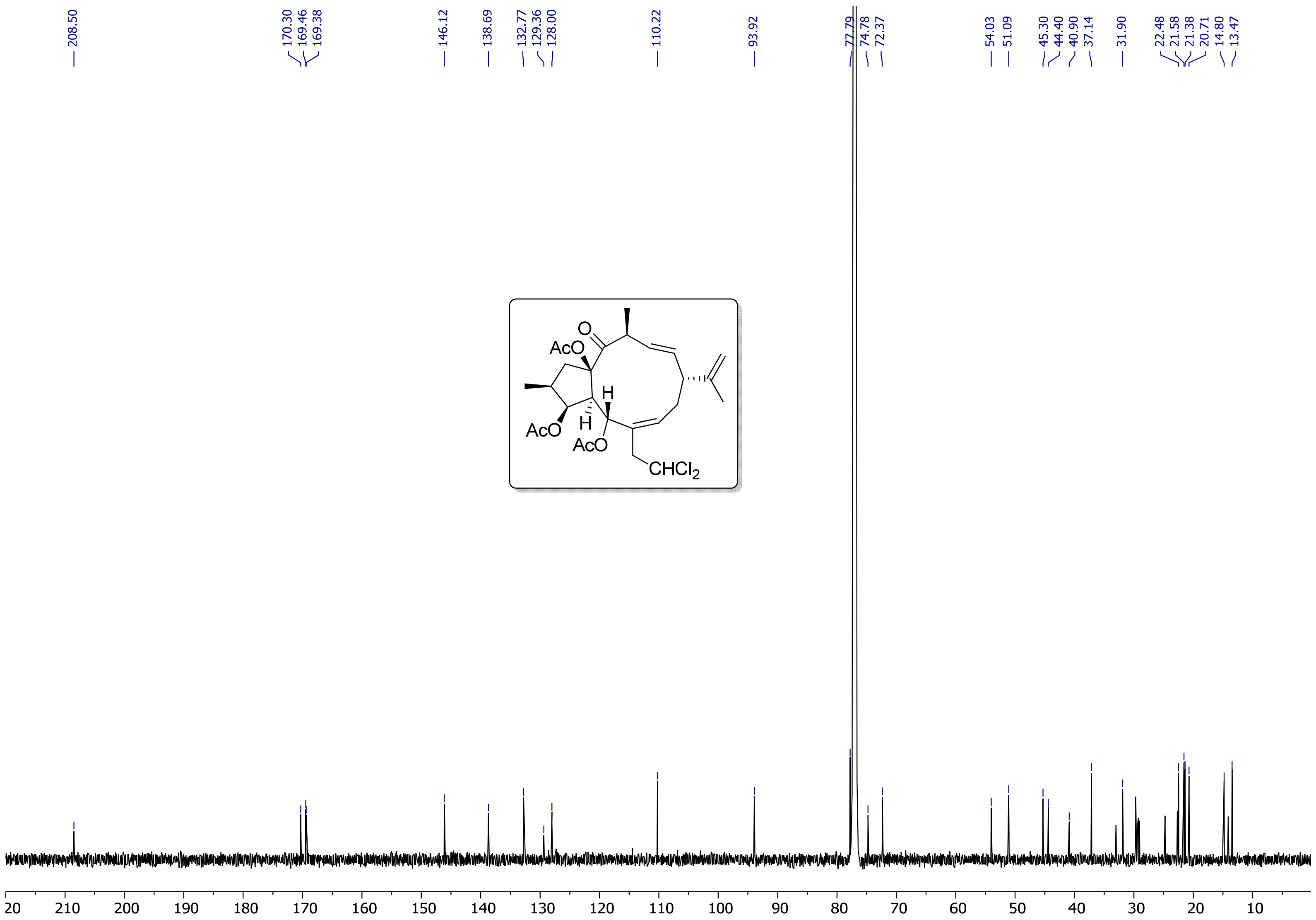

Figure S60. ^{13}C NMR spectrum (175 MHz) of compound **8** in $CDCl_3$.

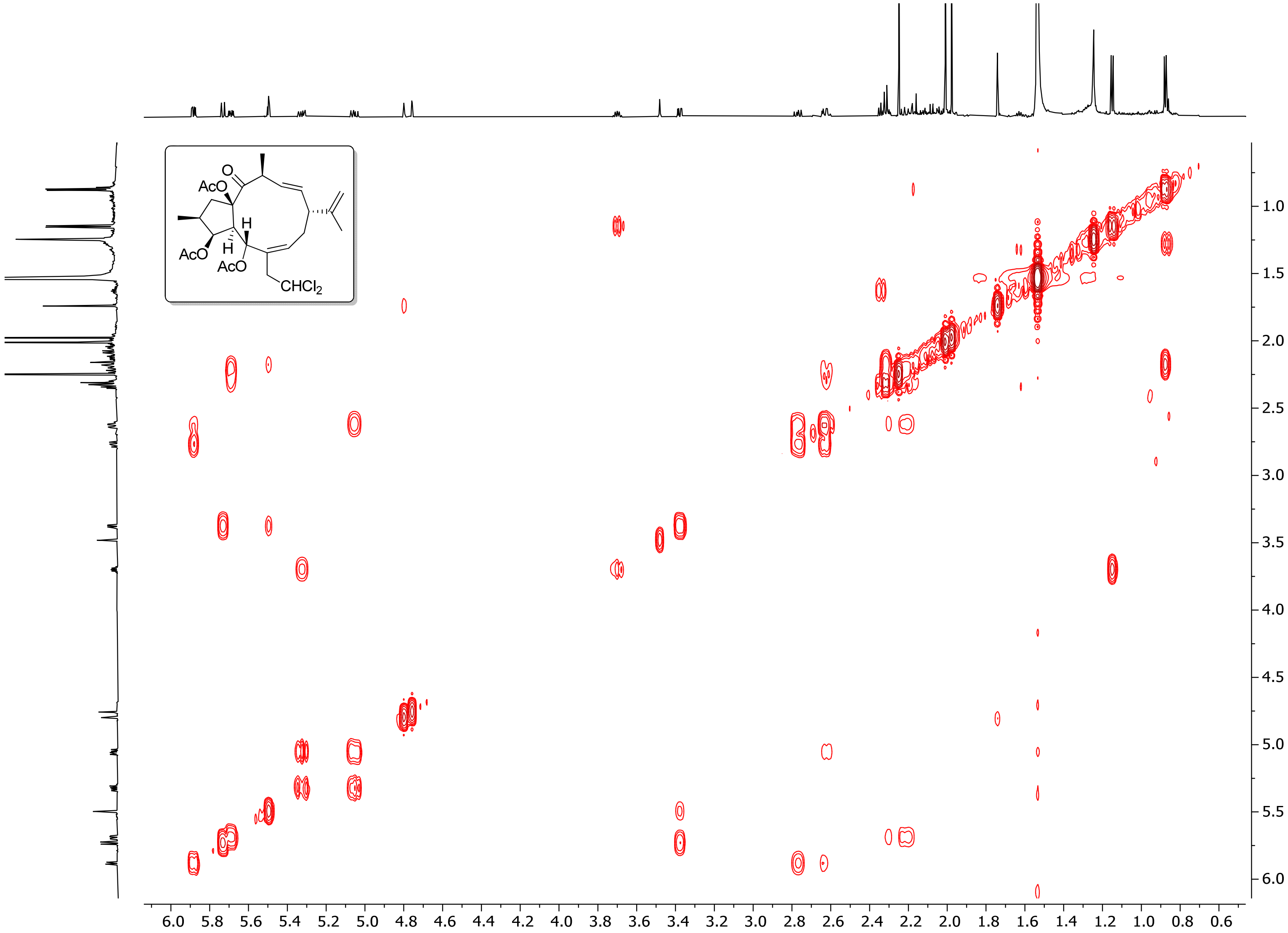

Figure S61. gCOSY spectrum of compound **8**.

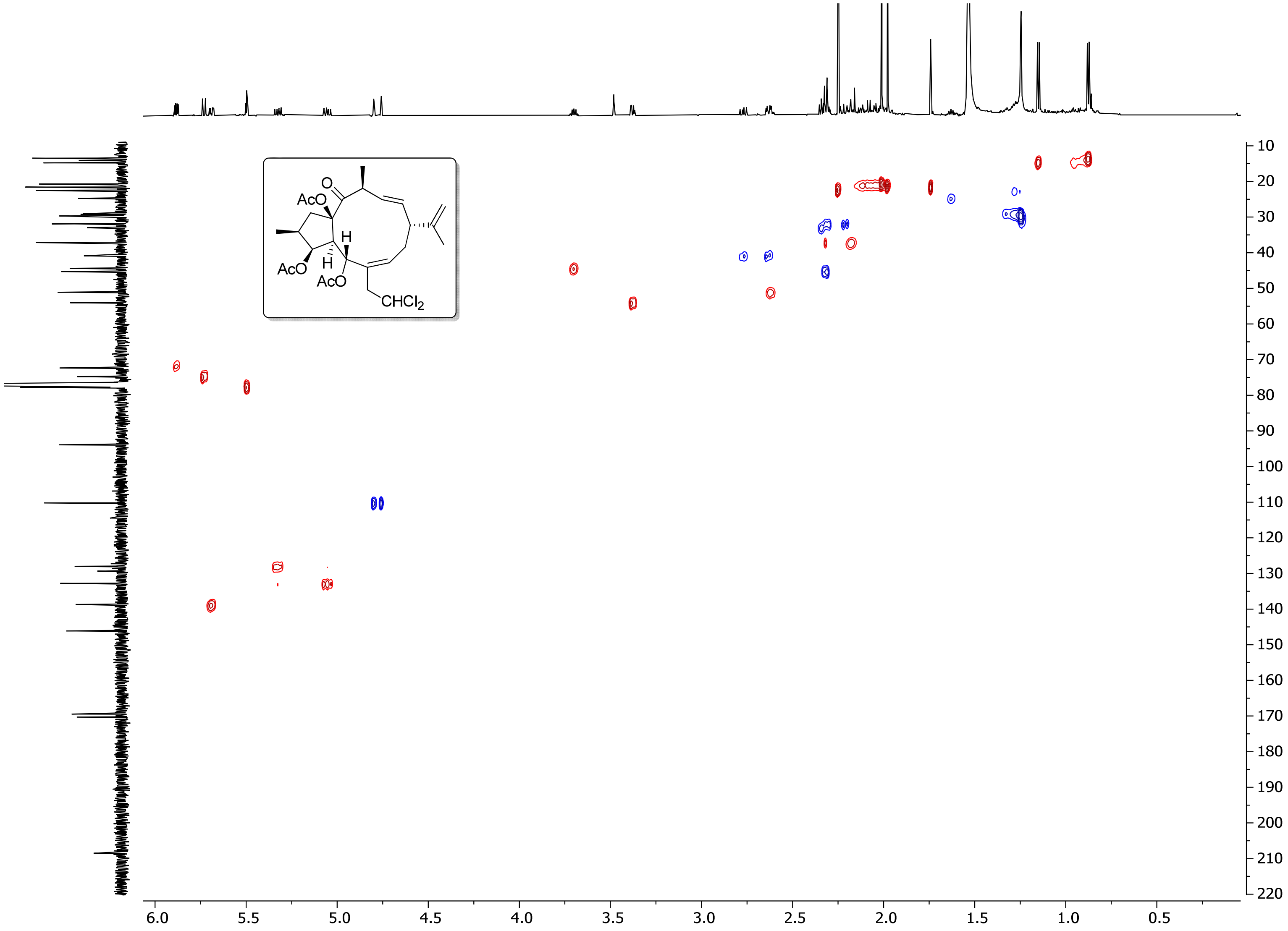

Figure S62. gHSQC spectrum of compound **8**.

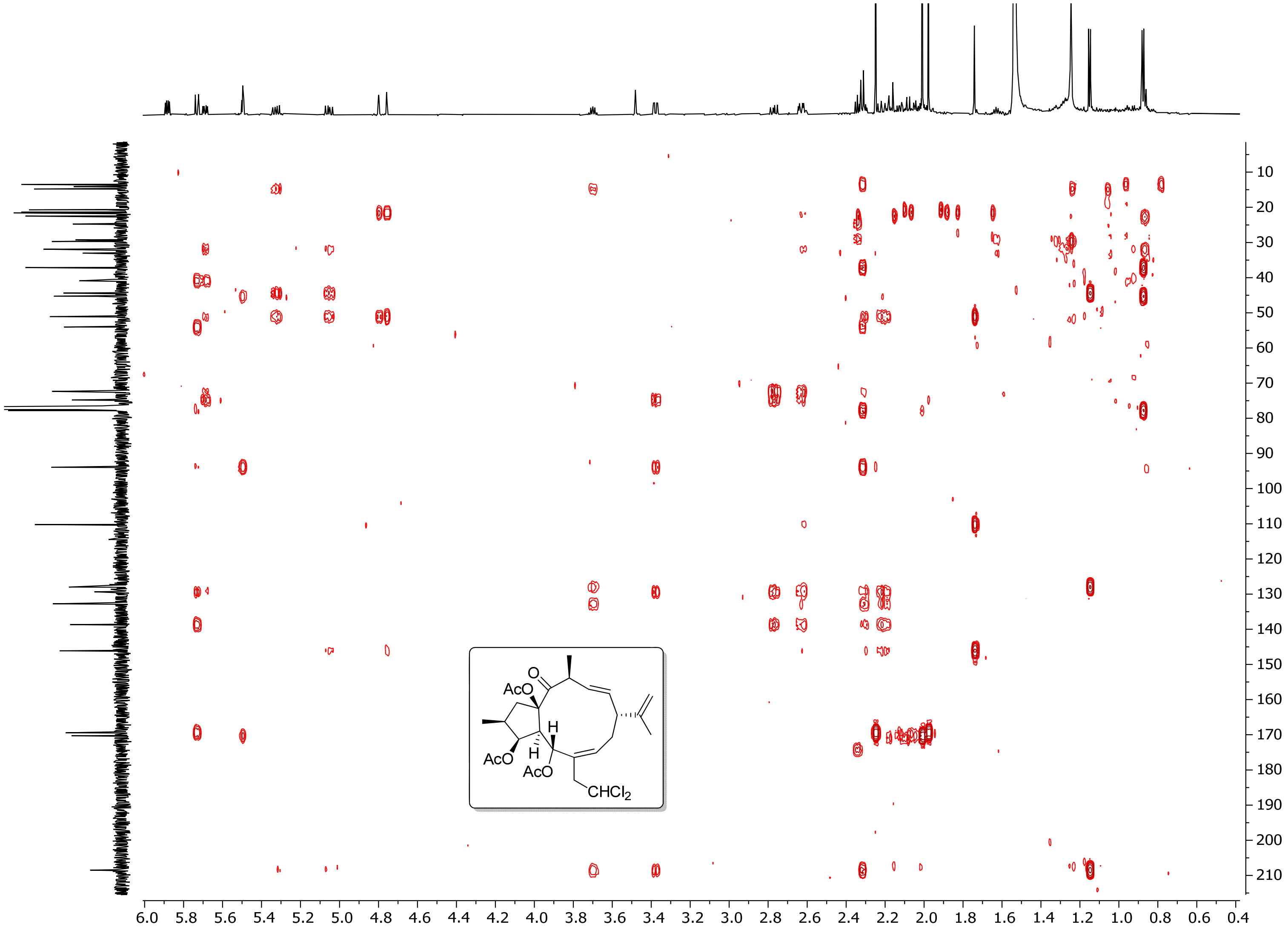

Figure S63. gHMBC spectrum of compound **8**.

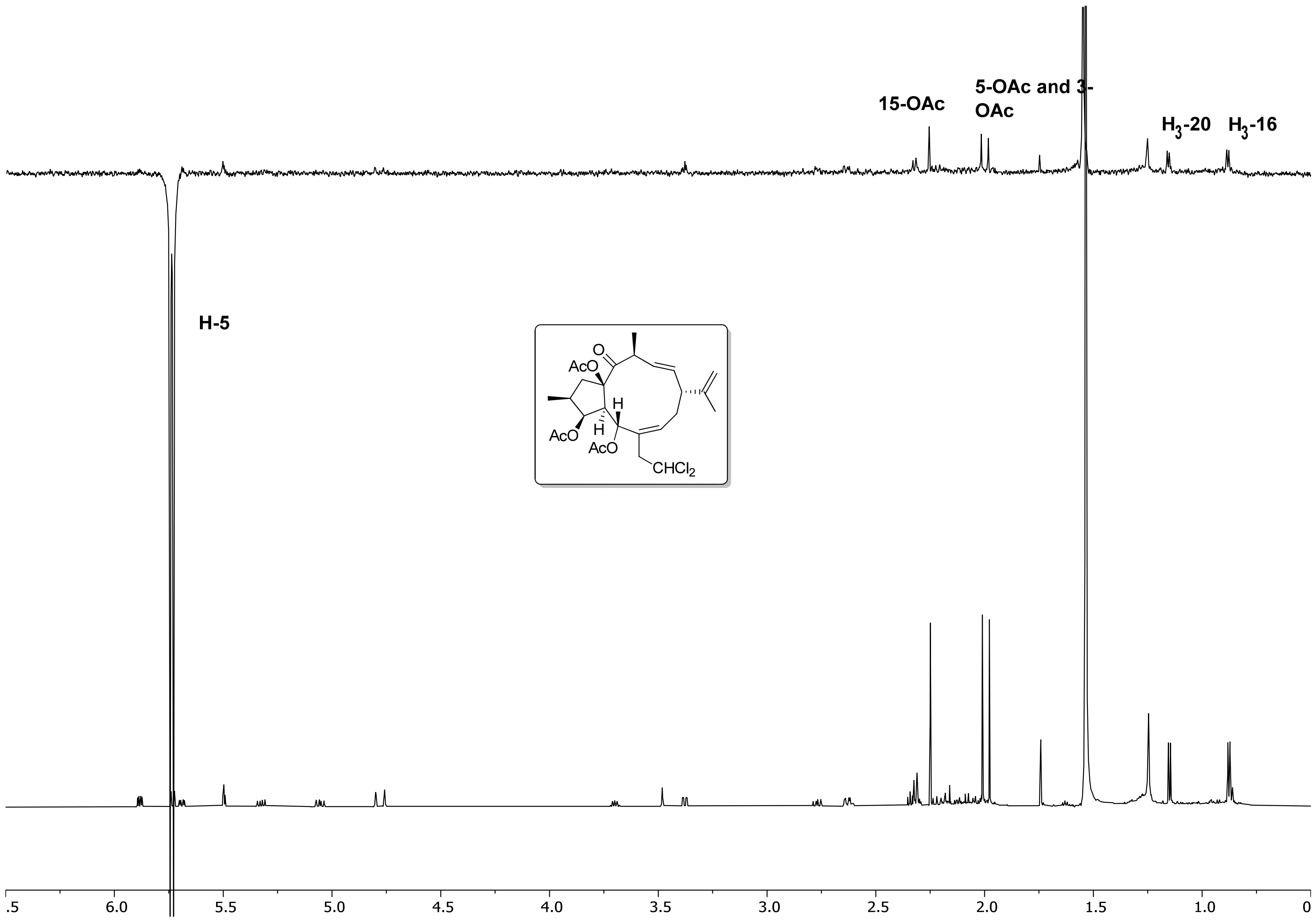

Figure S64a. 1D NOESY spectrum of compound **8**.

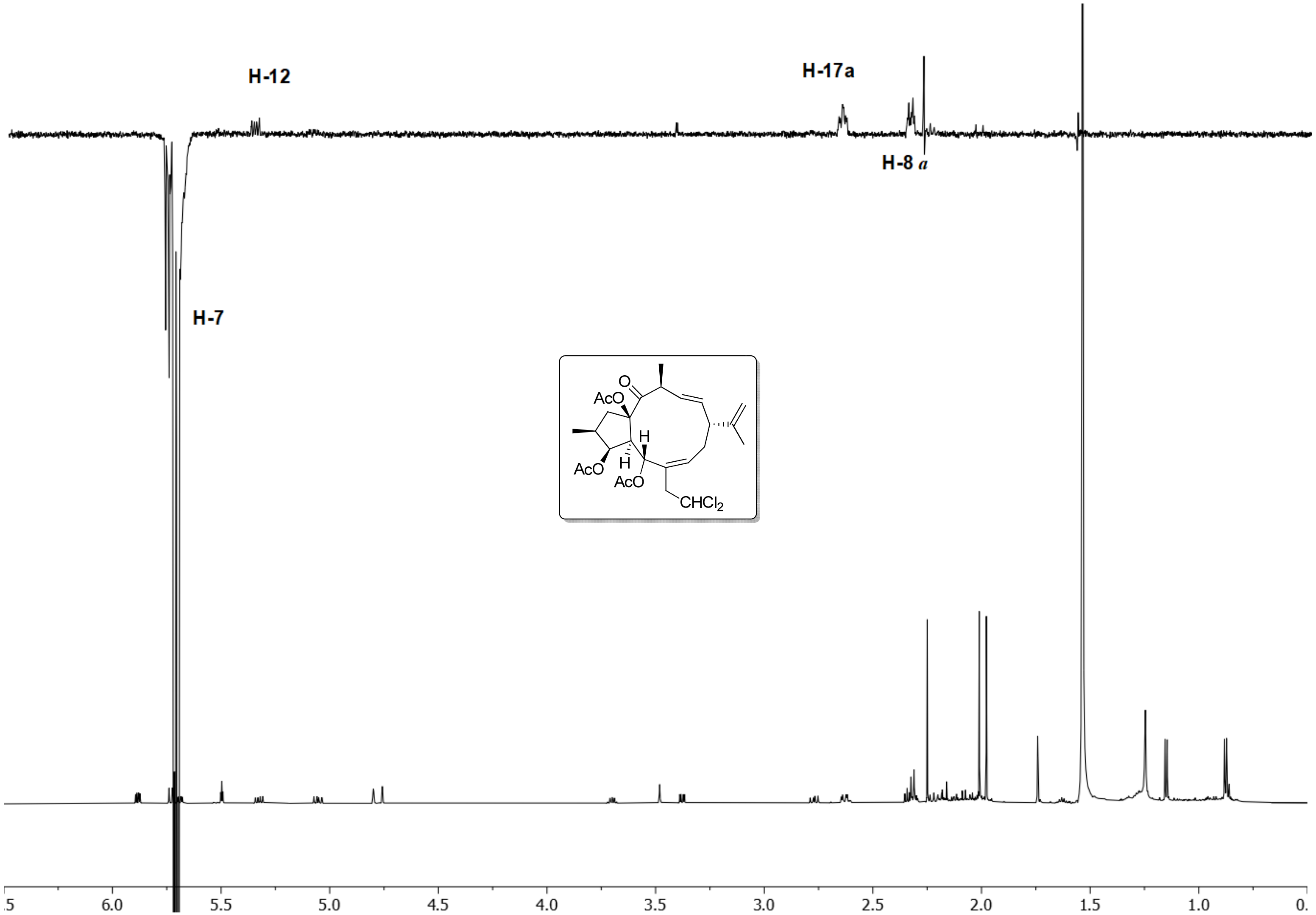

Figure S64b. 1D NOESY spectrum of compound **8**.

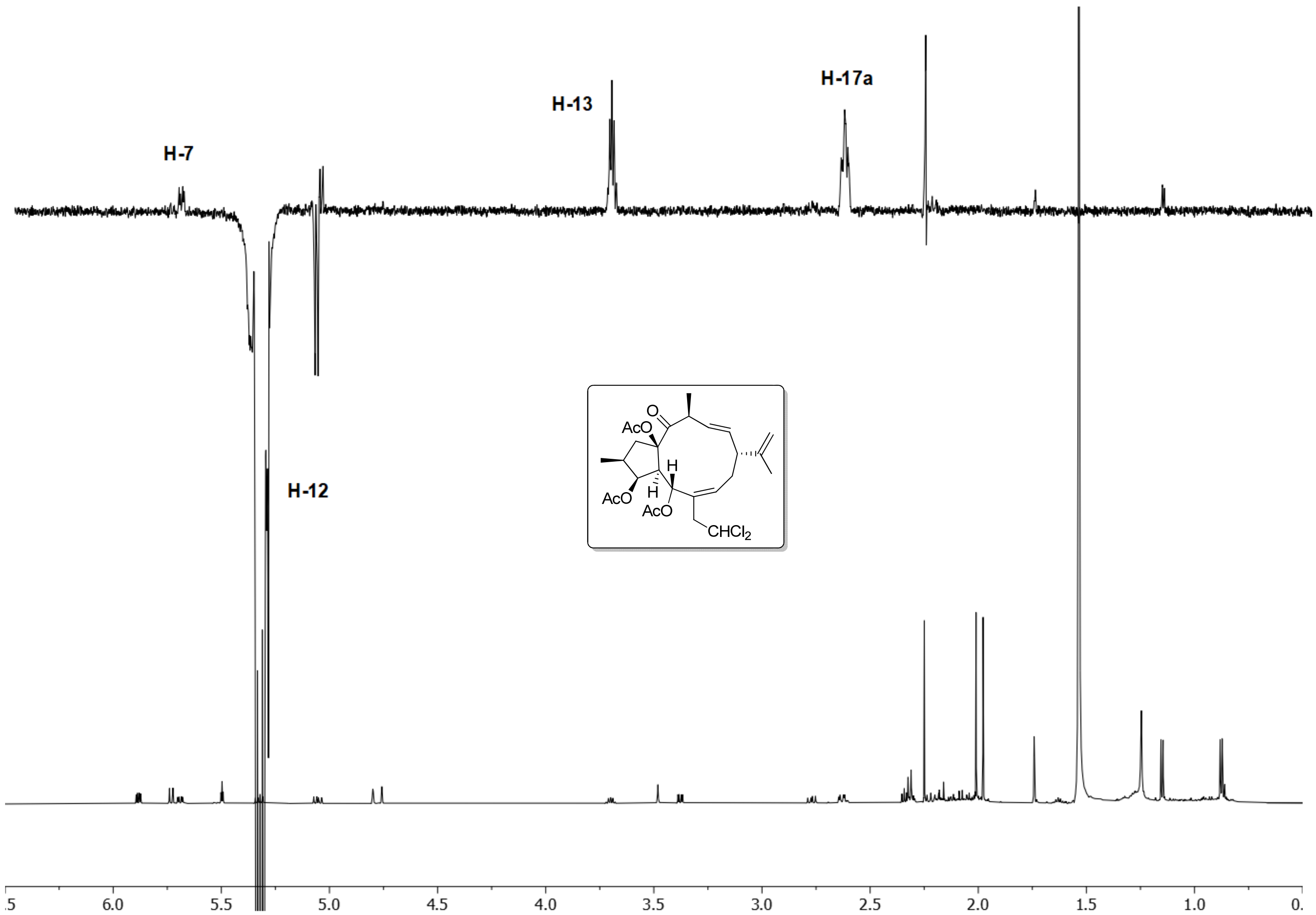

Figure S64c. 1D NOESY spectrum of compound **8**.

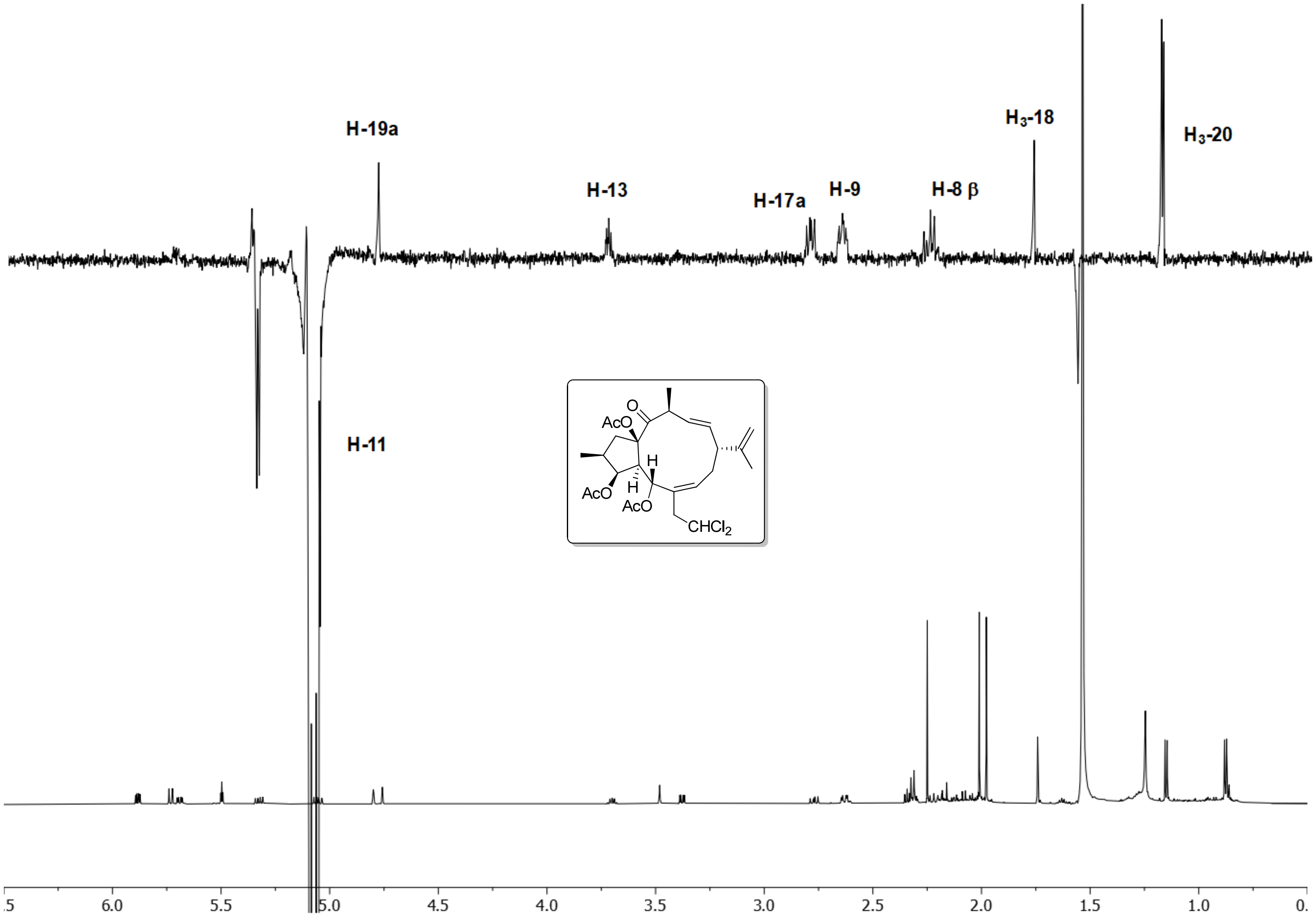

Figure S64d. 1D NOESY spectrum of compound **8**.

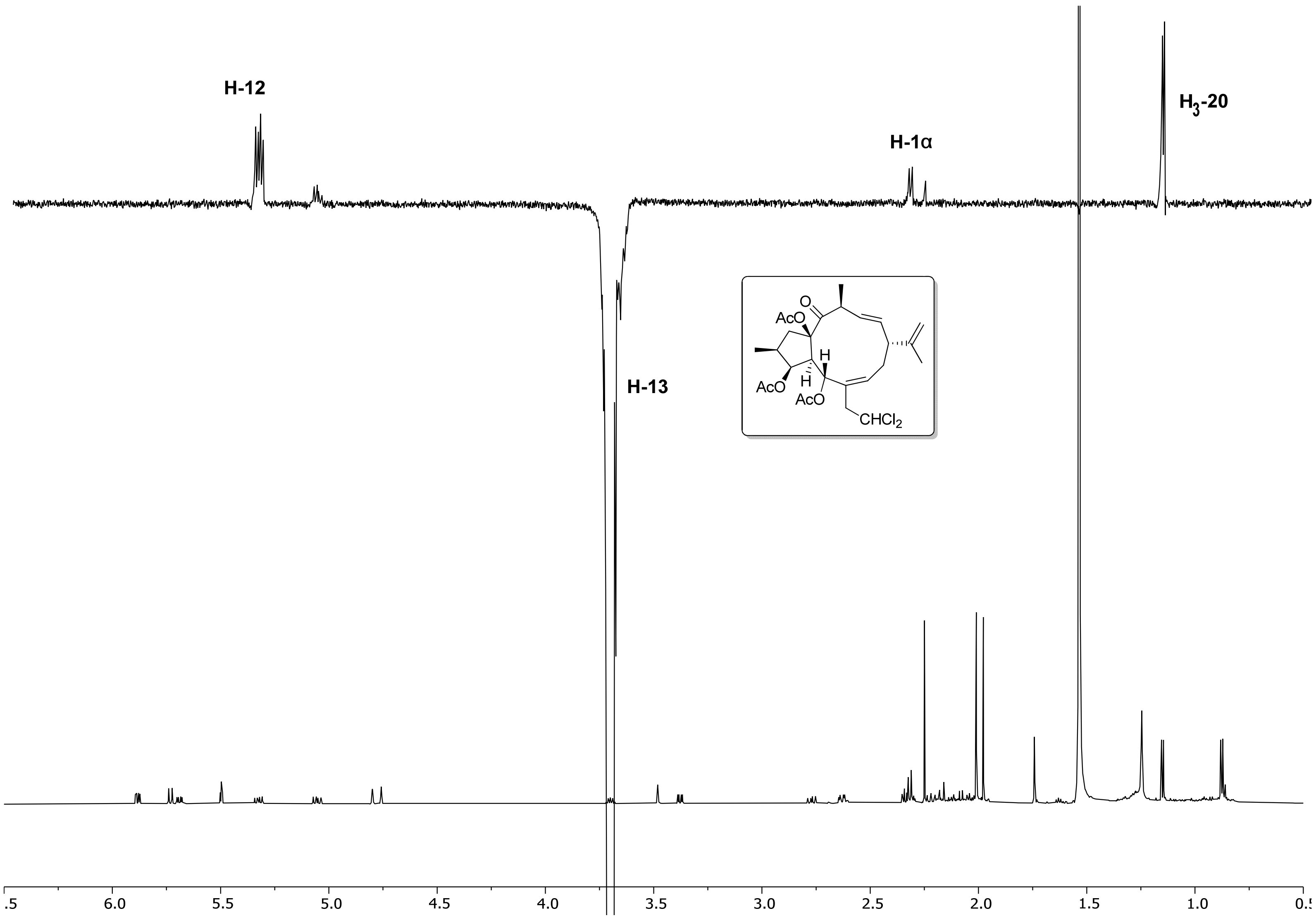

Figure S64e. 1D NOESY spectrum of compound **8**.

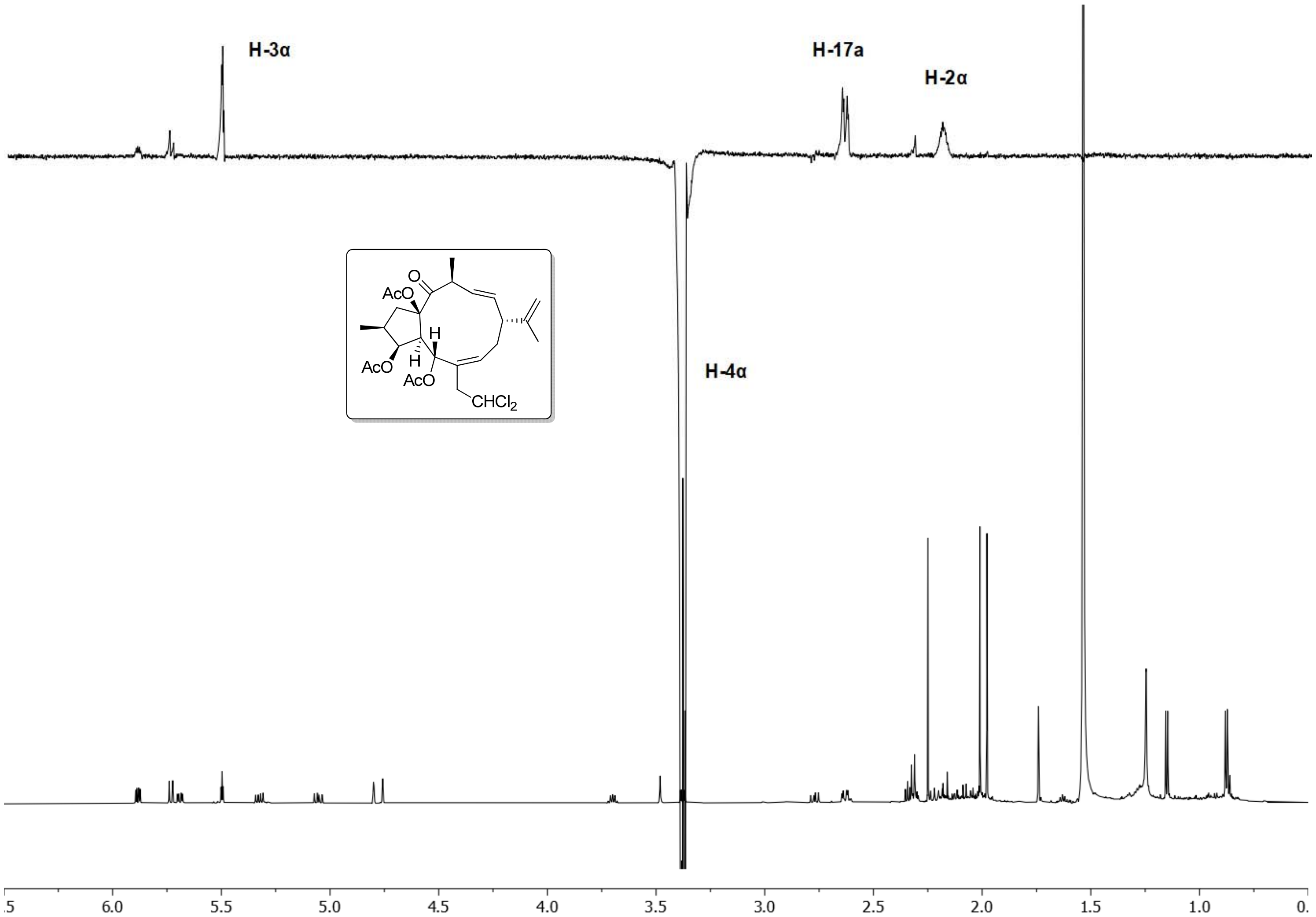

Figure S64f. 1D NOESY spectrum of compound **8**.

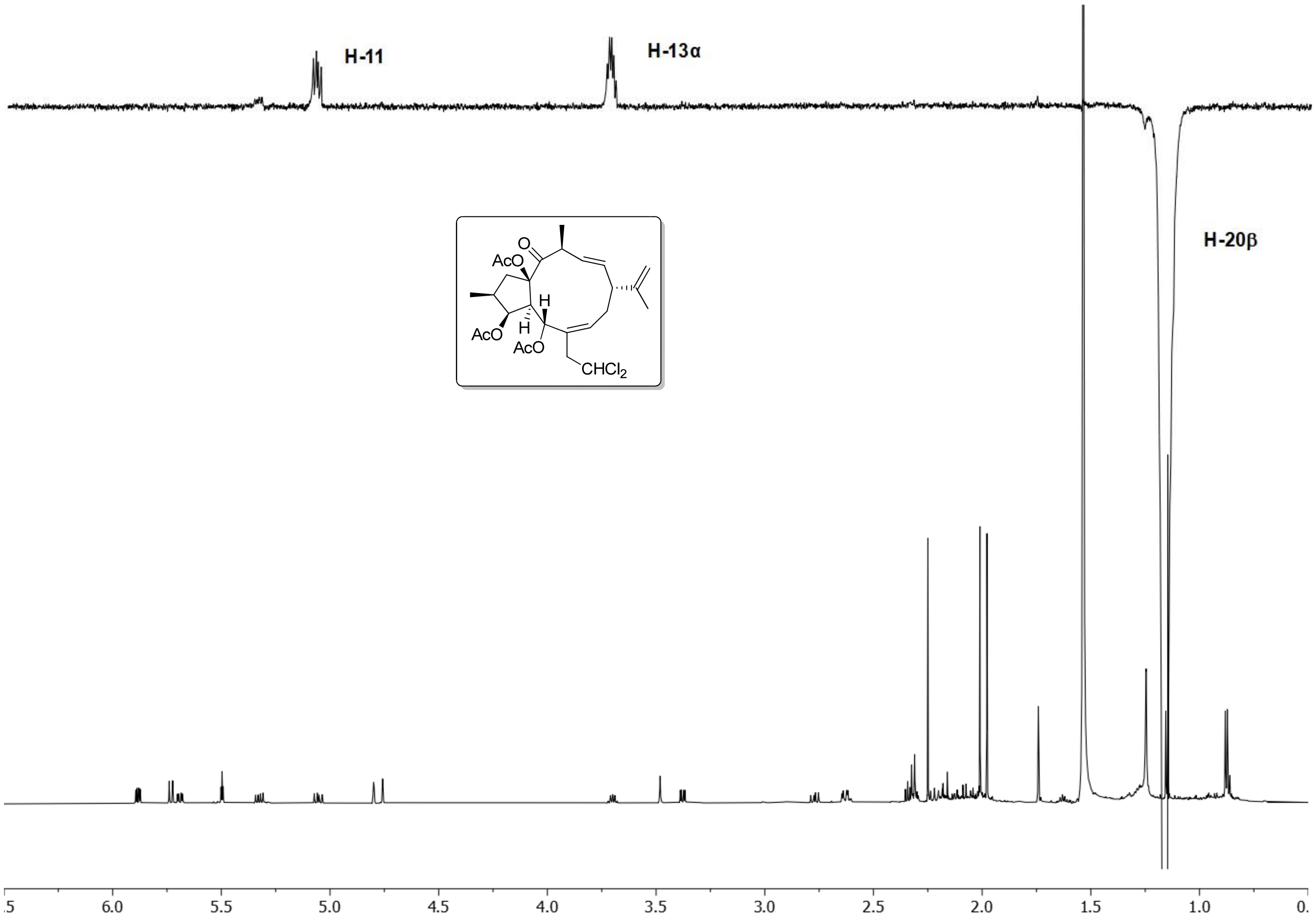

Figure S64g. 1D NOESY spectrum of compound **8**.

Elemental Composition Report **Page 1**

Single Mass Analysis
Tolerance = 5.0 mDa / DBE: min = -1.5, max = 50.0
Element prediction: Off
Number of isotope peaks used for i-FIT = 3

Monoisotopic Mass, Even Electron Ions
1592 formula(e) evaluated with 17 results within limits (up to 50 closest results for each mass)
Elements Used:
C: 0-300 H: 0-100 O: 0-200 23Na: 0-1 Cl: 0-8
HPLC12--MSe2pos 472 (3.804)
2: TOF MS ES+

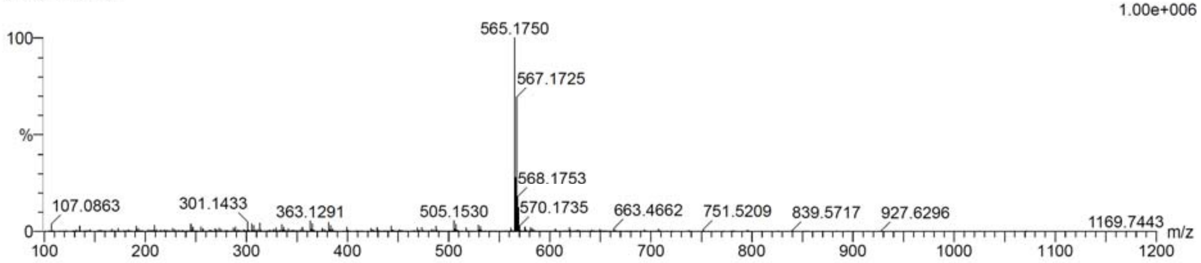

Minimum:				-1.5				
Maximum:		5.0	10.0	50.0				
Mass	Calc. Mass	mDa	PPM	DBE	i-FIT	Norm	Conf(%)	Formula
565.1750	565.1747	0.3	0.5	-1.5	818.0	9.899	0.01	C17 H38 O18 Cl
	565.1745	0.5	0.9	4.5	829.7	21.683	0.00	C21 H34 O16 23Na
	565.1758	-0.8	-1.4	17.5	819.7	11.686	0.00	C33 H31 O5 23Na Cl
	565.1760	-1.0	-1.8	11.5	811.1	3.024	4.86	C29 H35 O7 Cl2
	565.1738	1.2	2.1	2.5	816.5	8.491	0.02	C23 H40 O9 Cl3
	565.1736	1.4	2.5	8.5	808.1	0.051	95.02	C27 H36 O7 23Na Cl2
	565.1769	-1.9	-3.4	7.5	829.8	21.730	0.00	C23 H33 O16
	565.1729	2.1	3.7	6.5	820.1	12.047	0.00	C29 H42 Cl5
	565.1723	2.7	4.8	29.5	822.0	13.942	0.00	C42 H26 Cl
	565.1780	-3.0	-5.3	26.5	830.4	22.353	0.00	C39 H26 O3 23Na
	565.1782	-3.2	-5.7	20.5	821.4	13.312	0.00	C35 H30 O5 Cl
	565.1786	-3.6	-6.4	3.5	820.4	12.380	0.00	C26 H42 O3 23Na Cl4
	565.1714	3.6	6.4	-0.5	818.6	10.510	0.00	C21 H41 O9 23Na Cl3
	565.1710	4.0	7.1	16.5	830.0	21.900	0.00	C30 H29 O11
	565.1705	4.5	8.0	3.5	822.2	14.115	0.00	C27 H43 23Na Cl5
	565.1795	-4.5	-8.0	-0.5	815.1	7.032	0.09	C20 H40 O12 23Na Cl2
	565.1701	4.9	8.7	20.5	817.7	9.689	0.01	C36 H31 O2 Cl2

Figure S65. HRMS spectrum of compound **8**.
